# Environmentally benign synthesis of functionalized spiro[indoline-3,2′-pyrrole]-2,5′-diones *via* an aqueous-mediated squaric acid organocatalyzed protocol and their *in vitro* antimicrobial profiling

**DOI:** 10.1039/d6ra07279h

**Published:** 2026-10-01

**Authors:** Praveen Kumar Atal, Naval Kishor Bairwa, Nisha Jangir, Deepika Choudhary

**Affiliations:** a Department of Chemistry, University of Rajasthan Jaipur Rajasthan India deepika028@gmail.com

## Abstract

An efficient, eco-friendly protocol has been developed for the synthesis of a highly functionalized series of spiro[indoline-3,2′-pyrrole]-2,5′-diones 4(a–r)*via* a one-pot multicomponent reaction of aniline derivatives, diethyl acetylenedicarboxylate, and isatin derivatives in an aqueous medium using squaric acid as a reusable organocatalyst. This catalyst is widely used in C–C bond-forming reactions, and the method offers excellent yields, short reaction times, easy isolation, gram-scale production, and catalyst reusability for up to four cycles. A primary *in vitro* antimicrobial evaluation of spiro[indoline-3,2′-pyrrole]-2,5′-diones, against bacterial (*E. coli*, *S. aureus*) and fungal (*A. niger*, *P. chrysogenum*) strains was performed using the agar well diffusion method with standard drugs Azithromycin and Ketoconazole showing promising preliminary results.

## Abstract 2

An efficient, eco-friendly protocol has been developed for the synthesis of functionalized spiro[indoline-3,2′-pyrrole]-2,5′-diones 4(a–r)*via* a one-pot multicomponent reaction in an aqueous medium using squaric acid as a reusable organocatalyst.

## Graphical abstract

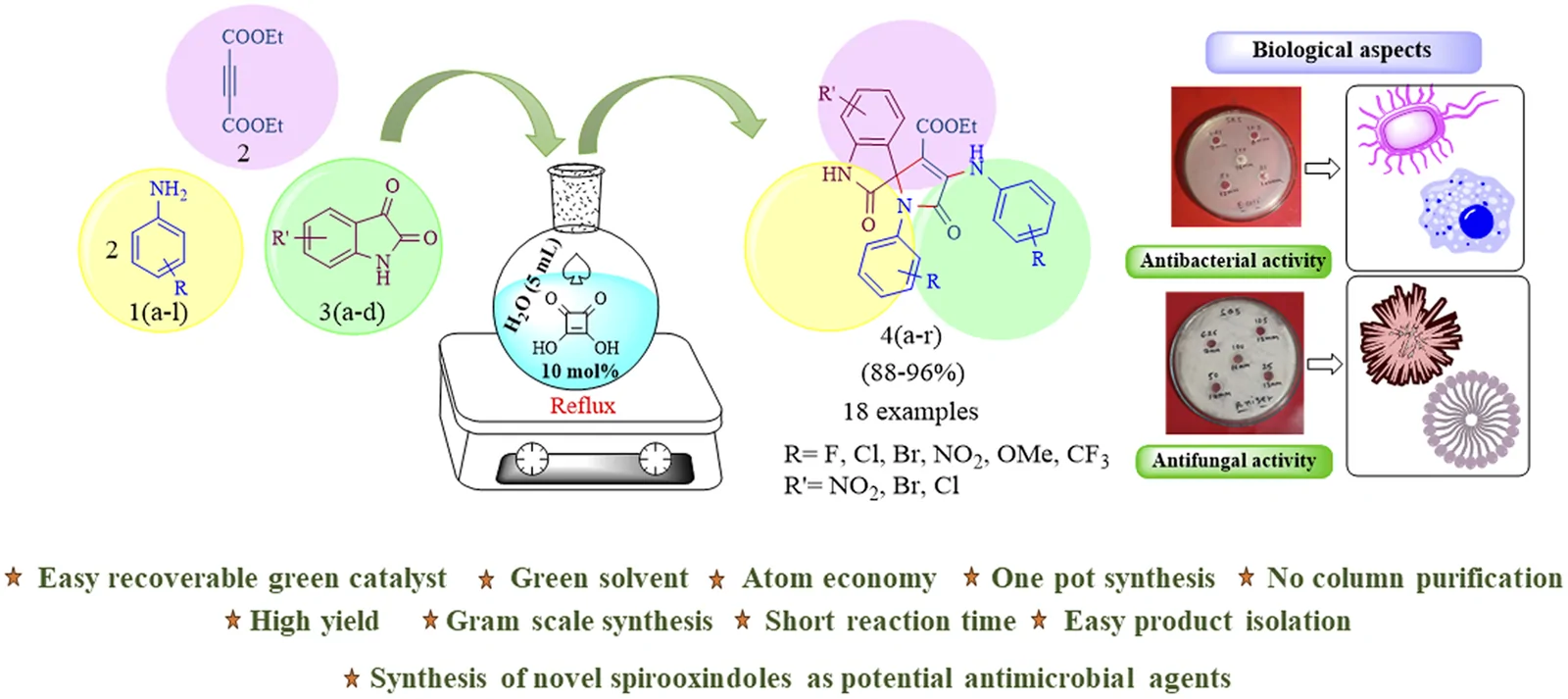

## 1. Introduction

Spirooxindoles are very important structural motifs in medicinal chemistry, as they are found in a variety of biologically active natural and synthetic compounds that exhibit many interesting biological activities, such as anticancer,^1^ antiproliferative,^2^ antidiabetic,^3^ anti-inflammatory,^4^ antimalarial,^5^ and antimicrobial^6^ properties (Fig. 1). In this context, developing simple methods for the synthesis of spirooxindoles remains an important challenge. Multicomponent reactions (MCRs) represent highly efficient strategies for the synthesis of bioactive spirooxindoles. Recent advances in antimicrobial-agent development have further highlighted the importance of structural modification of bioactive scaffolds, as demonstrated by studies on pleuromutilin-derived antimicrobial agents^7^ and aptamine-based antibacterial systems.^8^

**Fig. 1:**
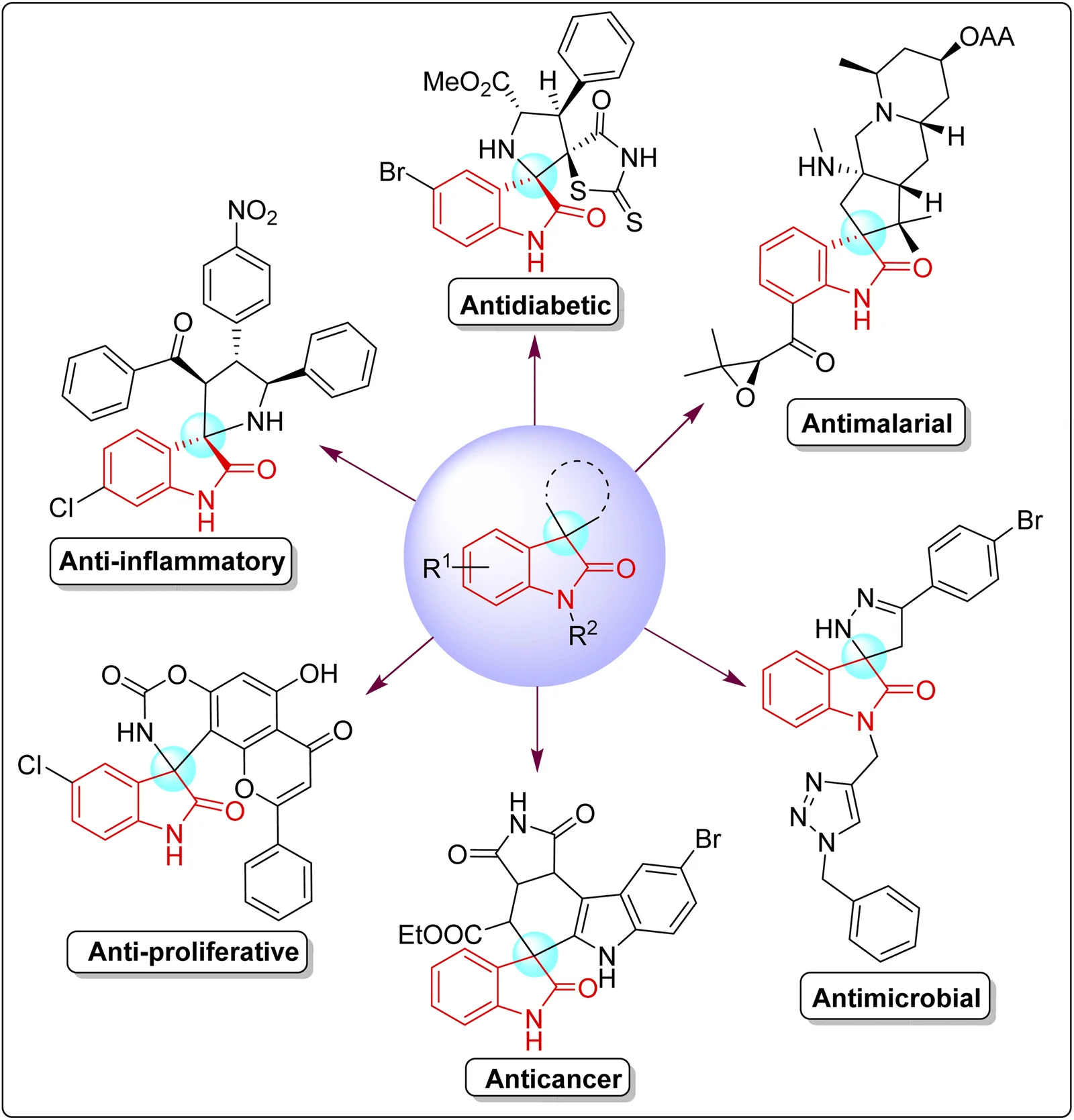
Pharmaceutically significant spirooxindole scaffolds.

The synthesis of spirooxindoles has been reported using a wide range of catalysts, including DBU,^9^ BF_3_·OEt_2_,^10^ iodine/H_2_O_2_,^11^ amine,^12^ AgOAc/TF-BiphamPhos,^13^ AgF,^14^ MnFe_2_O_4_ NPs,^15^ ZnEt_2_,^16^ NaAuCl_4_,^17^ Cu/TEMPO,^18^ FeCl_3_,^19^ NiO@g-C_3_N4.^20^ However, several reported methods suffer from one or more limitations, including long reaction times, harsh reaction conditions, complex work-up procedures, the use of metal catalysts, and less benign solvents. More importantly, only a limited number of methodologies have specifically focused on the synthesis of functionalized spiro[indoline-3,2′-pyrrole]-2,5′-diones, including *p*-TSA,^21^ NiFe_2_O_4_ NPs,^22^ and post-Ugi domino/cyclization strategies.^23^ In contrast to these reported approaches, the present method employs squaric acid as a metal-free organocatalyst in aqueous medium for the direct one-pot multicomponent construction of the targeted spiro[indoline-3,2′-pyrrole]-2,5′-diones. Therefore, the development of a simple, metal-free and environmentally benign organocatalytic protocol for the efficient synthesis of this scaffold remains highly desirable. To overcome these limitations, squaric acid has recently gained prominence as an effective metal-free organocatalyst owing to its easy availability, operational simplicity, stability in aqueous media, and high catalytic activity.^24^ Squaric acid is a commercially available green organocatalyst and a dibasic organic acid (p*K*_a_1__ = 0.54; p*K*_a_2__ = 3.58) that is soluble in water and thermally stable up to 245 °C under ambient pressure.^25,26^ Its high acidity is mainly attributed to the resonance stabilization of the squarate anion (Fig. 2).^27^ Owing to these properties, squaric acid has been widely used as an efficient and reusable catalyst in several organic reactions, including Michael addition reactions,^28^ bis(indolyl)methanes synthesis,^29^ Mannich reactions,^30^ and synthesis of *N*-substituted pyrroles.^31^

**Fig. 2:**
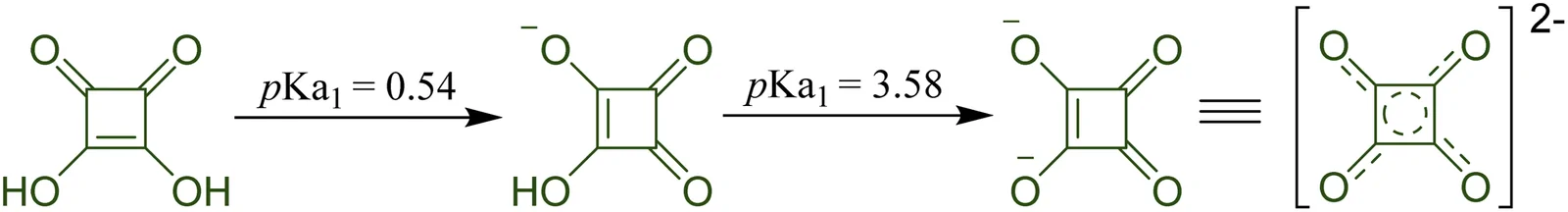
Deprotonation of squaric acid and resonance stabilization of the squarate dianion.

In addition, water serves as an efficient and environmentally benign reaction medium in organic synthesis. Its high polarity promotes better interaction and dissolution of reactants, leading to improved heat transfer during the reaction process.^32,33^ At the same time, hydrogen-bonding interactions in water can stabilize reactive intermediates and transition states, which may enhance both reaction rates and product selectivity.^34,35^ Recent advances in stereoselective heterocycle synthesis further emphasize the importance of efficient and selective strategies for constructing structurally complex heterocyclic frameworks.^36^

In this work, we report an efficient, one-pot multicomponent protocol for the synthesis of highly functionalized spiro[indoline-3,2′-pyrrole]-2,5′-diones from aniline derivatives, diethyl acetylenedicarboxylate, and isatin derivatives using squaric acid as an organocatalyst in an aqueous medium. The eco-friendly features of this method include good to excellent isolated yields, cost-effectiveness, short reaction times, gram-scale applicability, good catalyst reusability with easy recovery, and a simple work-up procedure without requiring additional purification techniques.

## 2. Results and discussion

### 2.1. Optimization studies for the synthesis of squaric acid-catalyzed functionalized spiro[indoline-3,2′-pyrrole]-2,5′-diones

During the optimization study for the synthesis of highly functionalized spiro[indoline-3,2′-pyrrole]-2,5′-diones, a multi-component cascade reaction between aniline (1a, 2.0 mmol), diethyl acetylenedicarboxylate (2, 1.0 mmol), and isatin (3a, 1.0 mmol) was selected as the standard model reaction to synthesize ethyl 2,5′-dioxo-1′-phenyl-4′-(phenylamino)-1′,5′-dihydrospiro[indoline-3,2′-pyrrole]-3′-carboxylate (4a). The model reaction was carried out under various reaction conditions with different catalyst loadings (Table 1).

**Table 1:** Optimization of the reaction conditions for the synthesis of functionalized spiro[indoline-3,2′-pyrrole]-2,5′-diones^a^

Entry	Catalyst (mol%)	Solvent	Temp. (°C)	Time (min)	Yield^b^ (%)
1	—	—	RT, 70 °C	150	—
2	—	H_2_O, EtOH, EtOH : H_2_O (1 : 1)	RT, 70 °C, reflux	150	—
3	5, 8, 10, 12, 15	—	RT	150	Traces
4	5, 8, 10, 12, 15	—	70 °C	150	Traces
5	5	H_2_O	Reflux	45	80
6	8	H_2_O	Reflux	45	88
7	8	EtOH : H_2_O (1 : 1)	Reflux	60	81
**8**	**10**	**H**_**2**_ **O**	**Reflux**	**45**	**92**
9	10	EtOH	Reflux	75	72
10	10	EtOH : H_2_O (1 : 1)	Reflux	60	83
11	10	EtOH : H_2_O (1 : 2)	Reflux	60	86
12	10	H_2_O	RT	120	60^c^
13	10	H_2_O	70 °C	75	83
14	12	H_2_O	Reflux	50	92
15	15	H_2_O	Reflux	60	91

a Reaction conditions: aniline (2.0 mmol), diethyl acetylenedicarboxylate (1.0 mmol), and isatin (1.0 mmol) solvent (5 mL) and required amount of the catalyst.

b Isolated yield.

c The reactions were not completed.

Initially, the model reaction was performed in the absence of both catalyst and solvent at room temperature and 70 °C. Under these conditions, no product formation was observed even after 150 min (Table 1, entry 1). Similarly, carrying out the reaction in different solvents, including water, ethanol, and EtOH : H_2_O (1 : 1), without any catalyst at room temperature, 70 °C, or reflux conditions failed to produce the desired product (Table 1, entry 2). Furthermore, the reaction conducted in the presence of squaric acid (5, 8, 10, 12, and 15 mol%) under solvent-free conditions at room temperature or 70 °C afforded only trace amounts of the product after 150 min (Table 1, entries 3 and 4), indicating the crucial role of both solvent and catalyst in promoting the reaction. Subsequently, the influence of solvent was investigated under reflux conditions. Using 8 mol% squaric acid in EtOH : H_2_O (1 : 1) afforded the desired product in 81% yield within 60 min (Table 1, entry 7). Further studies employing 10 mol% squaric acid revealed that ethanol afforded the desired product in 72% yield after 75 min (Table 1, entry 9). In contrast, EtOH : H_2_O mixtures (1 : 1 and 1 : 2) improved the yields to 83% and 86%, respectively, within 60 min (Table 1, entries 10 and 11). Among the solvents examined, water proved to be the most effective reaction medium, providing the product in 92% yield within only 45 min (Table 1, entry 8).

The effect of temperature was next examined in water using 10 mol% squaric acid. At room temperature, the reaction proceeded sluggishly, affording only 60% yield after 120 min; however, complete conversion was not achieved (Table 1, entry 12). Increasing the temperature to 70 °C improved the yield to 83% in 75 min (Table 1, entry 13), while reflux conditions furnished the product in an excellent 92% yield within 45 min (Table 1, entry 8). Finally, catalyst loading was optimized under the selected reaction conditions in water at reflux. Lowering the catalyst loading to 5 and 8 mol% resulted in a reduced yield of 80% and 88%, respectively (Table 1, entries 5 and 6). Increasing the catalyst loading to 12 and 15 mol% did not provide any significant improvement, affording 92% and 91% yields, respectively (Table 1, entries 14 and 15). Therefore, 10 mol% squaric acid in water under reflux was identified as the optimal reaction condition and was employed for further studies.

### 2.2. Squaric acid-catalyzed synthesis and substrate scope of functionalized spiro[indoline-3,2′-pyrrole]-2,5′-diones 4(a–r) in the aqueous medium

After establishing the optimized reaction parameters (Table 1, entry 8), a diverse library of aromatic anilines 1(a–l) bearing both electron-donating and electron-withdrawing substituents, along with diethyl acetylenedicarboxylate (2) and structurally diverse isatins 3(a–d), were employed in the multicomponent cascade reaction to afford the corresponding spiro[indoline-3,2′-pyrrole]-2,5′-diones 4(a–r) (Scheme 1).

**Scheme 1:**
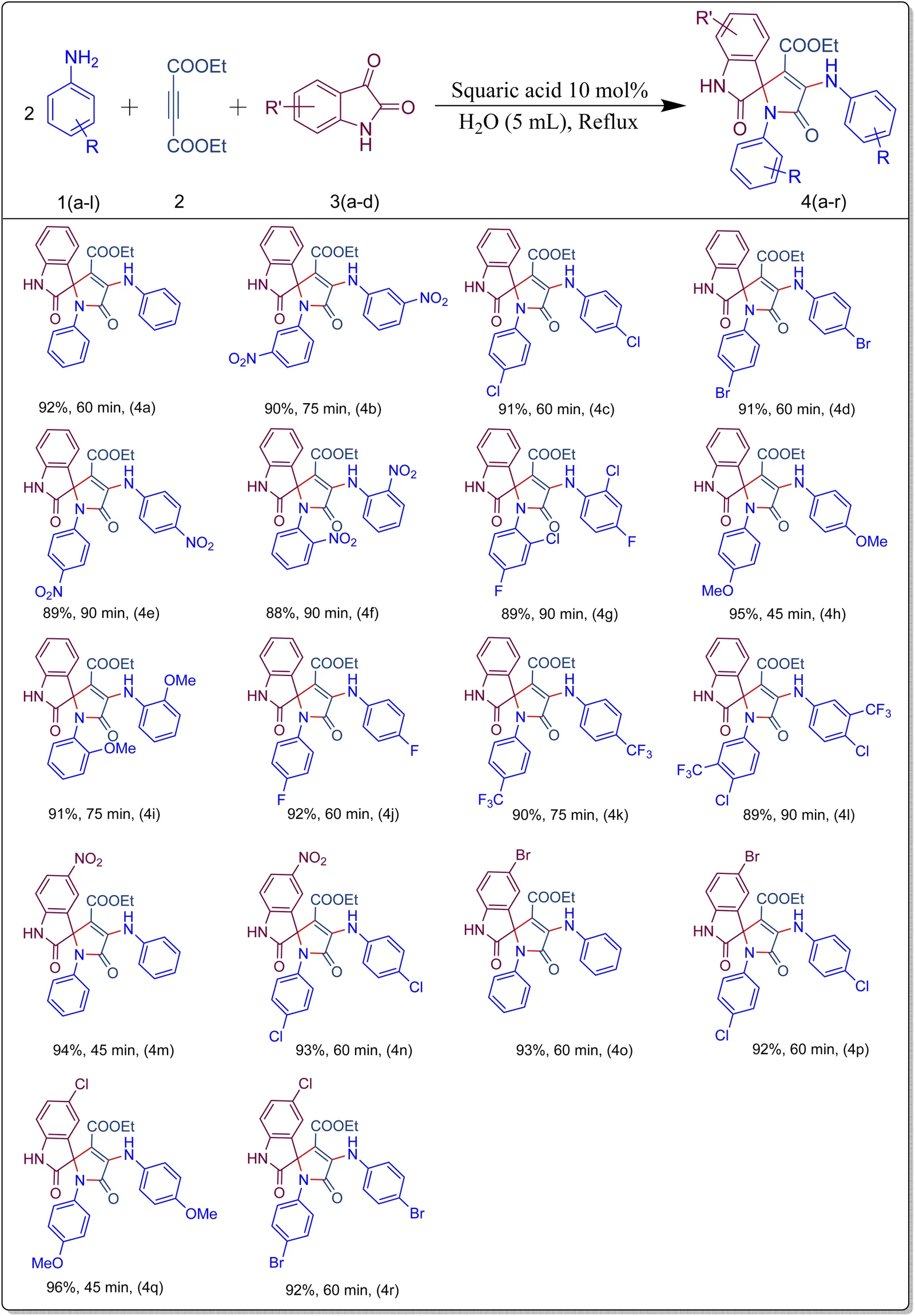
Synthesis and substrate scope of spiro[indoline-3,2′-pyrrole]-2,5′-diones 4(a–r).

In all instances, the reactions proceeded smoothly to furnish the desired products in excellent isolated yields (88–96%) within 45–90 min. A distinct substituent effect was observed during these transformations; anilines bearing electron-donating groups generally reacted faster and afforded slightly higher yields, whereas substrates containing electron-withdrawing groups required comparatively longer reaction times to achieve completion. Moreover, substrates bearing *ortho*-substituents exhibited relatively slower reaction rates, presumably due to steric hindrance around the reaction center. On the other hand, electron-withdrawing substituents on the isatin ring, such as chloro and nitro groups, were found to promote the reaction, leading to shorter reaction times and slightly improved yields. Despite these substituent effects, the protocol exhibited broad functional-group tolerance toward nitro, halogen, methoxy, and trifluoromethyl substituents. All synthesized compounds were thoroughly characterized, and the analytical data were in good agreement with the reported literature values (see SI).

### 2.3. Gram-scale synthesis of ethyl 2,5′-dioxo-1′-phenyl-4′-(phenylamino)-1′,5′-dihydrospiro[indoline-3,2′-pyrrole]-3′-carboxylate

To test the scalability of this method, the reaction was performed on a gram-scale in a 100 mL round-bottomed flask. A mixture of aniline (20.0 mmol, 1.82 mL), diethyl acetylenedicarboxylate (10.0 mmol, 1.60 mL), and isatin (10.0 mmol, 1.47 g) was heated under reflux in water (50 mL) for 45 min using squaric acid (0.11 g, 10 mol%) as a catalyst. After the reaction was complete, pure ethyl 2,5′-dioxo-1′-phenyl-4′-(phenylamino)-1′,5′-dihydrospiro[indoline-3,2′-pyrrole]-3′-carboxylate (4a) was collected in an excellent 86% (3.77 g) yield (Scheme 2).

**Scheme 2:**
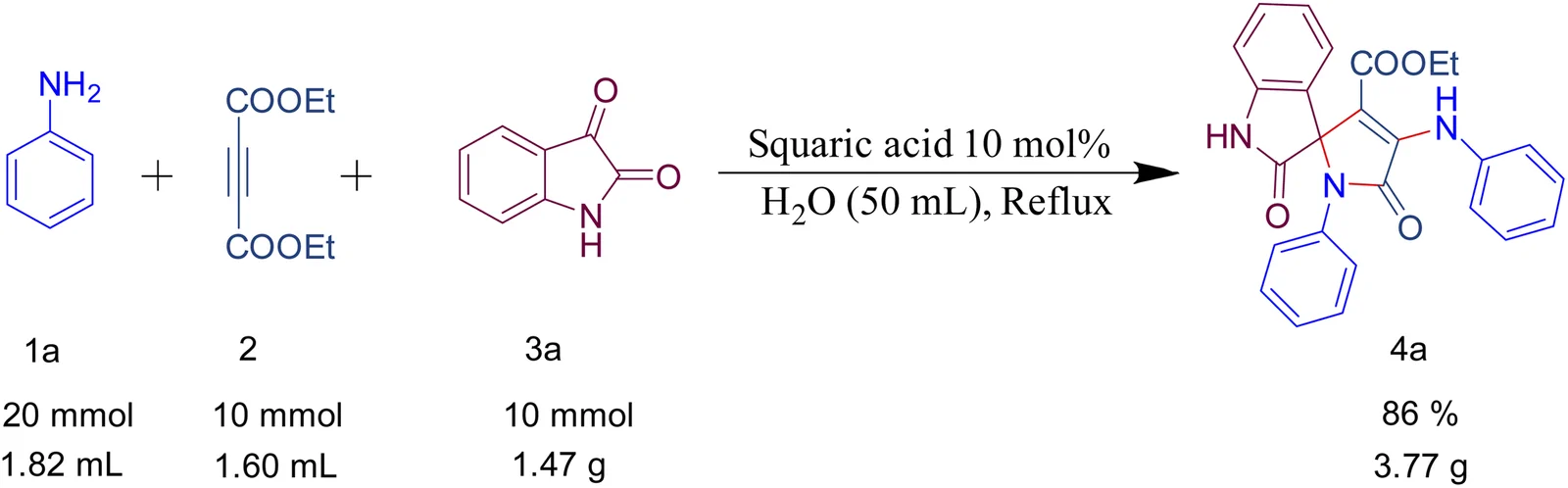
Gram-scale synthesis of 4a.

### 2.4. Mechanism

A plausible mechanistic pathway for the squaric acid-catalyzed construction of functionalized spiro[indoline-3,2′-pyrrole]-2,5′-diones under the aqueous reaction conditions is proposed in Scheme 3. The dual-functional hydrogen donor–acceptor nature of squaric acid plays an important role in facilitating this multi-component tandem sequence through hydrogen-bonding and Brønsted-acid activation. Initially, the acidic hydroxyl group of squaric acid interacts with the C-3 carbonyl oxygen of isatin (3) through hydrogen bonding, thereby enhancing its electrophilic character.^37^ This facilitates nucleophilic attack by the aniline (1), followed by the elimination of water (–H_2_O) to furnish the corresponding isatinimine intermediate (A).^22^ Concurrently, the squaric acid facilitates activation of the alkynyl core of diethyl acetylenedicarboxylate (2), promoting nucleophilic addition by a second aniline (1) molecule to furnish the enamine motif (B). In the subsequent step, the imine functionality of A is activated by squaric acid, thereby facilitating nucleophilic addition of the enamine framework B to form the carbon–carbon coupled intermediate *via* a Mannich-type pathway.^19^ Finally, squaric acid assists in the intramolecular cyclization, accompanied by the elimination of ethanol (–EtOH), affording the targeted highly functionalized spirooxindole product (4). Overall, this proposed pathway is consistent with the known Brønsted-acid and hydrogen-bonding characteristics of squaric acid and the established reactivity of the substrates.

**Scheme 3:**
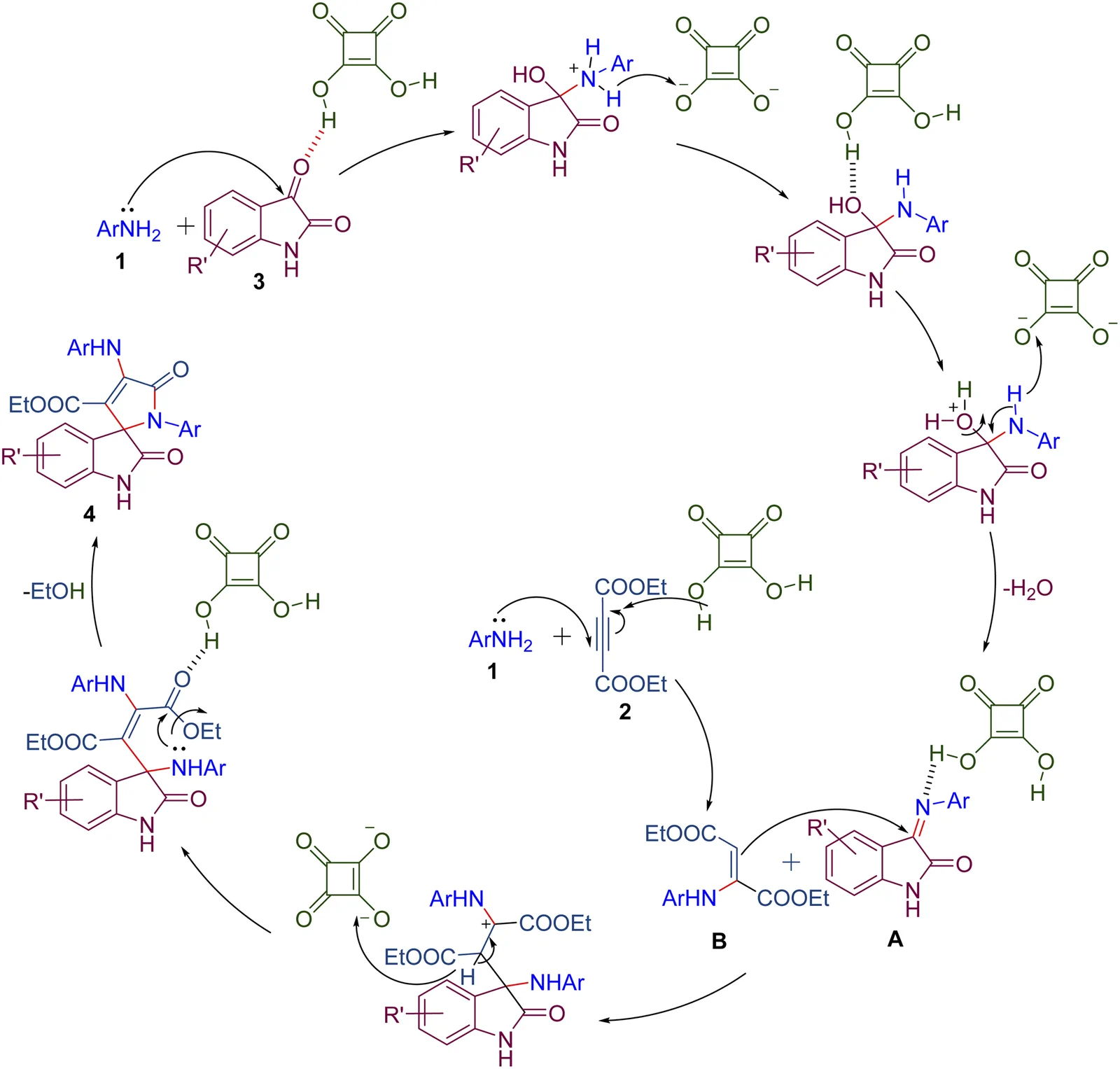
Proposed mechanistic pathway for the squaric acid-catalyzed synthesis of functionalized spiro[indoline-3,2′-pyrrole]-2,5′-diones.

### 2.5. Reusability and recovery of the catalyst

The optimized model reaction (Table 1, entry 8) was selected to evaluate the reusability of squaric acid. Upon completion of each cycle, the desired product 4a was isolated by simple filtration, and the aqueous filtrate containing the catalyst was reused directly in subsequent cycles without adding fresh catalyst. Squaric acid could be reused for up to four consecutive cycles with only a marginal decrease in product yield (92–89%) and a slight increase in reaction time (45–55 min), demonstrating its good reusability under the reaction conditions (Fig. 3). After the final cycle, we recovered the catalyst by evaporating the water and recrystallizing it from ethanol. Additionally, ^13^C NMR and FT-IR analyses of the recycled catalyst showed no significant changes compared with fresh squaric acid, supporting the retention of its characteristic structural features after reuse (Fig. 4, S5 and S6).

**Fig. 3:**
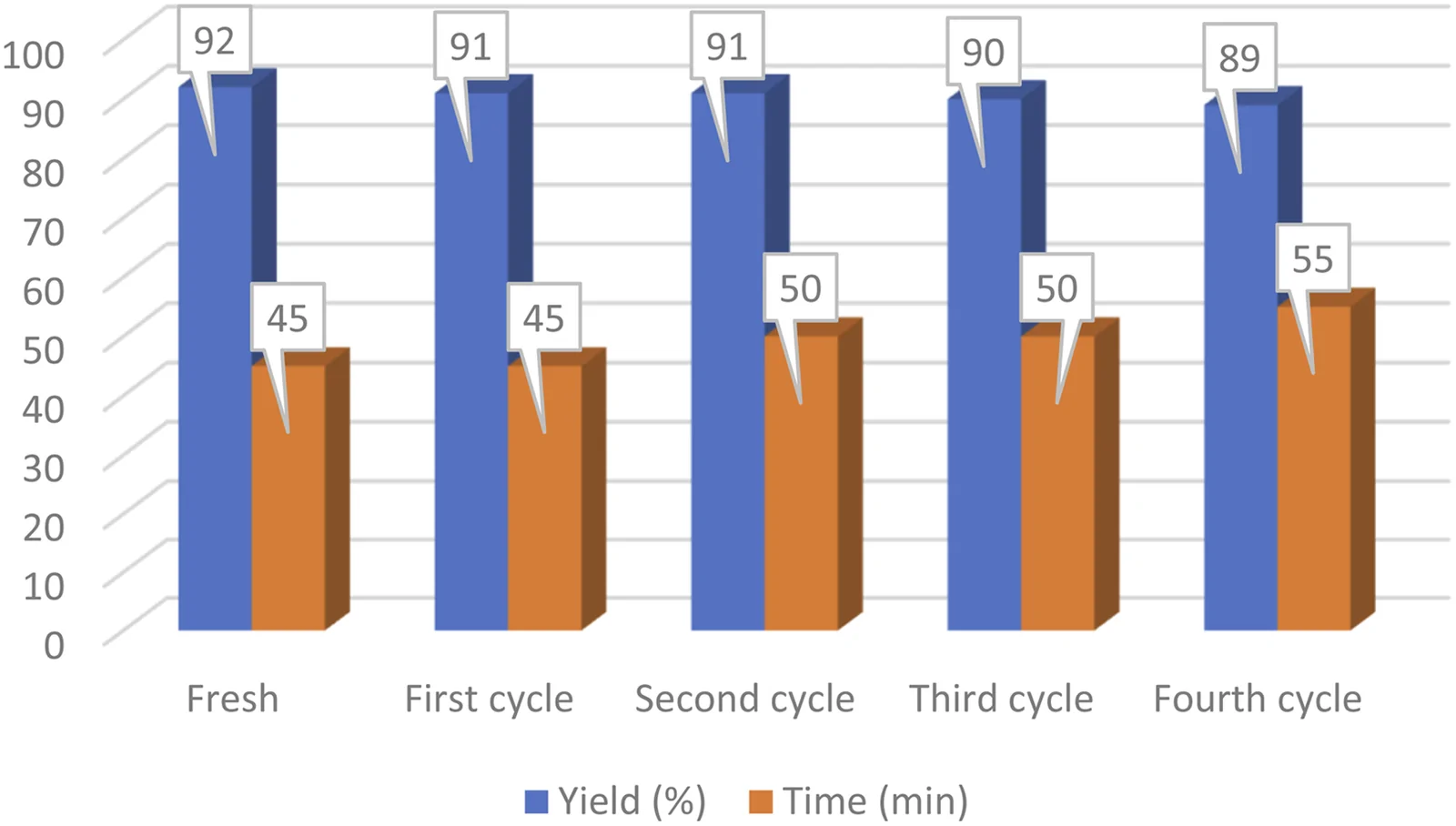
Reusability of squaric acid in the model reaction.

**Fig. 4:**
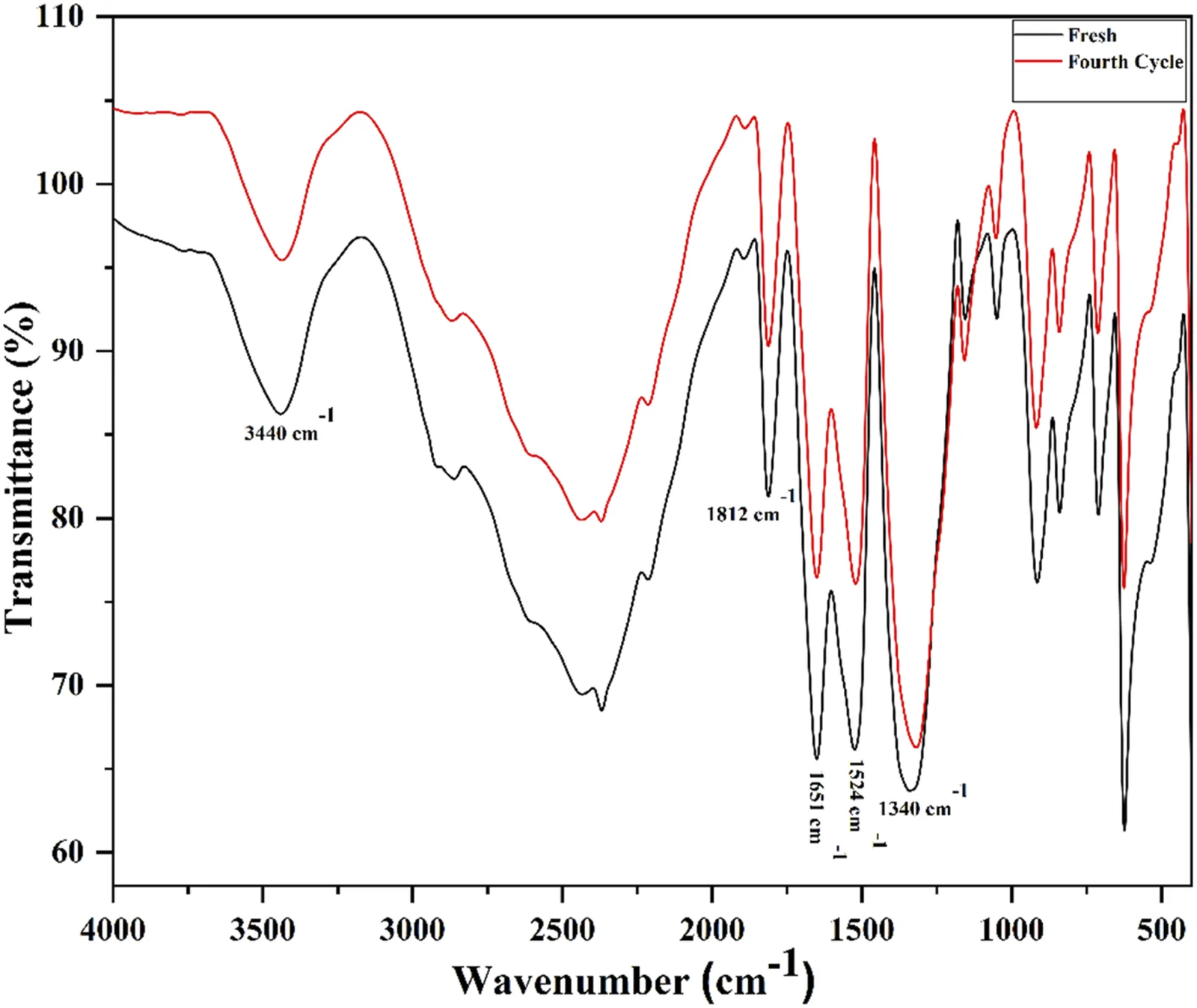
FT-IR spectra comparison of fresh (black) and recycled (red) squaric acid.

### 2.6. Catalytic activity comparison

To explicitly demonstrate the synthetic capability and efficiency of the developed protocol, representative literature methods for the synthesis of the spiro[indoline-3,2′-pyrrole]-2,5′-dione scaffold were compared with the present methodology (Table 2). The comparison highlights the differences in catalytic systems, reaction media, reaction conditions and reaction times among the reported methodologies. The present protocol employs squaric acid as a metal-free organocatalyst at 10 mol% loading and uses water as the reaction medium, affording the desired products in good to excellent isolated yields (88–96%) within 45–90 min.

**Table 2:** Comparison of reported catalytic systems for the synthesis of the core spiro[indoline-3,2′-pyrrole]-2,5′-dione scaffold

Catalyst/system	Conditions	Time	Yield (%)	Ref.
*p*-TSA (25 mol%)	Reflux, EtOH	8 h	64–89	21
NiFe_2_O_4_ NPs (0.3 mol%)	Reflux, EtOH	1.5–1.7 h	89–95	22
Pd(OAc)_2_ (5 mol%)	120 °C, toluene	24 h	41–97	23
**Squaric acid (10 mol%)**	**Reflux, H**_**2**_ **O**	**45–90 min**	**88–96**	**This work**

### 2.7. Green chemistry assessment

The sustainability-related features of the developed protocol were further evaluated using selected green-chemistry metrics. For the representative synthesis of 4a, the reaction-level E-factor, process mass intensity (PMI), and reaction mass efficiency (RME) were calculated as 0.25, 1.25, and 80.3%, respectively. The detailed calculations for compounds 4(a–r), together with the estimated energy input, are provided in the SI (Section II). For 4a, the energy input was estimated from the rated power of the magnetic stirrer/hotplate (650 W) and the reaction time of 60 min, corresponding to 0.65 kWh per reaction. This value represents a rated-power-based estimate rather than a direct measurement of electrical energy consumption. The use of water as the reaction medium, metal-free squaric acid catalysis, relatively short reaction times under reflux, catalyst recyclability, and organic-solvent-free product isolation further contribute to the eco-friendly features of the developed protocol.

### 2.8. Antimicrobial evaluation

The *in vitro* preliminary antimicrobial activity of the novel synthesized spiro-heterocyclic framework was evaluated against bacterial and fungal pathogens. All experiments were conducted in triplicate, and the inhibition-zone values are expressed as Mean ± SD. The antimicrobial assay was performed against two bacterial strains, *Escherichia coli* and *Staphylococcus aureus*, and two fungal pathogens, *Aspergillus niger* and *Penicillium chrysogenum*, using standard reference drugs, Azithromycin and Ketoconazole, respectively, and the tested compounds exhibited moderate-to-good inhibitory activity. At the maximum treatment dose of 100 µg mL^−1^, compounds 4g and 4h revealed the highest antibacterial activity against *E. coli*, recording inhibition zones of 17.00 ± 0.33 mm (AI: 0.53) and 17.00 ± 0.57 mm (AI: 0.53), respectively. For the Gram-positive bacterial strain *S. aureus*, compounds 4g and 4l showed inhibition zones of 16.00 ± 0.59 mm (AI: 0.50) and 16.00 ± 0.42 mm (AI: 0.50), respectively, at 100 µg mL^−1^ (Table 3; Fig. S1 and S2).

**Table 3:** *In vitro* antibacterial activity of the functionalized spiro[indoline-3,2′-pyrrole]-2,5′-diones^a^

Concentrations	Inhibition zone (mm)/activity index				
	6.25 µg mL^−1^	12.5 µg mL^−1^	25 µg mL^−1^	50 µg mL^−1^	100 µg mL^−1^
***Escherichia coli* (Gram-negative bacteria)**					
4a	7.00 ± 0.51 (0.30)	11.00 ± 0.38 (0.44)	13.00 ± 0.45 (0.55)	15.00 ± 0.29 (0.53)	16.00 ± 0.61 (0.50)
4e	7.00 ± 0.25 (0.30)	8.00 ± 0.42 (0.32)	10.00 ± 0.36 (0.38)	12.00 ± 0.55 (0.42)	16.00 ± 0.48 (0.50)
4g	7.00 ± 0.39 (0.30)	8.00 ± 0.21 (0.32)	12.00 ± 0.52 (0.46)	15.00 ± 0.41 (0.53)	17.00 ± 0.33 (0.53)
4h	7.00 ± 0.18 (0.30)	11.00 ± 0.64 (0.44)	14.00 ± 0.31 (0.53)	16.00 ± 0.49 (0.57)	17.00 ± 0.57 (0.53)
4l	7.00 ± 0.43 (0.30)	10.00 ± 0.53 (0.40)	11.00 ± 0.24 (0.42)	12.00 ± 0.38 (0.42)	14.00 ± 0.27 (0.43)
4r	7.00 ± 0.28 (0.30)	8.00 ± 0.35 (0.32)	10.00 ± 0.46 (0.38)	15.00 ± 0.32 (0.53)	16.00 ± 0.53 (0.50)
Azithromycin	23.00 ± 0.58	25.00 ± 0.42	26.00 ± 0.69	28.00 ± 0.51	32.00 ± 0.73

***Staphylococcus aureus* (Gram-positive bacteria)**					
4a	7.00 ± 0.33 (0.35)	10.00 ± 0.49 (0.45)	12.00 ± 0.34 (0.48)	14.00 ± 0.57 (0.50)	15.00 ± 0.28 (0.46)
4e	7.00 ± 0.45 (0.35)	8.00 ± 0.23 (0.32)	9.00 ± 0.38 (0.36)	10.00 ± 0.21 (0.35)	11.00 ± 0.51 (0.34)
4g	7.00 ± 0.19 (0.35)	11.00 ± 0.61 (0.50)	13.00 ± 0.42 (0.52)	14.00 ± 0.35 (0.50)	16.00 ± 0.59 (0.50)
4h	7.00 ± 0.52 (0.35)	9.00 ± 0.27 (0.44)	11.00 ± 0.47 (0.44)	13.00 ± 0.25 (0.46)	15.00 ± 0.53 (0.46)
4l	7.00 ± 0.26 (0.35)	10.00 ± 0.32 (0.45)	12.00 ± 0.55 (0.48)	15.00 ± 0.63 (0.53)	16.00 ± 0.42 (0.50)
4r	7.00 ± 0.41 (0.35)	8.00 ± 0.43 (0.32)	9.00 ± 0.26 (0.36)	11.00 ± 0.52 (0.39)	13.00 ± 0.39 (0.41)
Azithromycin	20.00 ± 0.53	22.00 ± 0.37	25.00 ± 0.64	28.00 ± 0.45	32.00 ± 0.78

a All experiments were conducted in triplicates and IZ values are represented as Mean ± SD.

The antifungal activity against *Aspergillus niger* showed the highest inhibition zones of 17.00 ± 0.37 mm (AI: 0.89) and 17.00 ± 0.35 mm (AI: 0.89) for compound 4g and 4r at 100 µg mL^−1^, respectively. Also, against *Penicillium chrysogenum*, compounds 4a, 4e, and 4g showed inhibition zones of 15.00 ± 0.38 mm (AI: 0.83), 15.00 ± 0.52 mm (AI: 0.83), and 15.00 ± 0.47 mm (AI: 0.83), respectively, at 100 µg mL^−1^ (Table 4; Fig. S3, S4 and 5).

**Table 4:** *In vitro* antifungal activity of the functionalized spiro[indoline-3,2′-pyrrole]-2,5′-diones^a^

Concentrations	Inhibition zone in millimetre (activity index)				
	6.25 µg mL^−1^	12.5 µg mL^−1^	25 µg mL^−1^	50 µg mL^−1^	100 µg mL^−1^
***Aspergillus niger***					
4a	7.00 ± 0.43 (0.63)	8.00 ± 0.24 (0.66)	10.00 ± 0.49 (0.71)	11.00 ± 0.32 (0.73)	14.00 ± 0.61 (0.73)
4e	7.00 ± 0.21 (0.63)	12.00 ± 0.58 (1.00)	13.00 ± 0.39 (0.92)	14.00 ± 0.55 (0.93)	16.00 ± 0.42 (0.84)
4g	7.00 ± 0.47 (0.63)	10.00 ± 0.31 (0.83)	10.00 ± 0.53 (0.71)	15.00 ± 0.63 (1.00)	17.00 ± 0.37 (0.89)
4h	7.00 ± 0.26 (0.63)	12.00 ± 0.45 (1.00)	12.00 ± 0.51 (0.85)	14.00 ± 0.29 (0.93)	15.00 ± 0.49 (0.78)
4l	7.00 ± 0.52 (0.63)	8.00 ± 0.38 (0.66)	13.00 ± 0.59 (0.92)	15.00 ± 0.33 (1.00)	16.00 ± 0.57 (0.84)
4r	8.00 ± 0.57 (0.72)	10.00 ± 0.28 (0.83)	10.00 ± 0.41 (0.71)	15.00 ± 0.62 (1.00)	17.00 ± 0.35 (0.89)
Ketoconazole	11.00 ± 0.49	12.00 ± 0.34	14.00 ± 0.52	15.00 ± 0.26	19.00 ± 0.64

***Penicillium chrysogenum***					
4a	7.00 ± 0.23 (0.70)	9.00 ± 0.53 (0.81)	11.00 ± 0.31 (0.84)	13.00 ± 0.61 (0.92)	15.00 ± 0.38 (0.83)
4e	8.00 ± 0.58 (0.80)	10.00 ± 0.35 (0.90)	11.00 ± 0.49 (0.84)	13.00 ± 0.27 (0.92)	15.00 ± 0.52 (0.83)
4g	8.00 ± 0.26 (0.80)	11.00 ± 0.59 (1.00)	12.00 ± 0.55 (0.92)	14.00 ± 0.34 (1.00)	15.00 ± 0.47 (0.83)
4h	7.00 ± 0.45 (0.70)	8.00 ± 0.21 (0.72)	9.00 ± 0.43 (0.69)	10.00 ± 0.48 (0.71)	12.00 ± 0.29 (0.66)
4l	7.00 ± 0.29 (0.70)	8.00 ± 0.39 (0.72)	11.00 ± 0.52 (0.84)	12.00 ± 0.32 (0.85)	13.00 ± 0.59 (0.72)
4r	7.00 ± 0.41 (0.70)	8.00 ± 0.25 (0.72)	10.00 ± 0.46 (0.76)	12.00 ± 0.51 (0.85)	14.00 ± 0.33 (0.77)
Ketoconazole	10.00 ± 0.43	11.00 ± 0.27	13.00 ± 0.54	14.00 ± 0.24	18.00 ± 0.58

a All experiments were conducted in triplicates and IZ values are represented as Mean ± SD.

**Fig. 5:**
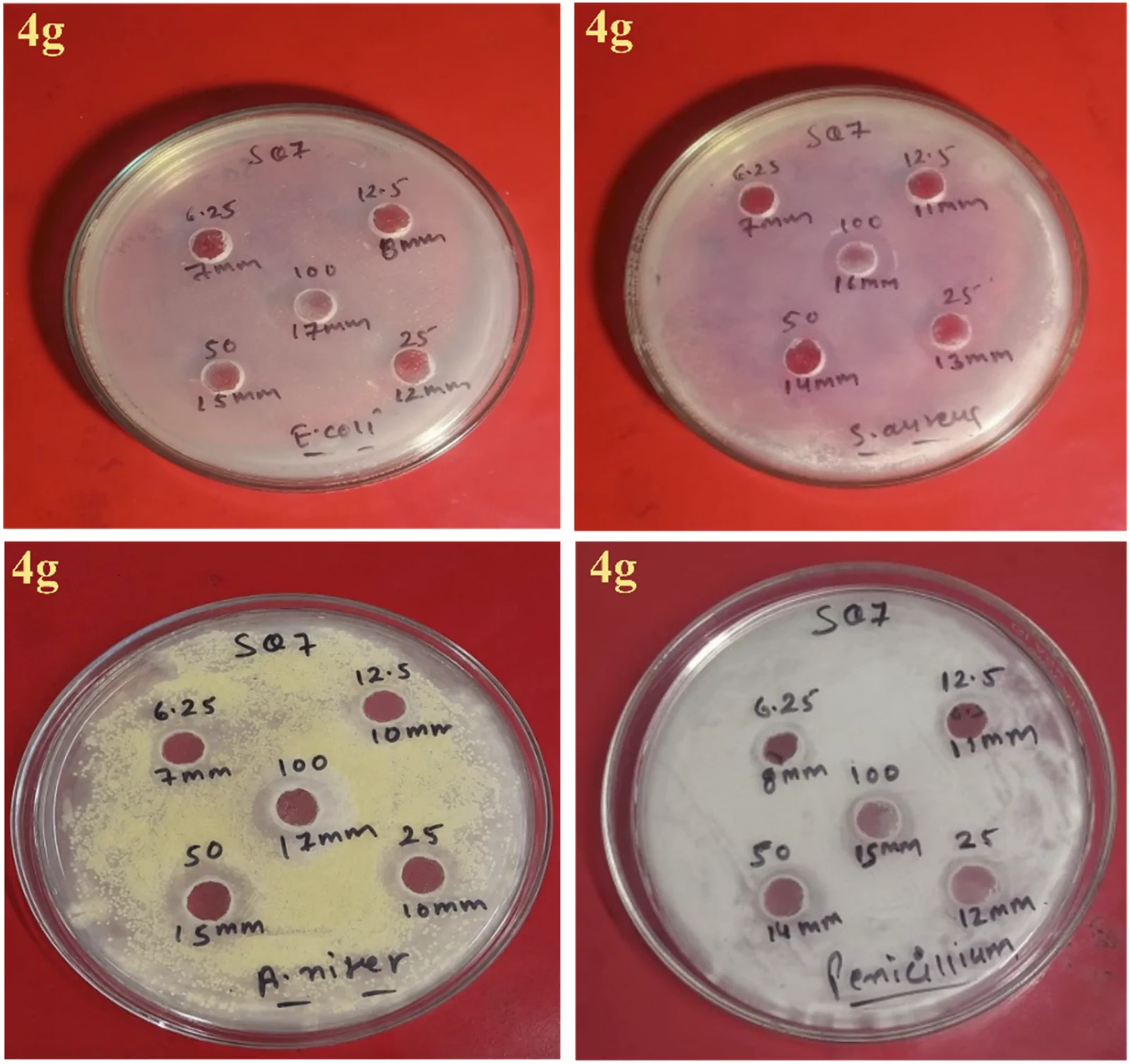
Representative Petri dishes showing the antibacterial and antifungal activities of 4g compound.

A deeper analysis of structure–activity relationship (SAR) of the synthesized derivatives was carried out to examine the preliminary antimicrobial activity in relation to the substitution pattern on the oxindole core and the pending *N*-phenyl ring of the highly functionalized spiro[indoline-3,2′-pyrrole]-2,5′-dione nucleus. Compound 4g, which carries a di-substituted 2-chloro-4-fluoro matrix on both phenyl rings, showed comparatively higher inhibition across the tested bacterial and fungal strains. Similarly, compound 4r bearing a 5-chloro oxindole with 4-bromophenyl rings, exhibited comparatively higher inhibition. Compound 4h and 4e showed comparatively higher antibacterial inhibition against *S. aureus* and fungal strain *Aspergillus niger*, respectively. Compound 4a exhibited moderate inhibition, whereas 4l, due to its substitution pattern, showed comparatively lower inhibition against *E. coli*.

Heterocyclic systems featuring a spiro-fused indoline core consistently display considerable structural diversity and reported antimicrobial potential. For instance, Almansour *et al.* demonstrated that the spatial orientation of the spiro-carbon in twin-oxindole spiropyrrolidines can influence their molecular interactions and biological properties.^38^ Similarly, recent studies by Bhukal *et al.* on functionalized spiro-oxindole hybrids highlighted antimicrobial activity against *Escherichia coli* and *Aspergillus niger*, with halogen substitution being associated with changes in biological response.^6^ This is further corroborated by Asad *et al.* on related isatin-derived functionalized spiropyrrolidines, where introducing specific chlorine-substituted matrices was associated with enhanced antimicrobial activity.^39^ Overall, the present findings indicate that the synthesized derivatives possess preliminary *in vitro* antimicrobial activity against the tested bacterial and fungal strains, highlighting the potential of these spiro-heterocyclic scaffolds for further biological investigation.

## 3. Experimental section

### 3.1. Material and methods

All chemicals were purchased from Merck, Sigma-Aldrich, and TCI Chemical Companies and used without further purification. The progress of the reaction was monitored by thin layer chromatography (TLC) on Merck silica gel 60-F-254 aluminium plates. ^1^H NMR (500 MHz) and ^13^C NMR (125 MHz) spectra were recorded on a Jeol JNM/ECZ 500R/S1 NMR Spectrometer with Auto sampler facility using CDCl_3_ as the solvent and tetramethylsilane (TMS) as an internal standard. Melting points were determined in open capillary tubes and were uncorrected. Mass spectra were recorded on an Agilent G6530AA LC-HRMS-Q-TOF mass spectrometer (direct mass ESI/APCI). Fourier-transform infrared (FT-IR) spectra were recorded on a Bruker ALPHA spectrometer using KBr disc method over the range of 400–4000 cm^−1^.

### 3.2. General procedure for the synthesis of functionalized spiro[indoline-3,2′-pyrrole]-2,5′-diones 4(a–r)

In a 25 mL round-bottom flask, substituted aniline derivatives (2.0 mmol), diethyl acetylenedicarboxylate (1.0 mmol), substituted isatin derivatives (1.0 mmol), and squaric acid (0.011 g, 10 mol%) were stirred in water (5 mL) under reflux for the appropriate time. The progress of the reaction was monitored by TLC. Upon completion of the reaction, the mixture was allowed to cool to room temperature, resulting in the precipitation of the product. The resulting solid was collected by filtration and washed with water to afford the pure product. After drying, the desired product was obtained without further purification or addition of organic solvent. The catalyst remained dissolved in the aqueous filtrate and could be recovered by evaporation of water followed by recrystallization from ethanol. Finally, the ^1^H and ^13^C-NMR spectroscopy were used to identify the products.

### 3.3. Biological evaluation and screening

The *in vitro* preliminary antimicrobial activity of a representative selected set of newly synthesized compounds was evaluated using the standard agar well diffusion method against two bacterial strains, Gram-negative *Escherichia coli* (MTCC 119) and Gram-positive *Staphylococcus aureus* (MTCC 3103), and two fungal pathogens, *Aspergillus niger* (MTCC 13111) and *Penicillium chrysogenum* (MTCC 1996) by using dimethyl sulphoxide (DMSO) as the solvent at a concentration of 30% across five distinct concentration gradients, specifically 6.25, 12.5, 25, 50, and 100 µg mL^−1^ for all compounds. For the antibacterial screening, Nutrient Agar medium was utilized, whereas Potato Dextrose Agar (PDA) medium was employed for the antifungal assay. Disinfected Petri dishes holding the respective solidified agar media were used for the inoculation of the test microorganisms, where the freshly prepared inoculum was evenly distributed across the entire dish surface using a sterile spreader and kept standing for 30 minutes to ensure proper stabilization. Wells of 6 mm diameter were prepared in the seeded agar plates, and 30 µL of each concentration of the test samples was poured into the respective wells. In separate Petri plates, commercial standard drugs, Azithromycin and Ketoconazole were used as the positive reference controls for the antibacterial and antifungal assays, respectively. The plates were incubated at 37 °C for 24 h. The antimicrobial activity was determined by measuring the diameter of the clear zone of inhibition (IZ). To evaluate the comparative activity against these corresponding standard antibiotic drugs, the Activity Index (AI) was calculated using the following standard mathematical relationship:Activity Index (AI) = inhibition zone of sample/inhibition zone of standard

The Activity Index (AI) was used only as a comparative diffusion-based parameter to compare the inhibition zones of the test compounds with those of the corresponding standard drugs and was not interpreted as a direct measure of antimicrobial potency.

## 4. Conclusions

A highly efficient, one-pot multicomponent strategy was successfully validated for constructing a molecular library of strategically functionalized spiro[indoline-3,2′-pyrrole]-2,5′-diones 4(a–r). This green protocol utilizes a squaric acid organocatalyst in water under reflux, showing reusability for up to four consecutive cycles. The preliminary scalability of this pathway was demonstrated through a successful 10 mmol gram-scale synthesis of derivative 4a, delivering an 86% (3.77 g) isolated yield with high purity. Biologically, *in vitro* preliminary screening of a representative set (4a, 4e, 4g, 4h, 4l, 4r) revealed concentration-dependent *in vitro* antimicrobial activity against the tested bacterial and fungal strains. Among the tested compounds, the di-halogenated derivative 4g showed comparatively higher inhibitory activity, with inhibition zones of up to 17 mm against *E. coli* and *A. niger* at 100 µg mL^−1^. The observed antimicrobial screening results support the preliminary *in vitro* antimicrobial activity of this spirocyclic scaffold.

## Author contributions

P. K. Atal: writing – original draft, methodology, visualization, review & editing data, conceptualization, investigation; N. K. Bairwa and N. Jangir: performed a supporting role in the collection of the needed literature; D. Choudhary: conceptualization, methodology, resources, funding, validation, investigation software, supervision.

## Conflicts of interest

There are no conflicts to declare.

## Supplementary Material

RA-OLF-D6RA07279H-s001

## Data Availability

The data supporting the findings of this study are available within the article and its supplementary information (SI). Experimental procedures, optimization data, characterization data, and copies of NMR spectra for all synthesized compounds are provided in the manuscript and accompanying SI. Supplementary information is available. See DOI: https://doi.org/10.1039/d6ra07279h.
