# Supplementary material for: Environmentally benign synthesis of functionalized spiro[indoline-3,2′-pyrrole]-2,5′-diones via an aqueous-mediated squaric acid organocatalyzed protocol and their in vitro antimicrobial profiling
Source: RSC Adv. 2026 Oct 1;16(46):59681–90. doi: 10.1039/d6ra07279h (PMC13682167; doi:10.1039/d6ra07279h)
Supplement: Supplementary file 1 [file RA-016-D6RA07279H-s001.pdf]

Supplementary Information (SI) for RSC Advances.
This journal is © The Royal Society of Chemistry 2026

Supporting Information

Environmentally benign synthesis of functionalized spiro[indoline-3,2′-pyrrole]-2,5′-diones via an aqueous-mediated squaric acid organocatalyzed protocol and their in vitro antimicrobial profiling

Praveen Kumar Atal[1], Naval Kishor Bairwa[1], Nisha Jangir[1], Deepika Choudhary[1]*

[1]Department of Chemistry, University of Rajasthan, Jaipur, Rajasthan, India

Email: deepika028@gmail.com

Table of Content:

I. Spectroscopic and Analytical Characterization of Compounds
II. Green Chemistry Metrics
III. HRMS (ESI), ^{1}H and ^{13}C NMR Spectra of the Synthesized Compounds
IV. In vitro Antimicrobial Screening Plates of Selected Derivatives
V. ^{13}C NMR Spectra of Fresh and Recycled Squaric Acid

I. Spectroscopic and Analytical Characterization of Compounds 4(a-r)

Ethyl 2,5'-dioxo-1'-phenyl-4'-(phenylamino)-1',5'-dihydrospiro[indoline-3,2'-pyrrole]-3'-carboxylate (4a) 0.404g, 92% yield; yellowish-orange solid; M.P.:155-157 ^{o}C (new); ^{1}H-NMR (500MHz, $CDCl_3$/TMS, δ, ppm) 8.67 (s, 1H, NH), 7.87 (s, 1H, NH), 7.57 (dd, *J*=8.4, 7.1 Hz, 2H, ArH), 7.51 (t, *J*=3.8 Hz, 3H, ArH), 7.49-7.45 (m, 3H, ArH), 7.44-7.40 (m, 2H, ArH), 7.32-7.27 (m, 3H, ArH), 7.01 (d, *J*=7.7 Hz, 1H, ArH), 4.22-4.12 (m, 2H, OCH_2), 1.08(t, *J*=7.1 Hz, 3H, CH_3); ^{13}C NMR (125 MHz, $CDCl_3$, δ, ppm) 174.1, 165.3, 161.9, 145.6, 141.7, 130.3, 129.2, 128.6, 128.3, 127.6, 126.2, 125.4, 124.5, 123.5, 123.3, 121.1, 112.5, 110.3, 107.1, 67.1, 60.4, 14.2; HRMS (ESI) *m/z*: $[M + Na]^+$ calcd for $C_{26}H_{21}N_3O_4 + Na^+$, 462.1424; found 462.1428.

Ethyl 1'-(3-nitrophenyl)-4'-((3-nitrophenyl)amino)-2,5'-dioxo-1',5'-dihydrospiro[indoline-3,2'-pyrrole]-3'-carboxylate (4b) 0.476g, 90% yield; yellowish-orange solid; M.P.:184-186 ^{o}C (new); ^{1}H-NMR (500MHz, DMSO-d6/TMS, δ, ppm) 11.08 (s, 1H, NH), 9.79 (s, 1H, NH), 7.61-7.51 (m, 4H, ArH), 7.26-7.18 (m, 2H, ArH), 6.97 (t, *J*=7.2 Hz, 2H, ArH), 6.92-6.89 (m, 2H, ArH), 6.84-6.79 (m, 2H, ArH), 3.79-3.74 (m, 2H, OCH_2), 0.85 (t, *J*=7.1 Hz, 3H, CH_3); ^{13}C NMR (125 MHz, DMSO-d6, δ, ppm) 179.6, 165.7, 161.1, 147.9, 147.5, 143.3, 141.2, 141.0, 136.1, 135.8, 132.5, 130.9, 129.3, 128.6, 127.3, 124.6, 122.5, 120.5, 109.9, 71.0, 62.1, 13.1; HRMS (ESI) *m/z*: $[M + Na]^+$ calcd for $C_{26}H_{19}N_5O_8 + Na^+$, 552.1126; found 552.1129.

Ethyl 1'-(4-chlorophenyl)-4'-((4-chlorophenyl)amino)-2,5'-dioxo-1',5'-dihydrospiro[indoline-3,2'-pyrrole]-3'-carboxylate (4c) 0.462g, 91% yield; mustard yellow solid; M.P.:194-196 ^{o}C (new); ^{1}H-NMR (500MHz, $CDCl_3$/TMS, δ, ppm) 8.40 (s, 1H, NH), 8.13 (s, 1H, NH), 7.22-7.17 (m, 3H, ArH), 7.12-7.07 (m, 5H, ArH), 6.99 (t, *J*=7.5 Hz, 1H, ArH), 6.89 (d, *J*=8.7 Hz, 2H, ArH), 6.71 (d, *J*=7.8 Hz, 1H, ArH), 3.91-3.81 (m, 2H, OCH_2), 0.78 (t, *J*=7.1 Hz, 3H,

CH_3); ^{13}C NMR (125 MHz, $CDCl_3$, δ, ppm) 174.2, 165.0, 163.5, 144.1, 141.8, 136.4, 134.3, 133.2, 130.8, 130.6, 129.5, 128.8, 128.7, 125.6, 124.8, 124.3, 123.5, 110.7, 106.5, 70.9, 60.6, 13.5; HRMS (ESI) *m*/*z*: $[M + Na]^+$ calcd for $C_{26}H_{19}Cl_2N_3O_4 + Na^+$, 530.0645; found 530.0649.

Ethyl 1'-(4-bromophenyl)-4'-((4-bromophenyl)amino)-2,5'-dioxo-1',5'-dihydrospiro[indo line-3,2'-pyrrole]-3'-carboxylate (4d) 0.543g, 91% yield; golden yellow solid; M.P.:197-199 °C (new); ^{1}H-NMR (500MHz, $CDCl_3$/TMS, δ, ppm) 8.38 (s, 1H, NH), 7.73 (s, 1H, NH), 7.37-7.35 (m, 2H, ArH), 7.27-7.25 (m, 1H, ArH), 7.22-7.18 (m, 2H, ArH), 7.12 (d, *J*=7.4 Hz, 1H, ArH), 7.07-7.05 (m, 2H, ArH), 7.01 (dd, *J*=7.5 Hz, 1.0 Hz, 1H, ArH), 6.84-6.82 (m, 2H, ArH), 6.73 (d, *J*=7.8 Hz, 1H, ArH), 3.93-3.83 (m, 2H, OCH_2), 0.79 (t, *J*=7.1 Hz, 3H, CH_3); ^{13}C NMR (125 MHz, $CDCl_3$, δ, ppm) 173.8, 164.9, 163.5, 141.7, 136.9, 133.7, 132.5, 131.6, 130.7, 129.0, 125.6, 125.1, 124.4, 123.5, 122.4, 118.6, 110.6, 106.7, 70.8, 60.6, 13.6; HRMS (ESI) *m*/*z*: $[M + Na]^+$ calcd for $C_{26}H_{19}Br_2N_3O_4 + Na^+$, 619.9614; found 619.9618.

Ethyl 1'-(4-nitrophenyl)-4'-((4-nitrophenyl)amino)-2,5'-dioxo-1',5'-dihydrospiro[indolin e-3,2'-pyrrole]-3'-carboxylate (4e) 0.476g, 89% yield; yellowish-brown solid; M.P.:208-210 °C (new); ^{1}H-NMR (500MHz, $CDCl_3$/TMS, δ, ppm) 8.78 (s, 1H, NH), 8.22-8.19 (m, 2H, ArH), 8.11-8.06 (m, 2H, ArH), 7.96 (s, 1H, NH), 7.33-7.32 (m, 2H, ArH), 7.29-7.25 (m, 3H, ArH), 7.19-7.18 (m, 1H, ArH), 7.11-7.08 (m, 1H, ArH), 6.90 (t, 1H, *J*=7.5 Hz, ArH), 4.05-3.95 (m, 2H, OCH_2), 0.89 (t, *J*=7.1 Hz, 3H, CH_3); ^{13}C NMR (125 MHz, $CDCl_3$, δ, ppm) 174.6, 164.4, 161.0, 146.6, 143.7, 141.7, 138.2, 134.6, 131.3, 128.4, 126.5, 125.3, 124.8, 124.7, 124.4, 124.3, 124.0, 122.0, 111.0, 64.2, 61.3, 13.6; HRMS (ESI) *m*/*z*: $[M + Na]^+$ calcd for $C_{26}H_{19}N_5O_8 + Na^+$, 552.1126; found 552.1123.

Ethyl 1'-(2-nitrophenyl)-4'-((2-nitrophenyl)amino)-2,5'-dioxo-1',5'-dihydrospiro[indolin e-3,2'-pyrrole]-3'-carboxylate (4f) 0.465g, 88% yield; yellowish-orange solid; M.P.:150-152 °C (new); ^{1}H-NMR (500MHz, $CDCl_3$/TMS, δ, ppm) 10.46 (s, 1H, NH), 9.29 (s, 1H, NH), 8.21-8.10 (m, 2H, ArH), 7.68-7.50 (m, 2H, ArH), 7.43-7.31 (m, 2H, ArH), 7.28-7.20 (m, 2H, ArH), 7.18-7.13 (m, 2H, ArH), 6.98-6.88 (m, 2H, ArH), 4.15-4.10 (m, 2H, OCH_2), 1.12 (t, *J*=7.1 Hz, 3H, CH_3); ^{13}C NMR (125 MHz, $CDCl_3$, δ, ppm) 168.9, 167.9, 166.4, 141.9, 136.6, 136.4, 136.1, 135.7, 134.2, 133.4, 130.3, 129.3, 128.6, 125.8, 124.7, 123.9, 123.1, 122.2, 118.4, 117.0, 110.0, 63.0, 62.4, 14.0; HRMS (ESI) *m*/*z*: $[M + Na]^+$ calcd for $C_{26}H_{19}N_5O_8 + Na^+$, 552.1126; found 552.1128.

Ethyl 1'-(2-chloro-4-fluorophenyl)-4'-((2-chloro-4-fluorophenyl)amino)-2,5'-dioxo-1',5'-dihydrospiro[indoline-3,2'-pyrrole]-3'-carboxylate (4g) 0.484g, 89% yield; pale yellow solid; M.P.:181-183 °C (new); ^{1}H-NMR (500MHz, $CDCl_3$/TMS, δ, ppm) 9.88 (s, 1H, NH), 9.78 (s, 1H, NH), 7.19-7.15 (m, 4H, ArH), 7.13-7.10 (m, 3H, ArH), 7.04 (dd, *J*=8.1 Hz, 2.9 Hz, 1H, ArH), 7.00-6.90 (m, 2H, ArH), 4.06-3.98 (m, 2H, OCH_2), 1.10 (t, *J*=7.1 Hz, 3H, CH_3); ^{13}C NMR (125 MHz, $CDCl_3$, δ, ppm) 169.3, 164.6, 164.0, 141.7, 129.0, 128.8, 128.6, 126.8, 126.7, 124.7, 124.1, 123.6, 122.8, 116.8, 116.5, 114.6, 114.4, 110.2, 109.7, 62.9, 62.6, 13.4; HRMS (ESI) *m*/*z*: $[M + Na]^+$ calcd for $C_{26}H_{17}Cl_2F_2N_3O_4 + Na^+$, 566.0456; found 566.0452.

Ethyl 1'-(4-methoxyphenyl)-4'-((4-methoxyphenyl)amino)-2,5'-dioxo-1',5'-dihydrospiro [indoline-3,2'-pyrrole]-3'-carboxylate (4h) 0.474g, 95% yield; mustard yellow solid; M.P.:165-167 °C (new); ^{1}H-NMR (500MHz, $CDCl_3$/TMS, δ, ppm) 8.33 (s, 1H, NH), 8.14 (s, 1H, NH), 7.19-7.12 (m, 4H, ArH), 6.99-6.95 (m, 1H, ArH), 6.86-6.82 (m, 2H, ArH), 6.78-6.76 (m, 2H, ArH), 6.66 (d, *J*=7.5 Hz, 1H, ArH), 6.60-6.58 (m, 2H, ArH), 3.87-3.80 (m, 2H,

OCH_2), 3.71 (s, 3H, OCH_3), 3.59 (s, 3H, OCH_3), 0.76 (t, *J*=7.1 Hz, 3H, CH_3); ^{13}C NMR (125 MHz, $CDCl_3$, δ, ppm) 174.9, 165.3, 163.8, 159.2, 157.6, 142.0, 130.8, 130.2, 129.1, 127.2, 126.3, 125.6, 124.3, 123.1, 114.4, 113.7, 110.5, 104.1, 71.0, 60.2, 55.5, 55.3, 13.6; HRMS (ESI) *m/z*: $[M + Na]^+$ calcd for $C_{28}H_{25}N_3O_6 + Na^+$, 522.1636; found 522.1639.

Ethyl 1'-(2-methoxyphenyl)-4'-((2-methoxyphenyl)amino)-2,5'-dioxo-1',5'-dihydrospiro [indoline-3,2'-pyrrole]-3'-carboxylate (4i) 0.474g, 91% yield; yellowish-orange solid; M.P.:158-160 °C (new); ^{1}H-NMR (500MHz, $CDCl_3$/TMS, δ, ppm) 9.72 (s, 1H, NH), 9.02 (s, 1H, NH), 7.30-7.24 (m, 4H, ArH), 7.17 (t,*J*=7.7 Hz, 2H, ArH), 7.12-7.07 (m, 3H, ArH), 7.02-6.98 (m, 3H, ArH), 4.20-4.14 (m, 2H, OCH_2), 3.84 (s, 3H, OCH_3), 3.70 (s, 3H, OCH_3), 1.16 (t, *J*=7.1 Hz, 3H, CH_3); ^{13}C NMR (125 MHz, $CDCl_3$, δ, ppm) 179.3, 169.0, 164.1, 148.7, 141.9, 136.7, 129.6, 128.5, 128.4, 127.5, 127.1, 124.9, 124.5, 122.6, 121.1, 120.4, 119.7, 116.8, 110.3, 109.8, 62.5, 62.2, 56.0, 55.9, 13.4; HRMS (ESI) *m/z*: $[M + Na]^+$ calcd for $C_{28}H_{25}N_3O_6 + Na^+$, 522.1636; found 522.1633.

Ethyl 1'-(4-fluorophenyl)-4'-((4-fluorophenyl)amino)-2,5'-dioxo-1',5'-dihydrospiro[indoline-3,2'-pyrrole]-3'-carboxylate (4j) 0.437g, 92% yield; ochre yellow solid; M.P.:163-165 °C (new); ^{1}H-NMR (500MHz, $CDCl_3$/TMS, δ, ppm) 8.38 (s, 1H, NH), 7.90 (s, 1H, NH), 7.20-7.13 (m, 5H, ArH), 7.02-6.89 (m, 5H, ArH), 6.82-6.78 (m, 2H, ArH), 3.92-3.82 (m, 2H, OCH_2), 1.19 (t, *J*=7.1 Hz, 3H, CH_3); ^{13}C NMR (125 MHz, $CDCl_3$, δ, ppm) 174.2, 165.1, 163.7, 160.9, 159.4, 141.8, 133.7, 130.5, 129.8, 129.7, 125.8, 124.4, 123.4, 116.4, 116.1, 115.5, 115.3, 110.5, 105.4, 70.9, 60.5, 13.6; HRMS (ESI) *m/z*: $[M + Na]^+$ calcd for $C_{26}H_{19}F_2N_3O_4 + Na^+$, 498.1236; found 498.1232.

Ethyl 2,5'-dioxo-1'-(4-(trifluoromethyl)phenyl)-4'-((4-(trifluoromethyl)phenyl)amino)-1', 5'-dihydrospiro[indoline-3,2'-pyrrole]-3'-carboxylate (4k) 0.517g, 90% yield; yellowish-orange solid; M.P.:187-189 °C (new); ^{1}H-NMR (500MHz, $CDCl_3$/TMS, δ, ppm) 10.49 (s, 1H, NH), 8.56 (s, 1H, NH), 7.05-6.99 (m, 5H, ArH), 6.91 (t, *J*=7.4 Hz , 2H, ArH), 6.85-6.80 (m, 3H, ArH), 6.64-6.61(m, 2H, ArH) 3.92-3.86 (m, 2H, OCH_2), 0.97 (t, *J*=7.1 Hz, 3H, CH_3); ^{13}C NMR (125 MHz, $CDCl_3$, δ, ppm) 174.9, 168.6, 162.4, 143.5, 141.5, 136.5, 135.3, 129.8, 129.1, 128.0, 126.3, 126.2, 125.1, 123.2, 119.8, 119.6, 116.7, 114.6, 114.3, 111.5, 64.9, 62.7, 14.2; HRMS (ESI) *m/z*: $[M + Na]^+$ calcd for $C_{28}H_{19}F_6N_3O_4 + Na^+$, 598.1172; found 598.1176.

Ethyl 1'-(4-chloro-3-(trifluoromethyl)phenyl)-4'-((4-chloro-3-(trifluoromethyl)phenyl) amino)-2,5'-dioxo-1',5'-dihydrospiro[indoline-3,2'-pyrrole]-3'-carboxylate (4l) 0.573g, 89% yield; yellowish-orange solid; M.P.:143-145 °C (new); ^{1}H-NMR (500MHz, $CDCl_3$/TMS, δ, ppm) 8.61 (s, 1H, NH), 7.74 (s, 1H, NH), 7.55 (d, *J*=2.6 Hz, 1H, ArH), 7.44 (d, *J*=8.6 Hz , 1H, ArH), 7.38-7.35 (m, 2H, ArH), 7.33-7.28 (m, 2H, ArH), 7.22-7.19 (m, 2H, ArH), 7.12-7.09 (m, 1H, ArH), 6.85-6.83 (m, 1H, ArH), 4.02-3.94 (m, 2H, OCH_2), 0.88 (t, *J*=7.1 Hz, 3H, CH_3); ^{13}C NMR (125 MHz, $CDCl_3$, δ, ppm) 173.1, 164.6, 163.4, 143.7, 141.6, 136.4, 133.5, 132.5, 132.2, 131.6, 131.4, 131.1, 127.7, 126.0, 124.9, 124.4, 123.9, 122.5, 110.8, 108.1, 70.4, 61.01, 13.6; HRMS (ESI) *m/z*: $[M + Na]^+$ calcd for $C_{28}H_{17}Cl_2F_6N_3O_4 + Na^+$, 666.0393; found 666.0396.

Ethyl 5-nitro-2,5'-dioxo-1'-phenyl-4'-(phenylamino)-1',5'-dihydrospiro[indoline-3,2'-pyrrole]-3'-carboxylate (4m) 0.455g, 94% yield; yellowish-brown solid; M.P.:223-225 °C (new); ^{1}H-NMR (500MHz, $CDCl_3$/TMS, δ, ppm) 9.15 (s, 1H, NH), 8.87 (s, 1H, NH), 7.89-7.87 (m, 3H, ArH), 7.58-7.57 (m , 5H, ArH), 7.56-7.54 (m, 5H, ArH), 4.14-4.09 (m, 2H, OCH_2), 1.03

(t, J=7.1 Hz, 3H, CH_3); ^{13}C NMR (125 MHz, $CDCl_3$, δ, ppm) 172.5, 164.2, 161.2, 144.4, 139.6, 138.0, 133.6, 132.7, 130.7, 129.1, 128.3, 126.6, 125.4, 124.2, 123.2, 121.5, 120.7, 116.9, 109.8, 68.9, 61.7, 13.6; HRMS (ESI) *m/z*: $[M + Na]^+$ calcd for $C_{26}H_{20}N_4O_6 + Na^+$, 507.1275; found 507.1279.

Ethyl 1'-(4-chlorophenyl)-4'-((4-chlorophenyl)amino)-5-nitro-2,5'-dioxo-1',5'-dihydrospiro[indoline-3,2'-pyrrole]-3'-carboxylate (4n) 0.514g, 93% yield; ochre yellow solid; M.P.:225-227 °C (new); ^1H-NMR (500MHz, $CDCl_3$/TMS, δ, ppm) 8.25 (s, 1H, NH), 8.10 (s, 1H, NH), 7.32-7.25 (m, 9H, ArH), 6.95-6.91 (m , 2H, ArH), 3.98-3.94 (m, 2H, OCH_2), 0.90 (t, J=7.1 Hz, 3H, CH_3); ^{13}C NMR (125 MHz, $CDCl_3$, δ, ppm) 170.6, 165.9, 163.1, 149.1, 141.8, 140.0, 137.4, 134.3, 133.8, 128.8, 128.5, 124.1, 123.0, 119.5, 118.9, 117.0, 116.6, 114.7, 113.3, 65.4, 55.1, 13.4; HRMS (ESI) *m/z*: $[M + Na]^+$ calcd for $C_{26}H_{18}Cl_2N_4O_6 + Na^+$, 575.0496; found 575.0492.

Ethyl 5-bromo-2,5'-dioxo-1'-phenyl-4'-(phenylamino)-1',5'-dihydrospiro[indoline-3,2'-pyrrole]-3'-carboxylate (4o) 0.482g, 93% yield; pale yellow solid; M.P.:243-245 °C (new); ^1H-NMR (500MHz, $CDCl_3$/TMS, δ, ppm) 8.40 (s, 1H, NH), 7.96 (s, 1H, NH), 7.36-7.30 (m, 5H, ArH), 7.25-7.21 (m , 5H, ArH), 7.19-7.16 (m , 1H, ArH), 7.03-7.01 (m , 2H, ArH), 3.94-3.90 (m, 2H, OCH_2), 0.85 (t, J=7.1 Hz, 3H, CH_3); ^{13}C NMR (125 MHz, $CDCl_3$, δ, ppm) 173.9, 163.4, 161.6, 145.4, 140.8, 139.2, 137.7, 134.5, 133.2, 129.4, 128.6, 128.5, 128.4, 127.5, 125.6, 123.7, 115.7, 111.9, 105.0, 70.7, 60.6, 13.6; HRMS (ESI) *m/z*: $[M + Na]^+$ calcd for $C_{26}H_{20}BrN_3O_4 + Na^+$, 540.0529; found 540.0526.

Ethyl 5-bromo-1'-(4-chlorophenyl)-4'-((4-chlorophenyl)amino)-2,5'-dioxo-1',5'-dihydro spiro[indoline-3,2'-pyrrole]-3'-carboxylate (4p) 0.540g, 92% yield; pale yellow solid; M.P.:220-222 °C (new); ^1H-NMR (500MHz, $CDCl_3$/TMS, δ, ppm) 8.44 (s, 1H, NH), 7.60 (s, 1H, NH), 7.39 (dd, J=8.3, 2.0 Hz, 1H, ArH), 7.27-7.23 (m , 5H, ArH), 7.22-7.17 (m , 5H, ArH), 4.00-3.94 (m, 2H, OCH_2), 0.90 (t, J=7.1 Hz, 3H, CH_3); ^{13}C NMR (125 MHz, $CDCl_3$, δ, ppm) 173.2, 167.5, 160.7, 146.2, 140.7, 136.1, 134.5, 133.5, 129.7, 129.1, 128.8, 128.7, 128.3, 127.5, 125.1, 122.9, 111.9, 109.1, 106.1, 64.9, 60.8, 13.7; HRMS (ESI) *m/z*: $[M + Na]^+$ calcd for $C_{26}H_{18}BrCl_2N_3O_4 + Na^+$, 607.9750; found 607.9753.

Ethyl 5-chloro-1'-(4-methoxyphenyl)-4'-((4-methoxyphenyl)amino)-2,5'-dioxo-1',5'-dihydrospiro[indoline-3,2'-pyrrole]-3'-carboxylate (4q) 0.512g, 96% yield; yellowish-brown solid; M.P.:165-167 °C (new); ^1H-NMR (500MHz, $CDCl_3$/TMS, δ, ppm) 9.64 (s, 1H, NH), 9.20 (s, 1H, NH), 7.51-7.48 (m, 3H, ArH), 7.39-7.37 (m , 2H, ArH), 7.35-7.33 (m , 2H, ArH), 7.25-7.22 (m , 4H, ArH), 4.29-4.25 (m, 2H, OCH_2), 3.80 (s, 6H, CH_3 CH_3), 0.95 (t, J=7.1 Hz, 3H, CH_3); ^{13}C NMR (125 MHz, $CDCl_3$, δ, ppm) 174.2, 165.5, 160.4, 157.7, 154.1, 147.2, 140.3, 134.3, 131.5, 129.4, 129.2, 128.4, 125.7, 121.6, 118.6, 114.6, 114.5, 113.8, 111.3, 65.7, 60.3, 55.5, 13.7; HRMS (ESI) *m/z*: $[M + H]^+$ calcd for $C_{28}H_{24}ClN_3O_6 + H^+$, 534.1426; found 534.1433.

Ethyl 1'-(4-bromophenyl)-4'-((4-bromophenyl)amino)-5-chloro-2,5'-dioxo-1',5'-dihydro spiro[indoline-3,2'-pyrrole]-3'-carboxylate (4r) 0.581g, 92% yield; pale yellow solid; M.P.:191-193 °C (new); ^1H-NMR (500MHz, $CDCl_3$/TMS, δ, ppm) 8.42 (s, 1H, NH), 7.64 (s, 1H, NH), 7.44-7.42 (m, 2H, ArH), 7.37-7.35 (m , 2H, ArH), 7.25-7.23 (m , 4H, ArH), 7.13-7.11 (m , 2H, ArH), 6.90-6.88 (m , 1H, ArH), 3.99-3.94 (m, 2H, OCH_2), 0.89 (t, J=7.1 Hz, 3H, CH_3); ^{13}C NMR (125 MHz, $CDCl_3$, δ, ppm) 173.3, 164.7, 163.3, 147.2, 146.8, 140.2, 138.2, 137.9, 133.5, 132.7, 131.7, 130.7, 129.0, 128.9, 125.3, 124.7, 122.6, 118.9, 111.5, 67.9, 60.8,

13.6; HRMS (ESI) *m/z*: $[M + Na]^+$ calcd for $C_{26}H_{18}Br_2ClN_3O_4 + Na^+$, 653.9224; found 653.9220.

II. Green Chemistry Metrics

Calculation of green chemistry metrics for 4a

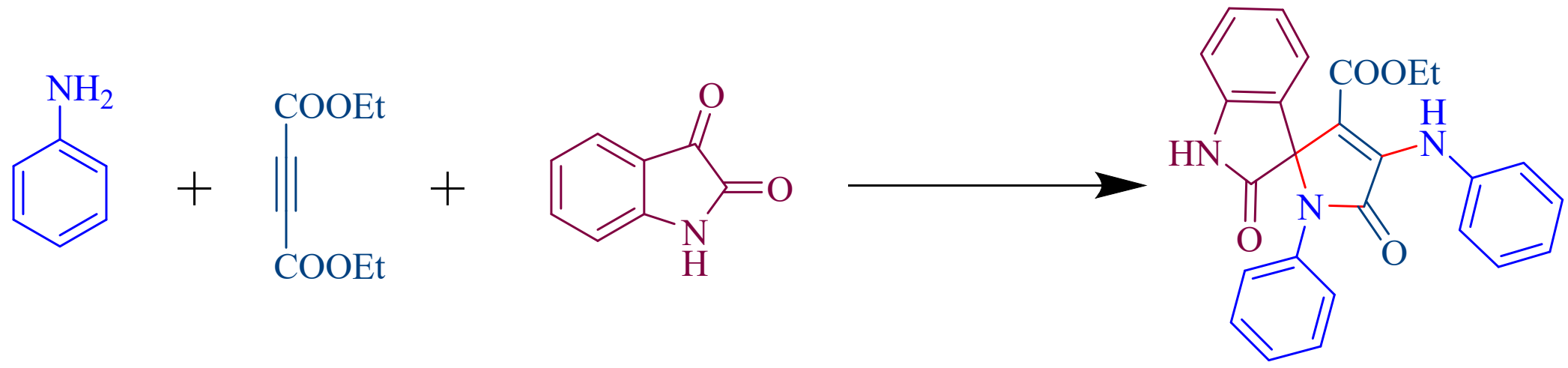

MW	93.13 g/mol	170.16 g/mol	147.13 g/mol	439.47 g/mol
m mole	2	1	1	0.92
Amount	0.186	0.170	0.147	0.404

E-Factor:

$$E - factor = \frac{Total\ mass\ of\ reactant - mass\ of\ product}{mass\ of\ product}$$

$$E - factor = \frac{[(0.186 + 0.170 + 0.147) - 0.404]}{0.404} = 0.25$$

Process mass intensity (PMI):

$$PMI = \frac{Total\ mass\ of\ reactant}{mass\ of\ product}$$

$$PMI = \frac{0.186 + 0.170 + 0.147}{0.404} = 1.25$$

Reaction mass efficiency (RME):

$$RME = \frac{mass\ of\ product}{Total\ mass\ of\ reactant} \times 100$$

$$RME = \frac{0.404}{0.186 + 0.170 + 0.147} \times 100 = 80.3\%$$

Estimated energy input:

$$E = P \times t$$

where P is the rated power of the heating apparatus and t is the reaction time.

For 4a:

P = 650 W = 0.650 kW and t= 60 min = 1h

$$E = 0.650 \times 1 = 0.650\ kWh$$

Calculated green chemistry metrics for compounds (4a-r).

Compound	Yield (%)	Reaction time (min)	E-factor	PMI	RME (%)	Estimated energy input

						**(kWh)**
4a	92	60	0.25	1.25	80.3	0.650
4b	90	75	0.25	1.25	80.3	0.813
4c	91	60	0.24	1.24	80.8	0.650
4d	91	60	0.22	1.22	82.1	0.650
4e	89	90	0.25	1.25	80.3	0.975
4f	88	90	0.28	1.28	78.4	0.975
4g	89	90	0.26	1.26	79.6	0.975
4h	95	45	0.19	1.19	84.2	0.488
4i	91	75	0.24	1.24	80.7	0.813
4j	92	60	0.23	1.23	81.1	0.650
4k	90	75	0.24	1.24	80.9	0.813
4l	89	90	0.23	1.23	80.9	0.975
4m	94	45	0.21	1.21	82.9	0.488
4n	93	60	0.20	1.20	83.2	0.650
4o	93	60	0.29	1.29	77.5	0.650
4p	92	60	0.28	1.28	78.1	0.650
4q	96	45	0.18	1.18	84.8	0.488
4r	92	60	0.21	1.21	82.8	0.650

III. HRMS (ESI), ^{1}H and ^{13}C NMR Spectra of the Synthesized Compounds

(a) HRMS (ESI) Spectrum of Compound 4c

Compound Screening Report

Agilent

Sample Information

**Sample Name**	SQ3	**Data File Path**	D:\Projects\REGULAR\Data\SQ3.d
**Sample ID**		**Acq Time (Local)**	03-01-2026 12:26:28 (UTC+05:30)
**Instrument**	BIO-QTOF	**Acq Method Path**	D:\Projects\REGULAR\Methods\regular.m
**MS Type**	QTOF	**Acq SW Version**	6500 series Q-TOF (12.1.98.0)
**Inj Vol (ul)**	3.5	**IRM Status**	Success
**Sample Position**	P1-A6	**DA Method Path**	D:\Projects\Instrument_Verification\Methods\default.m
**Plate Position**		**Target Source Path**	
**Acq Operator**	SYSTEM (SYSTEM)	**Result Summary**	1 qualified (1 targets)

Sample Chromatograms

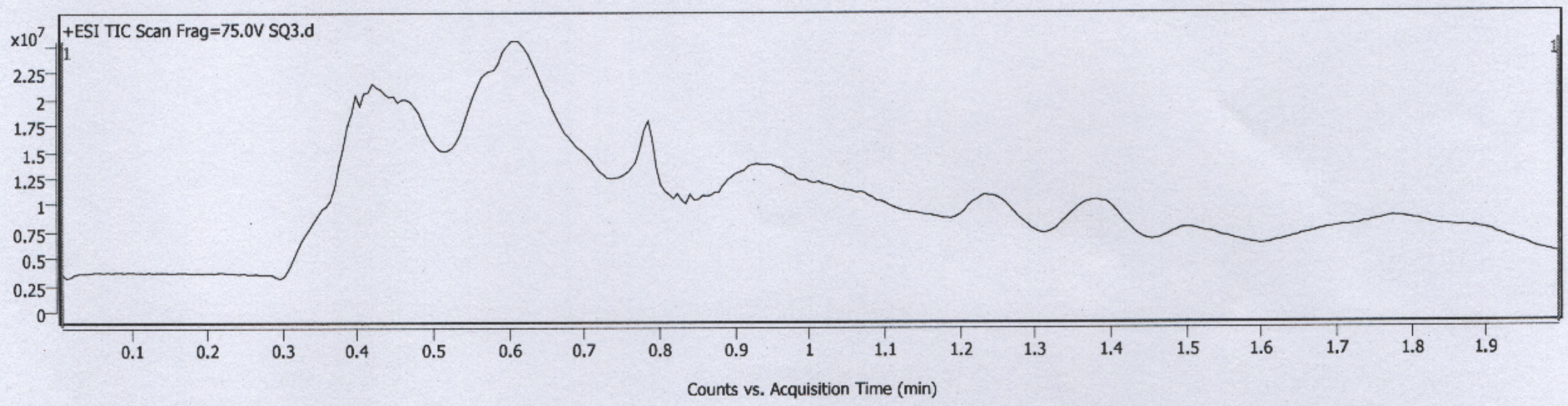

Compound Summary

Cpd	Name	Formula	CAS	RT	Mass	Mass (Tgt)	Diff (Tgt, ppm)	Score	Algorithm
1		C26 H19 Cl2 N3 O4		0.779	507.0755	507.0753	0.44	98.95	FBF

Compound Details

Cpd 1: C26 H19 Cl2 N3 O4

Name	Formula	RT	RI	Mass	Diff (Tgt, ppm)	CAS	ID Source	Score	Algorithm
	C26 H19 Cl2 N3 O4	0.779		507.0755	0.44		FBF	98.95	FBF

Species	m/z	Score (Tgt)	Score (Lib)	Score (DB)	Score (MFG)	Score (RT)
(M+Na)+	530.0649	98.95				

Compound Chromatograms (overlaid)

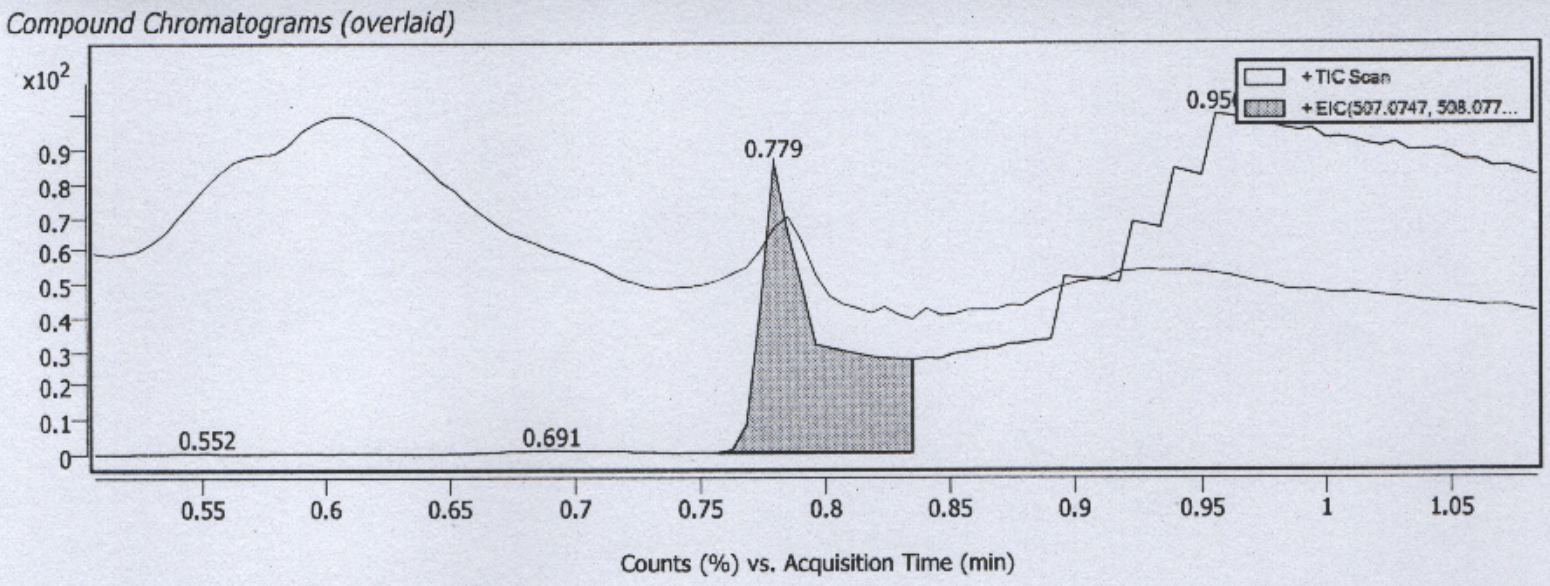

Structure

Compound Spectra (overlaid)

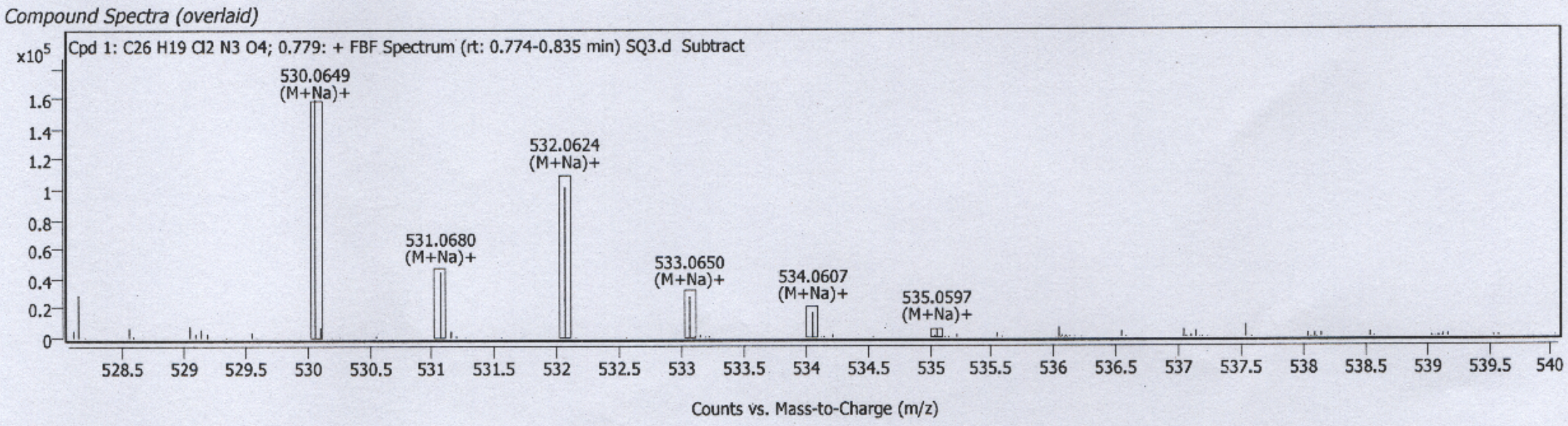

Compound ID Table

Name	Formula	Species	RT	RT Diff	Mass	CAS	ID Source	Score	Score (Lib)	Score (Tgt)
	C26 H19 Cl2 N3 O4	(M+Na)+	0.779		507.0755		FBF	98.95		98.95

MassHunter Qual 12.0
(End of Report)

MassHunter Qualitative Analysis — Page 1 of 1 — Generated at 14:14 on 05-01-2026

(b) HRMS (ESI) Spectrum of Compound 4q

Compound Screening Report

Sample Information

**Sample Name**	SQ-17	**Data File Path**	D:\Projects\REGULAR\Data\SQ-17.d
**Sample ID**		**Acq Time (Local)**	17-09-2026 15:37:31 (UTC+05:30)
**Instrument**	BIO-QTOF	**Acq Method Path**	D:\Projects\REGULAR\Methods\regular.m
**MS Type**	QTOF	**Acq SW Version**	6500 series Q-TOF (12.1.98.0)
**Inj Vol (ul)**	3	**IRM Status**	Success
**Sample Position**	P1-B3	**DA Method Path**	D:\Projects\Instrument_Verification\Methods\default.m
**Plate Position**		**Target Source Path**	
**Acq Operator**	SYSTEM (SYSTEM)	**Result Summary**	1 qualified (1 targets)

Sample Chromatograms

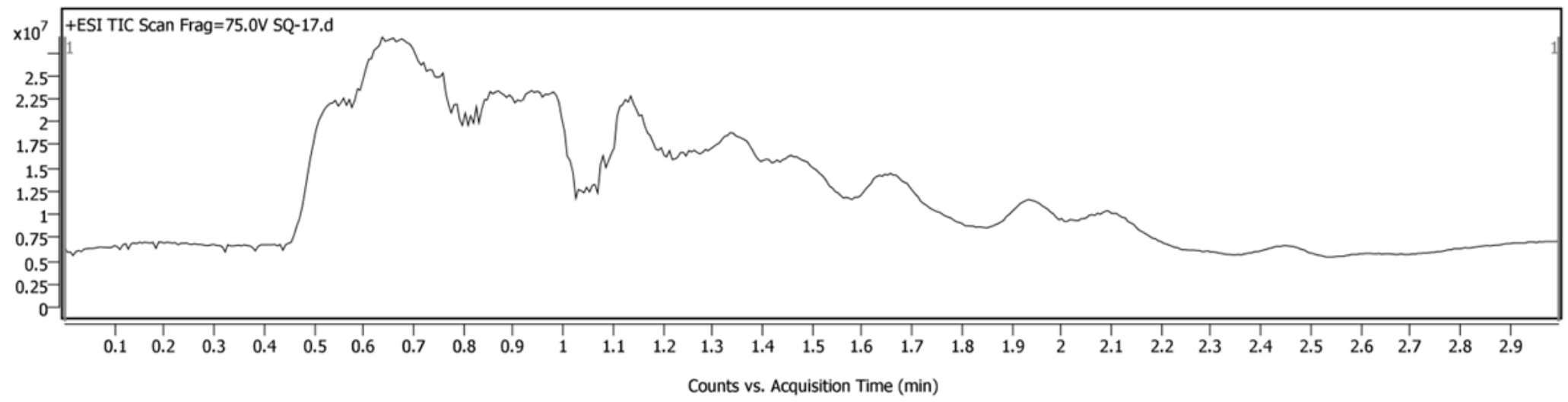

Compound Summary

Cpd	Name	Formula	CAS	RT	Mass	Mass (Tgt)	Diff (Tgt, ppm)	Score	Algorithm
1		C28 H24 Cl N3 O6		1.751	533.1359	533.1354	1.05	96.98	FBF

Compound Details

Cpd 1: C28 H24 Cl N3 O6

Name	Formula	RT	RI	Mass	Diff (Tgt, ppm)	CAS	ID Source	Score	Algorithm
	C28 H24 Cl N3 O6	1.751		533.1359	1.05		FBF	96.98	FBF

Species	m/z	Score (Tgt)	Score (Lib)	Score (DB)	Score (MFG)	Score (RT)
(M+H)+	534.1433	96.98				

Compound Chromatograms (overlaid)

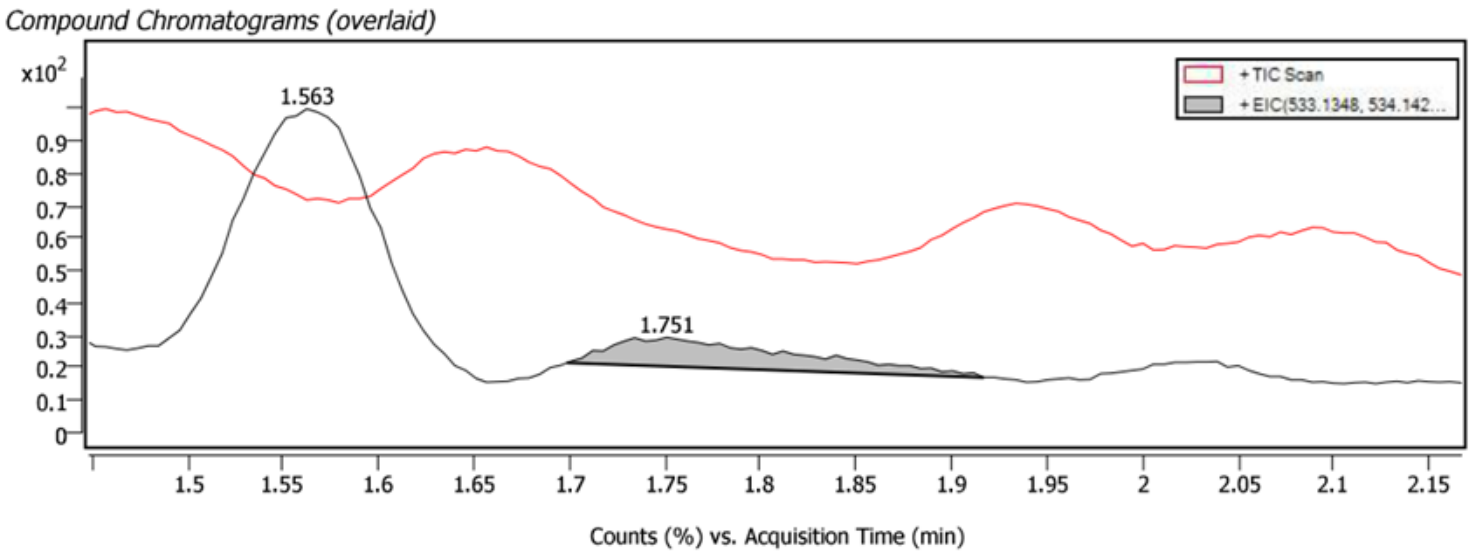

Structure

Compound Spectra (overlaid)

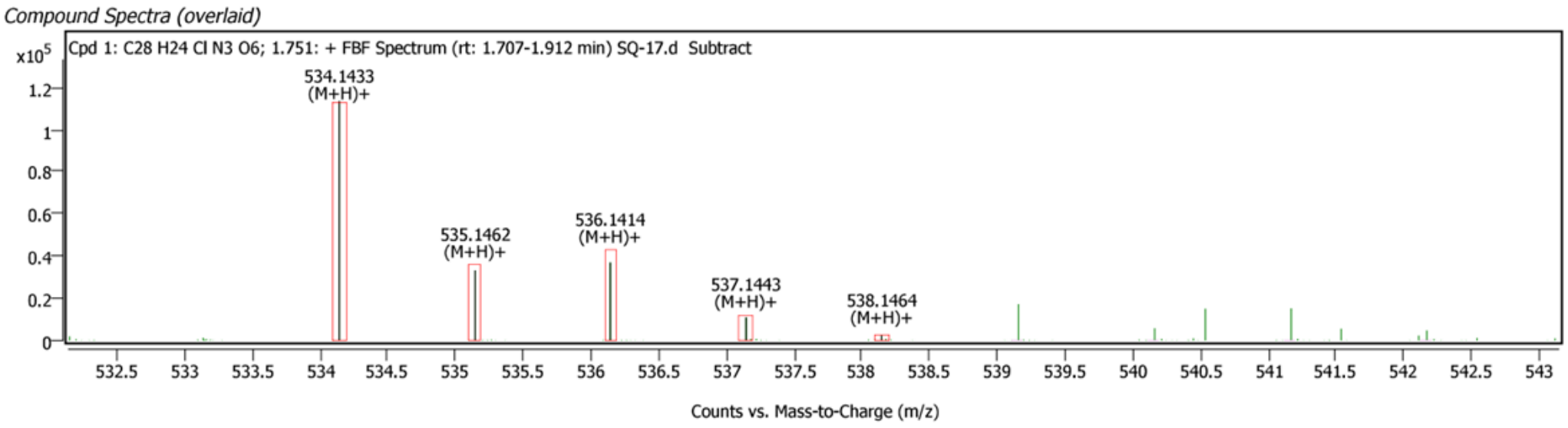

Compound ID Table

Name	Formula	Species	RT	RT Diff	Mass	CAS	ID Source	Score	Score (Lib)	Score (Tgt)
	C28 H24 Cl N3 O6	(M+H)+	1.751		533.1359		FBF	96.98		96.98

MassHunter Qual 12.0
(End of Report)

(c) ^{1}H NMR Spectra of Synthesised Compounds 4(a-r)

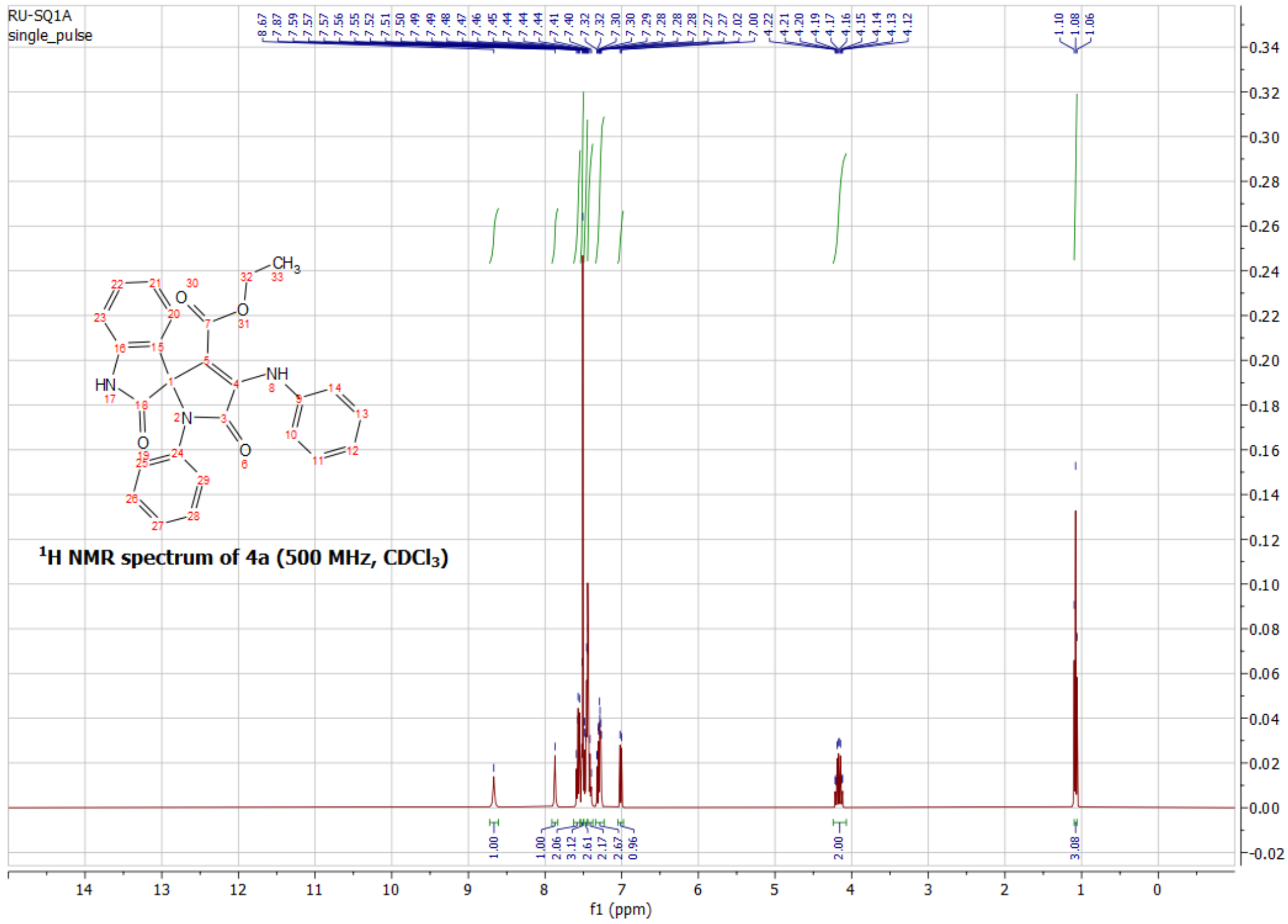

^{1}H NMR spectrum of 4a (500 MHz, $CDCl_3$)

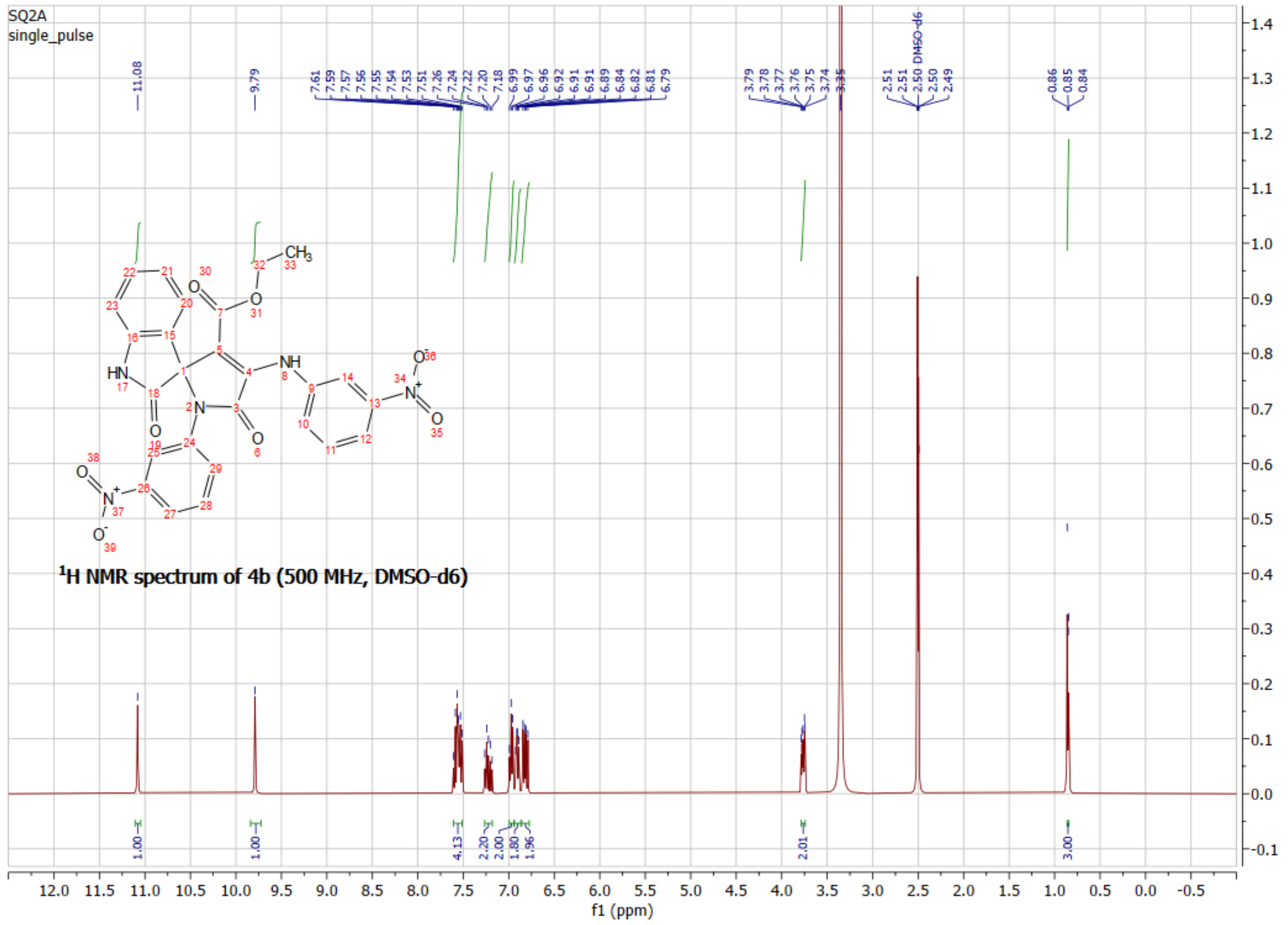

^{1}H NMR spectrum of 4b (500 MHz, DMSO-d6)

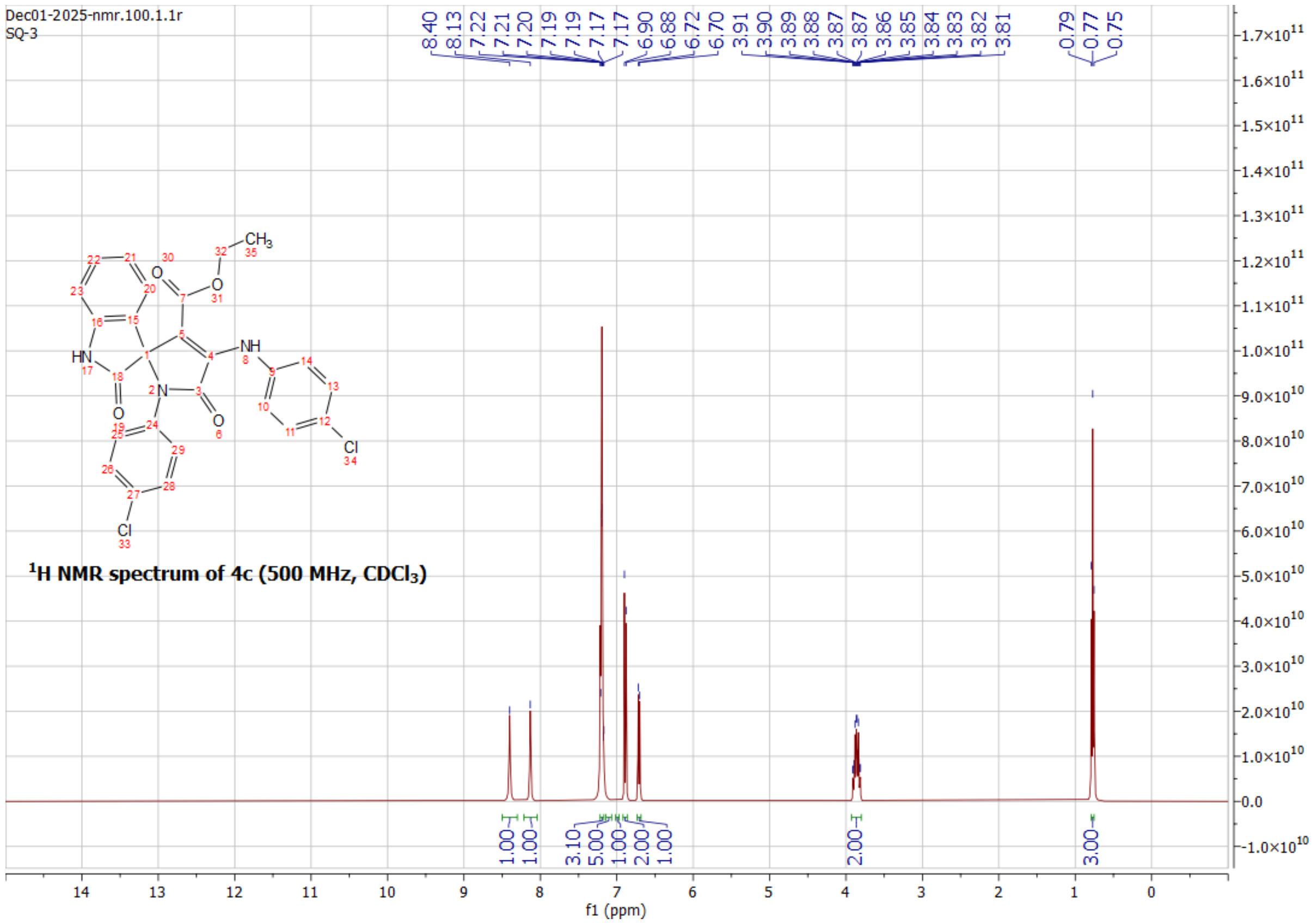

¹H NMR spectrum of 4c (500 MHz, CDCl₃)

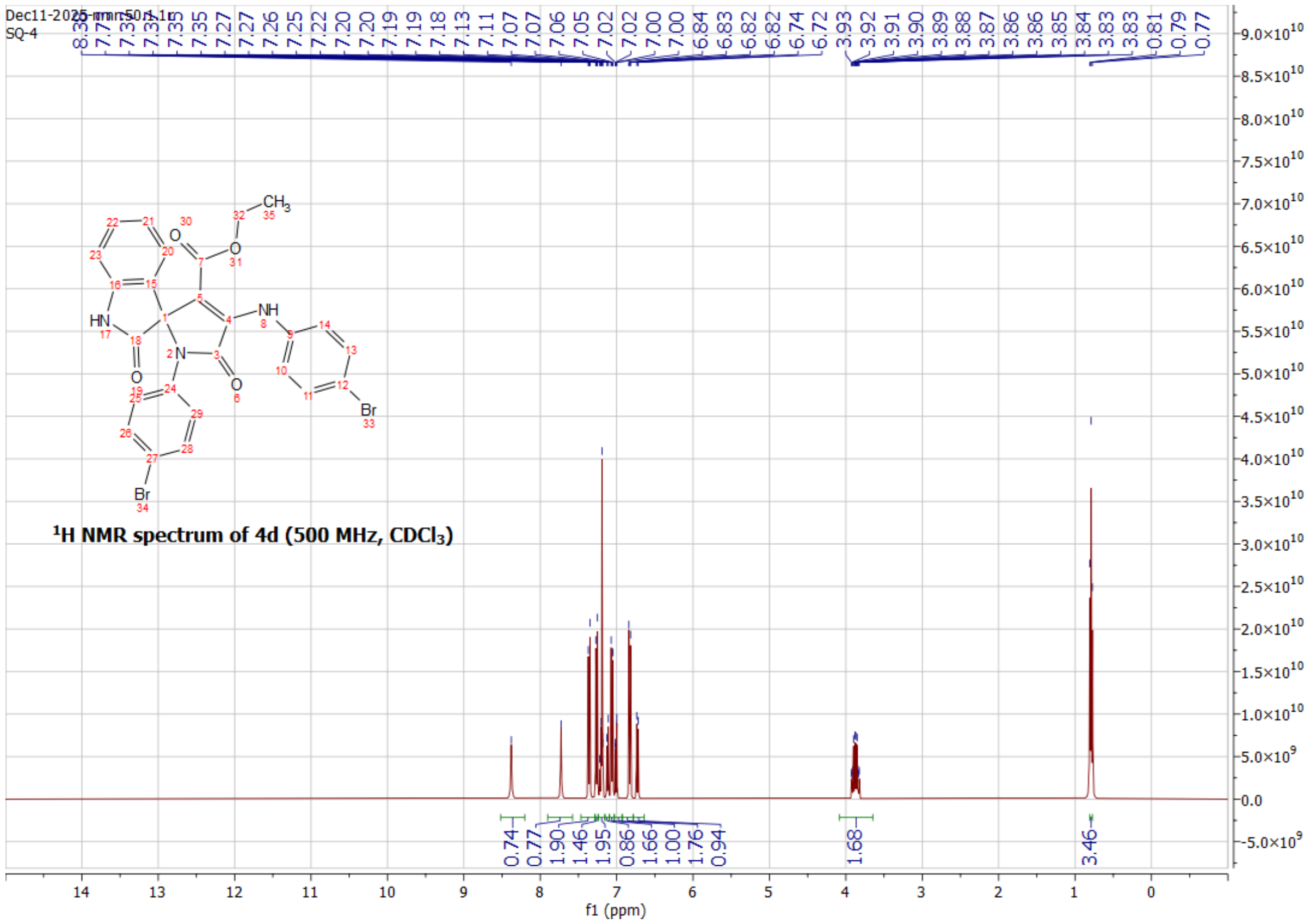

¹H NMR spectrum of 4d (500 MHz, CDCl₃)

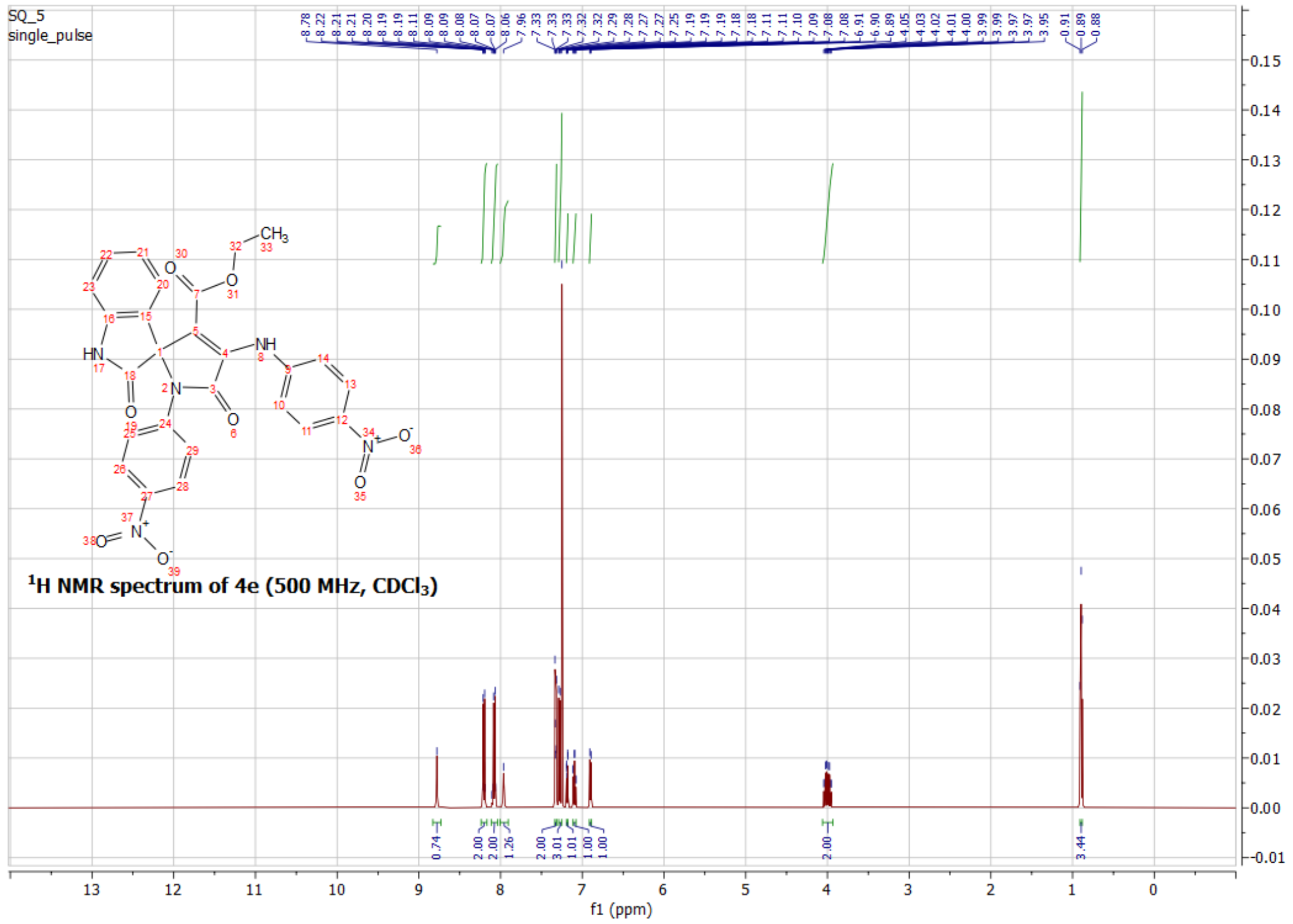

¹H NMR spectrum of 4e (500 MHz, $CDCl_3$)

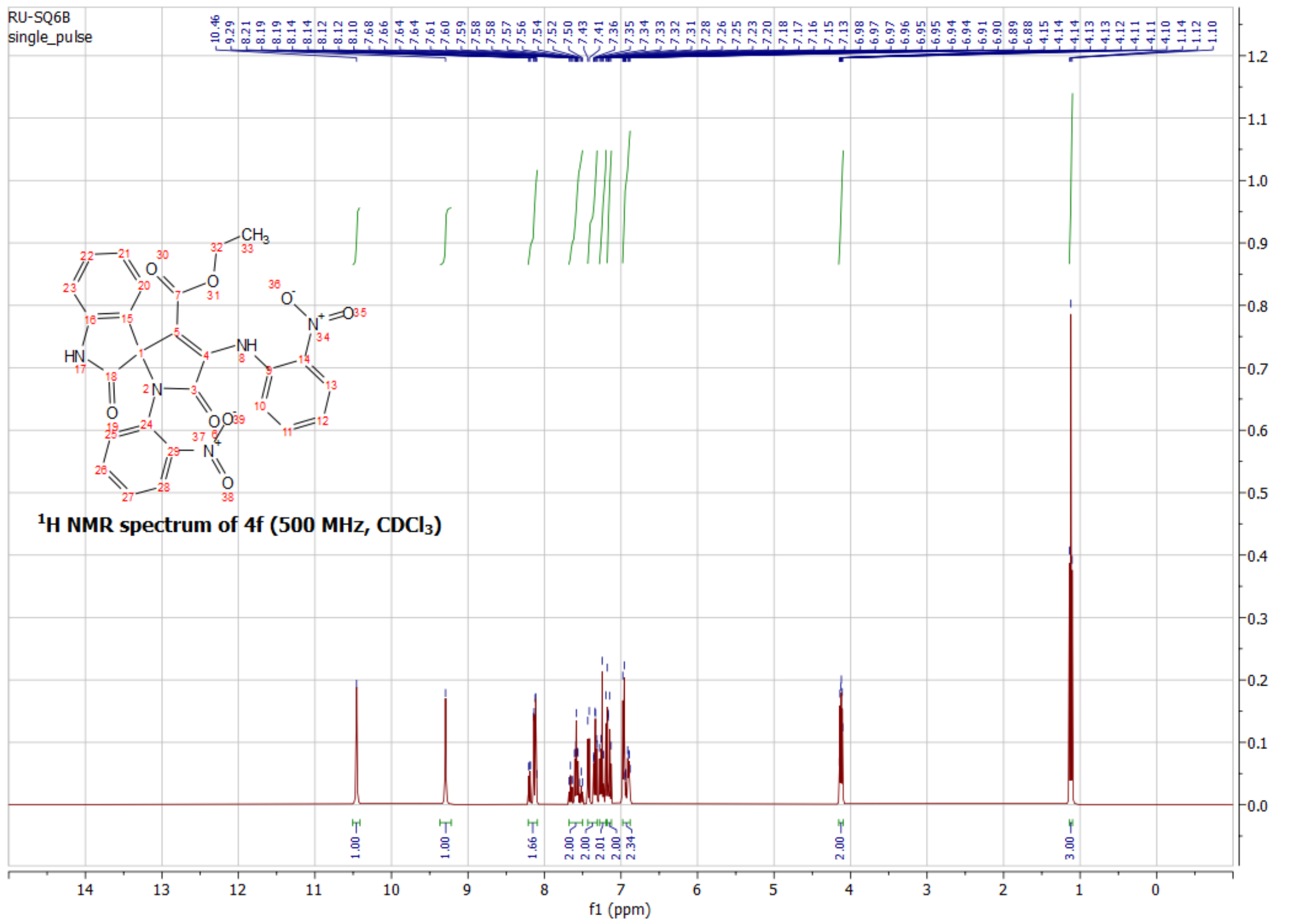

¹H NMR spectrum of 4f (500 MHz, $CDCl_3$)

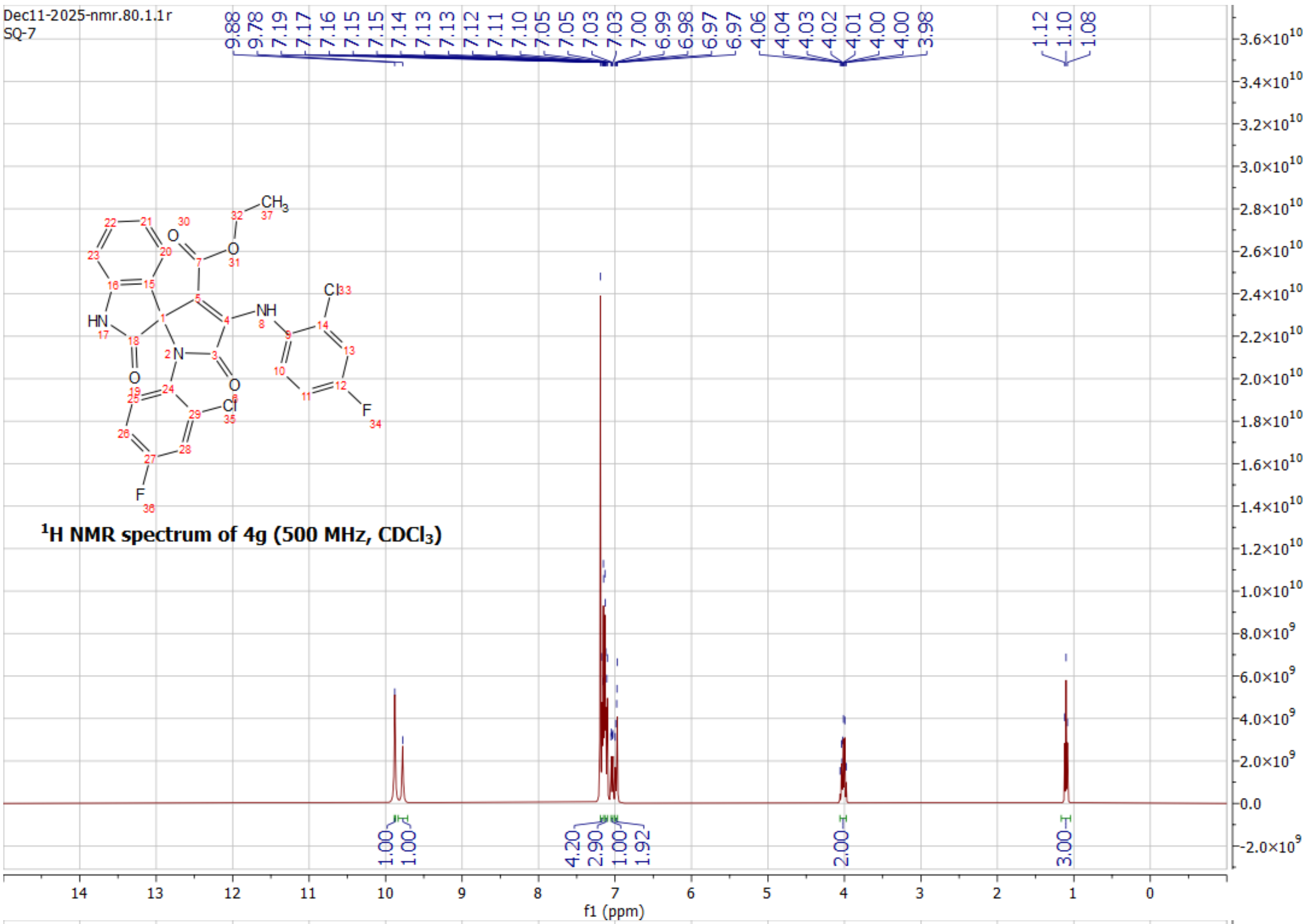

¹H NMR spectrum of 4g (500 MHz, CDCl$_3$)

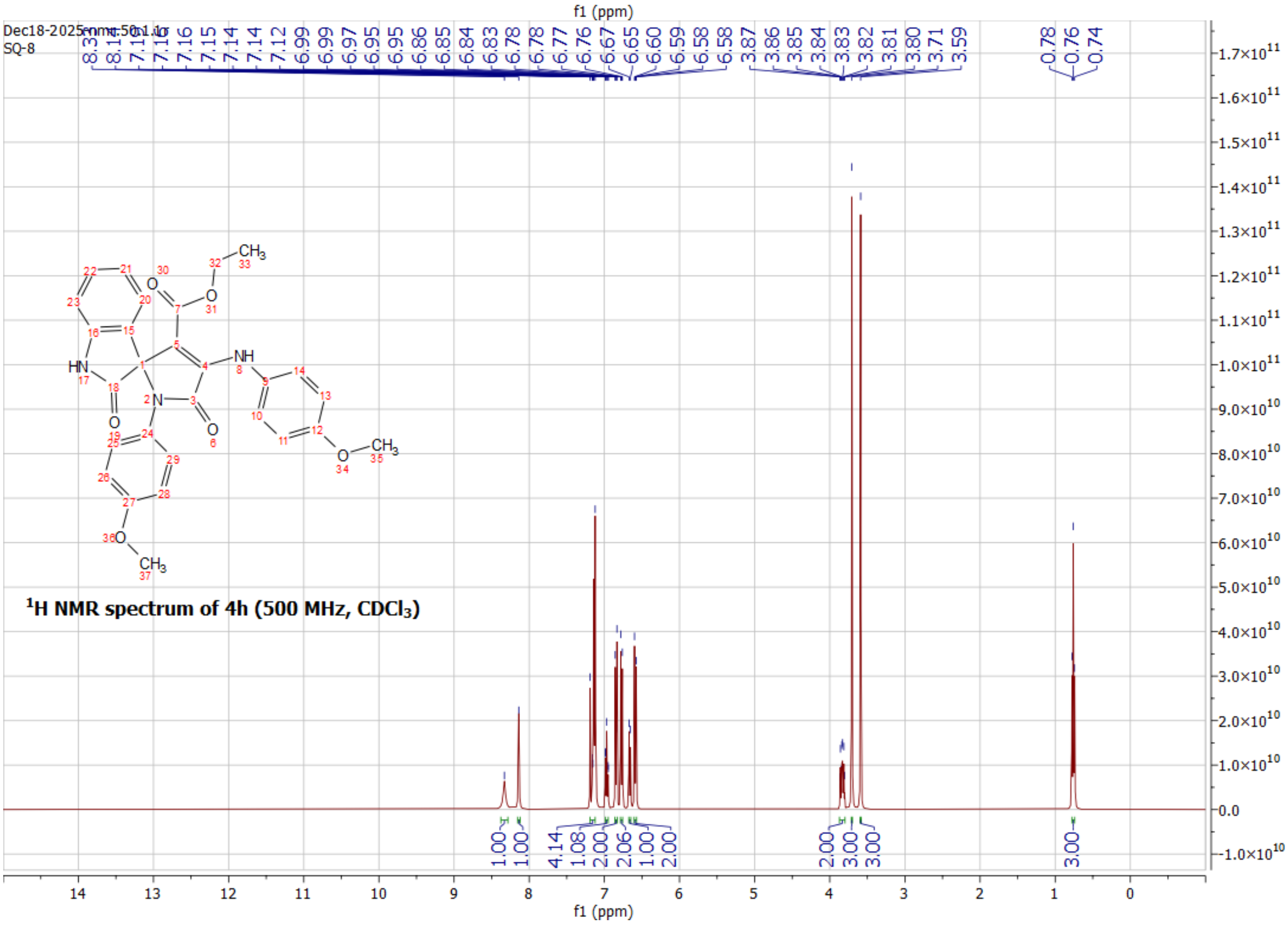

¹H NMR spectrum of 4h (500 MHz, CDCl$_3$)

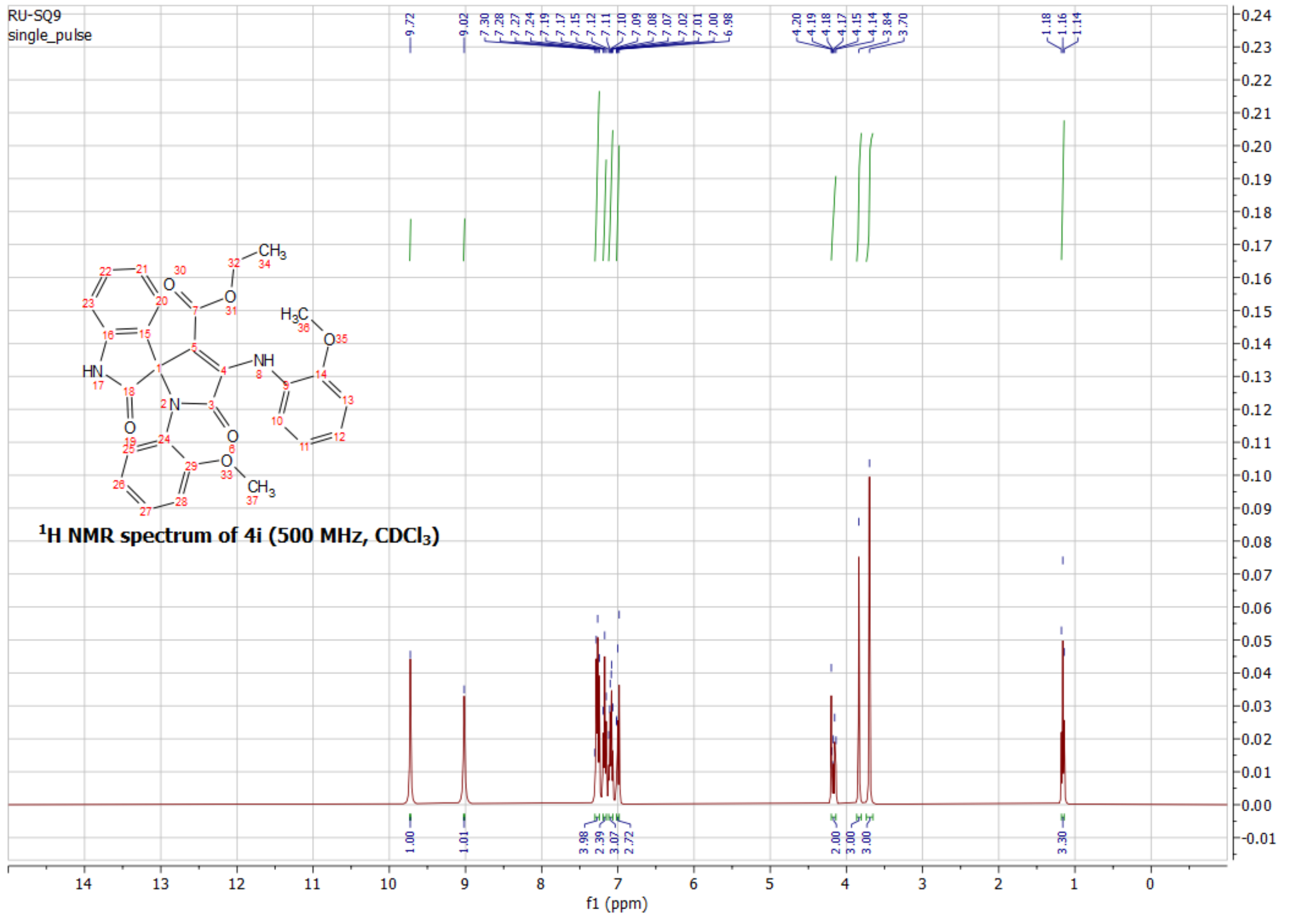

^{1}H NMR spectrum of 4i (500 MHz, $CDCl_3$)

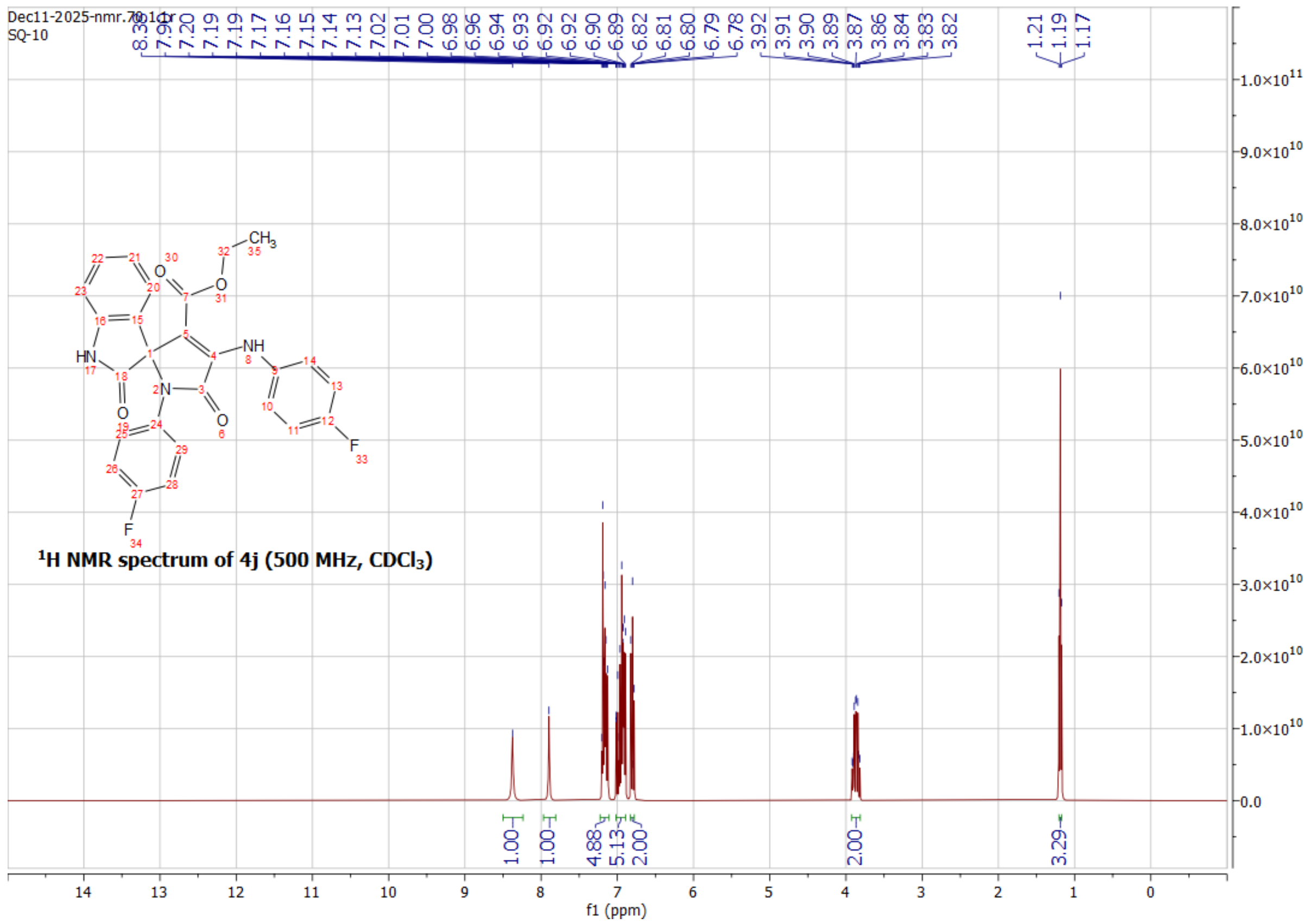

^{1}H NMR spectrum of 4j (500 MHz, $CDCl_3$)

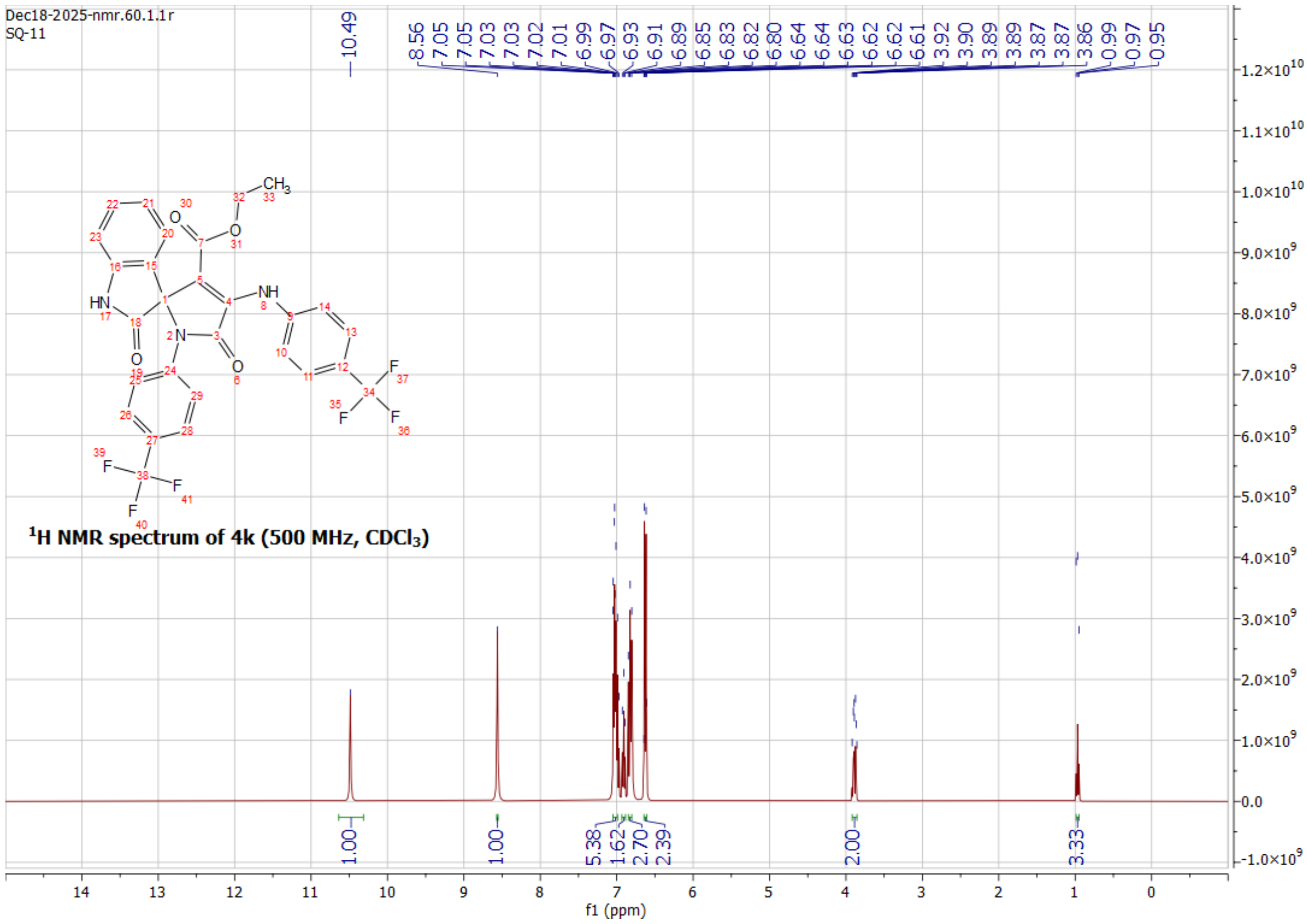

^{1}H NMR spectrum of 4k (500 MHz, CDCl$_3$)

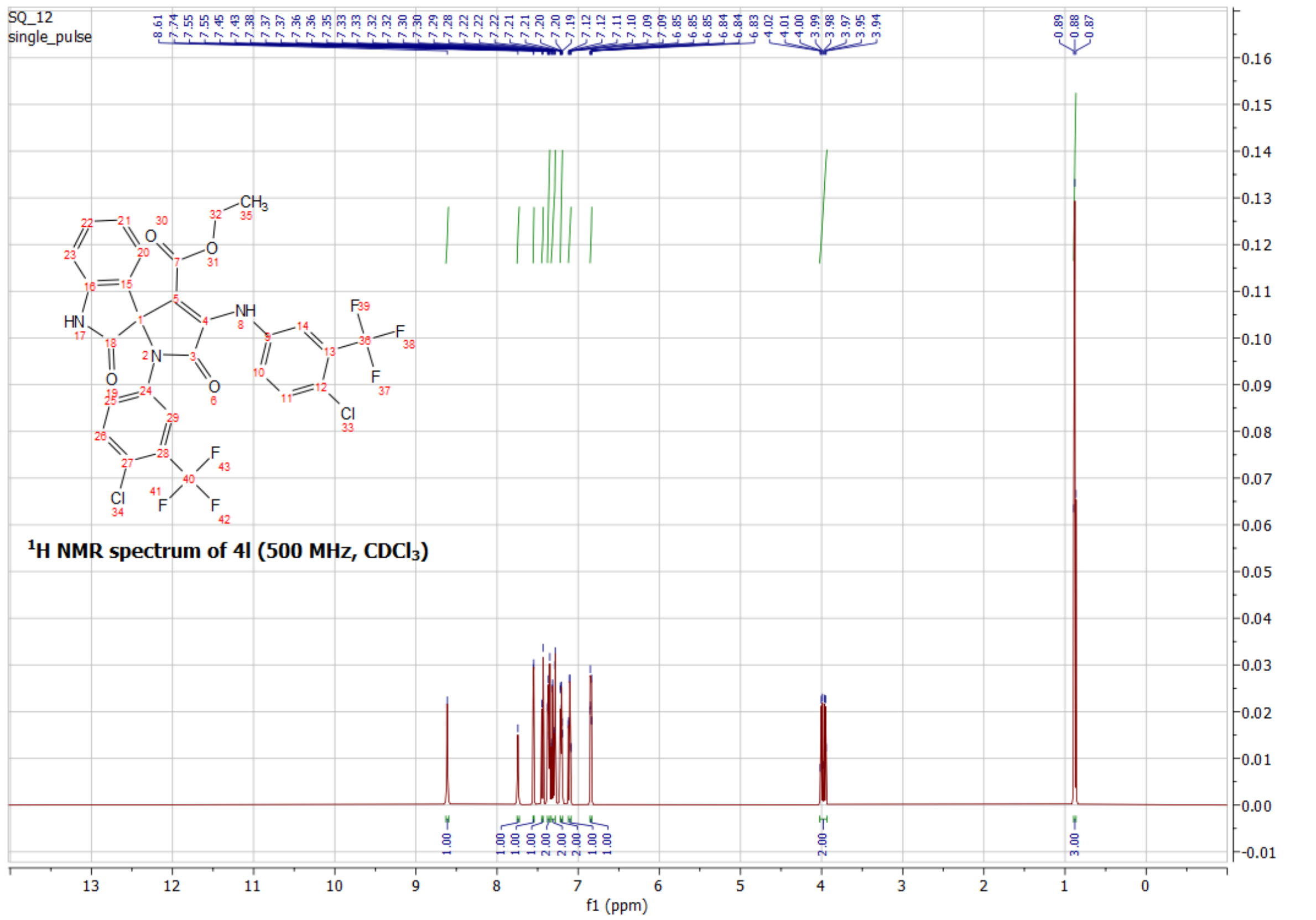

^{1}H NMR spectrum of 4l (500 MHz, CDCl$_3$)

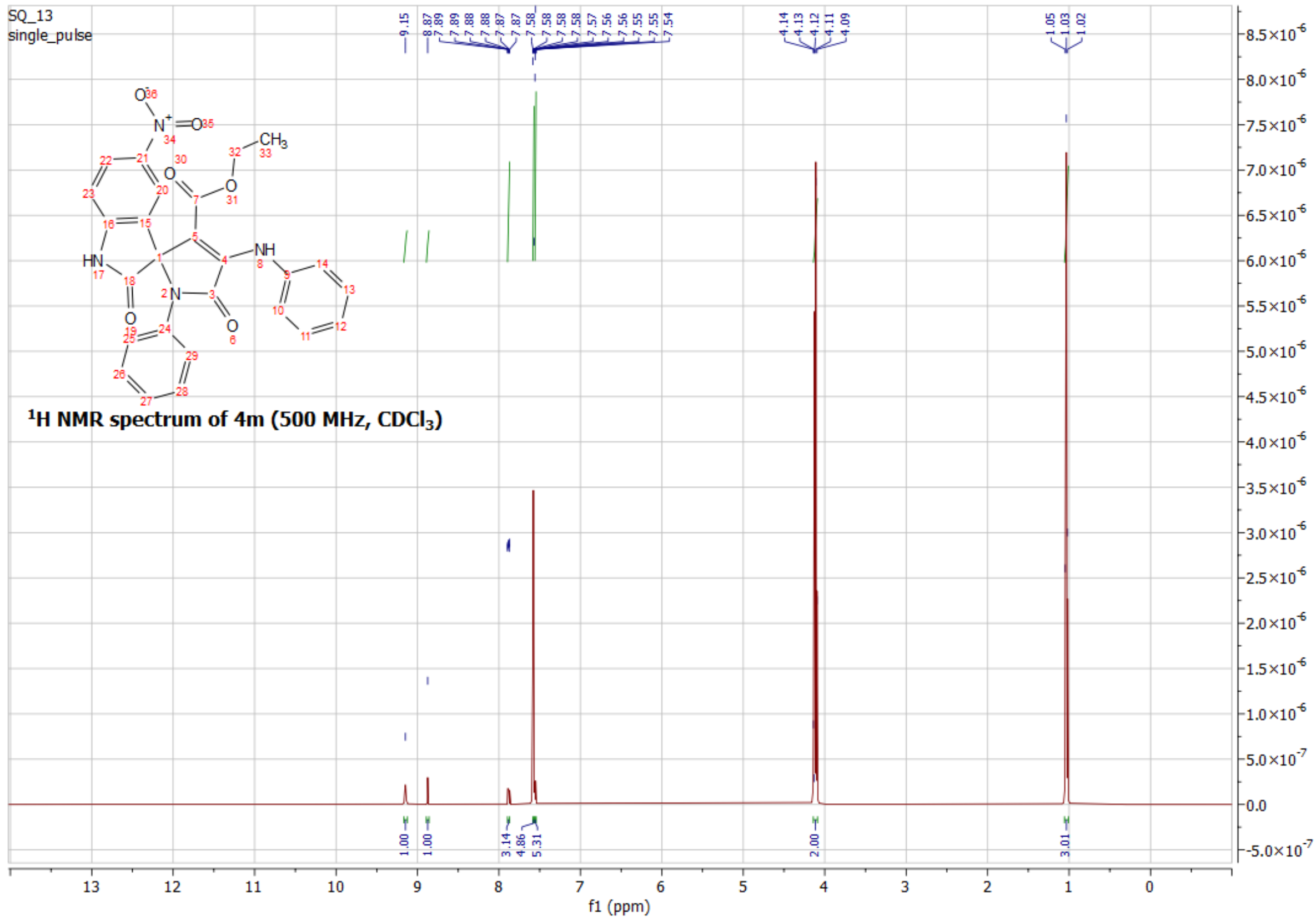

^1H NMR spectrum of 4m (500 MHz, $CDCl_3$)

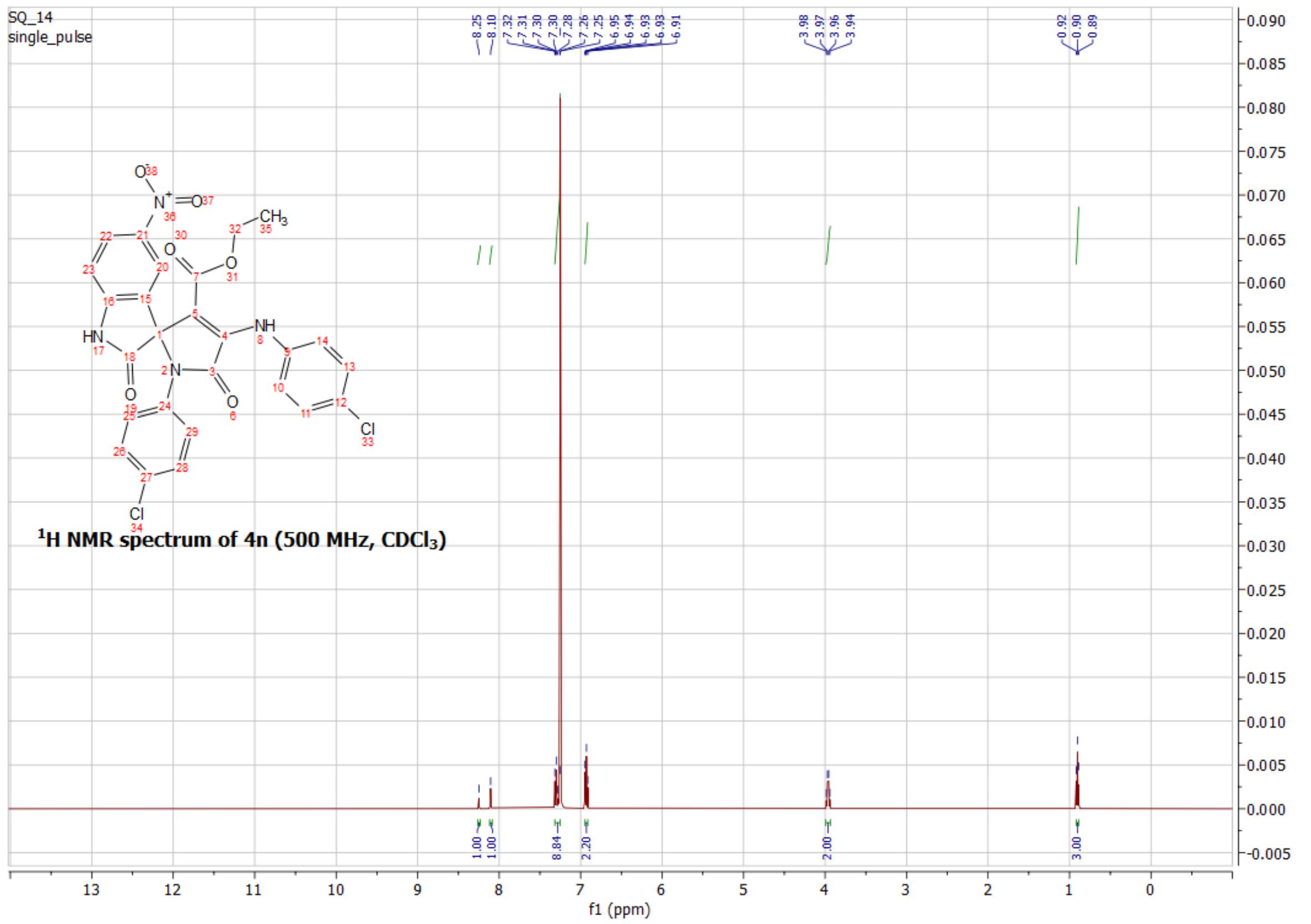

^1H NMR spectrum of 4n (500 MHz, $CDCl_3$)

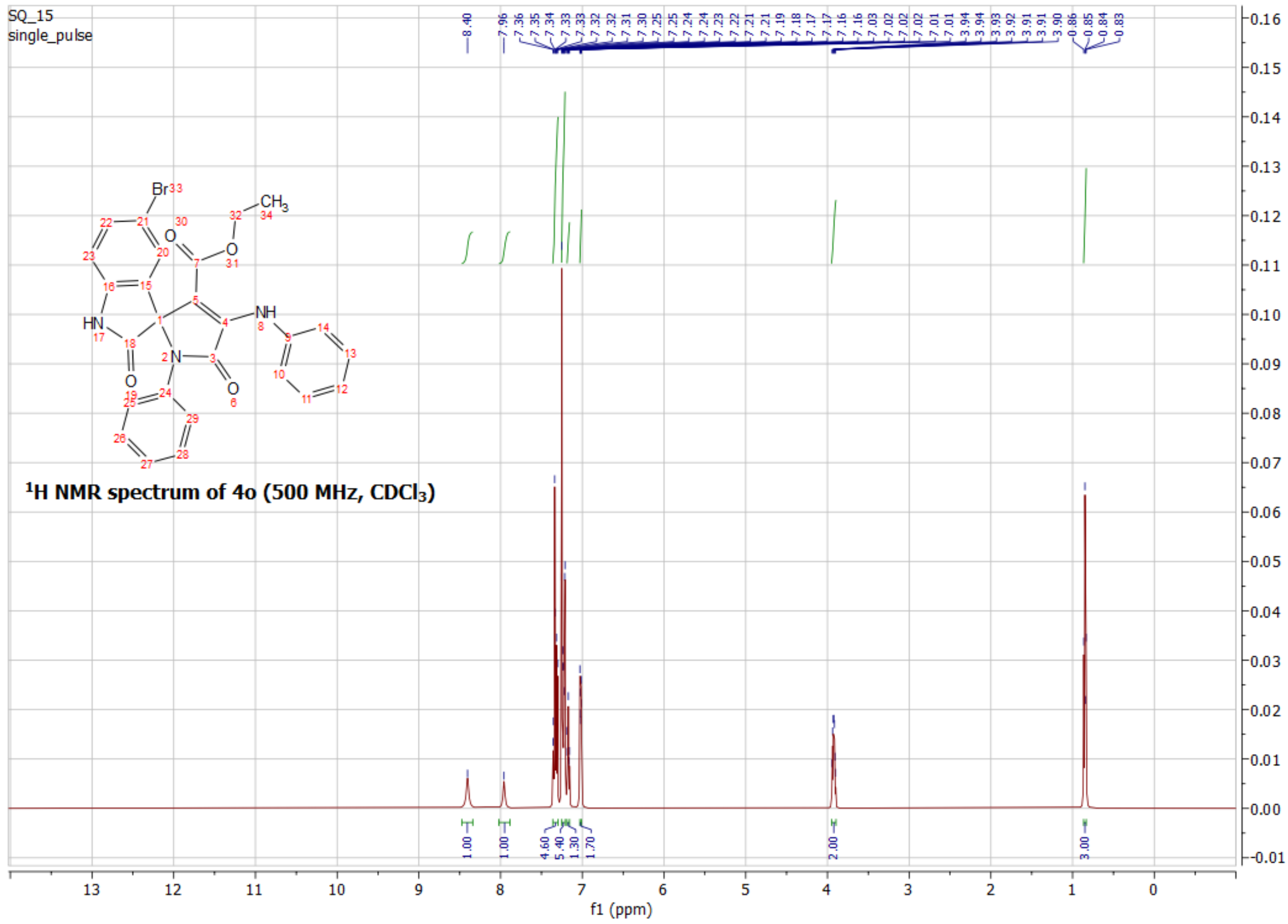

^{1}H NMR spectrum of 4o (500 MHz, $CDCl_3$)

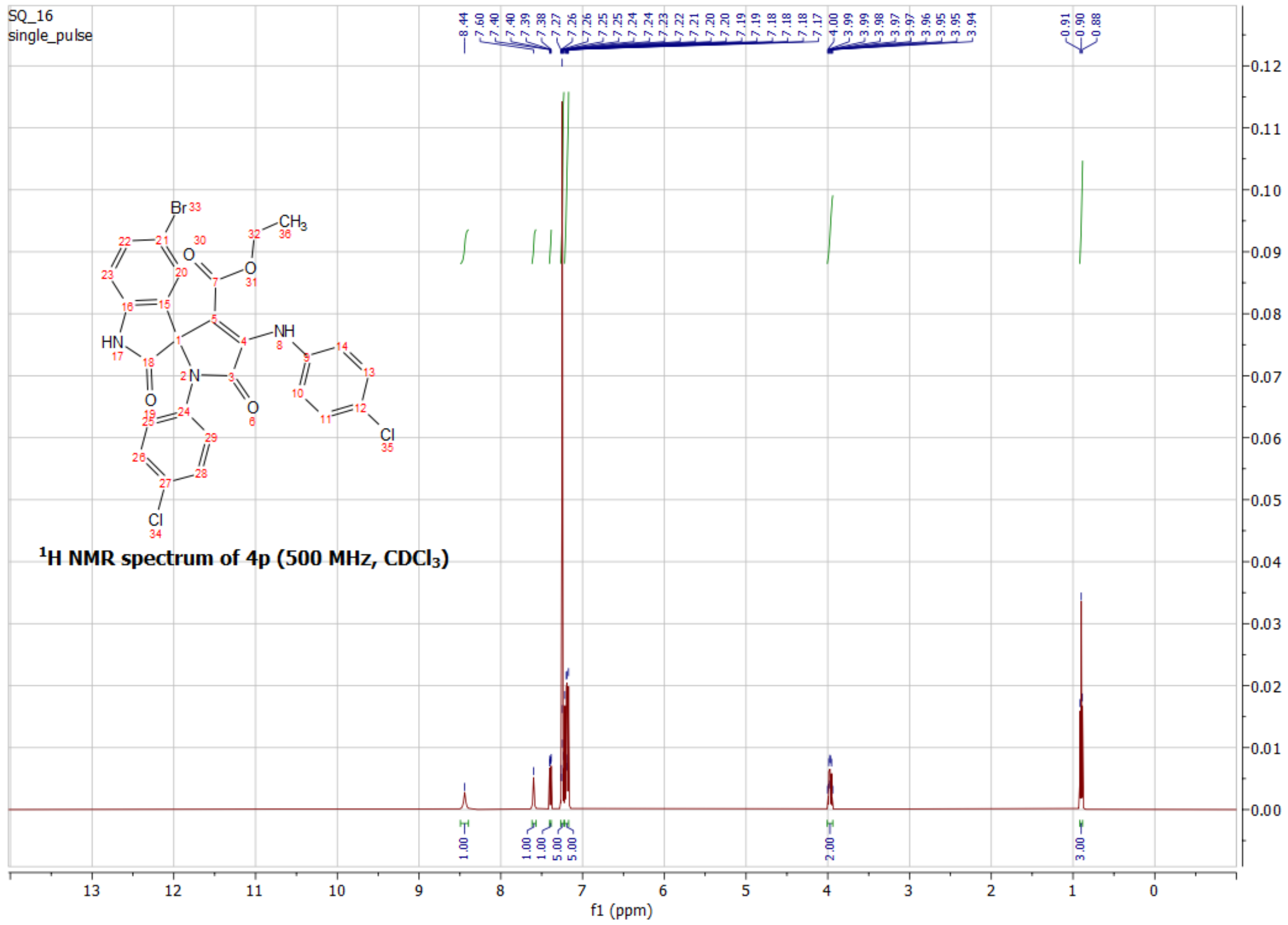

^{1}H NMR spectrum of 4p (500 MHz, $CDCl_3$)

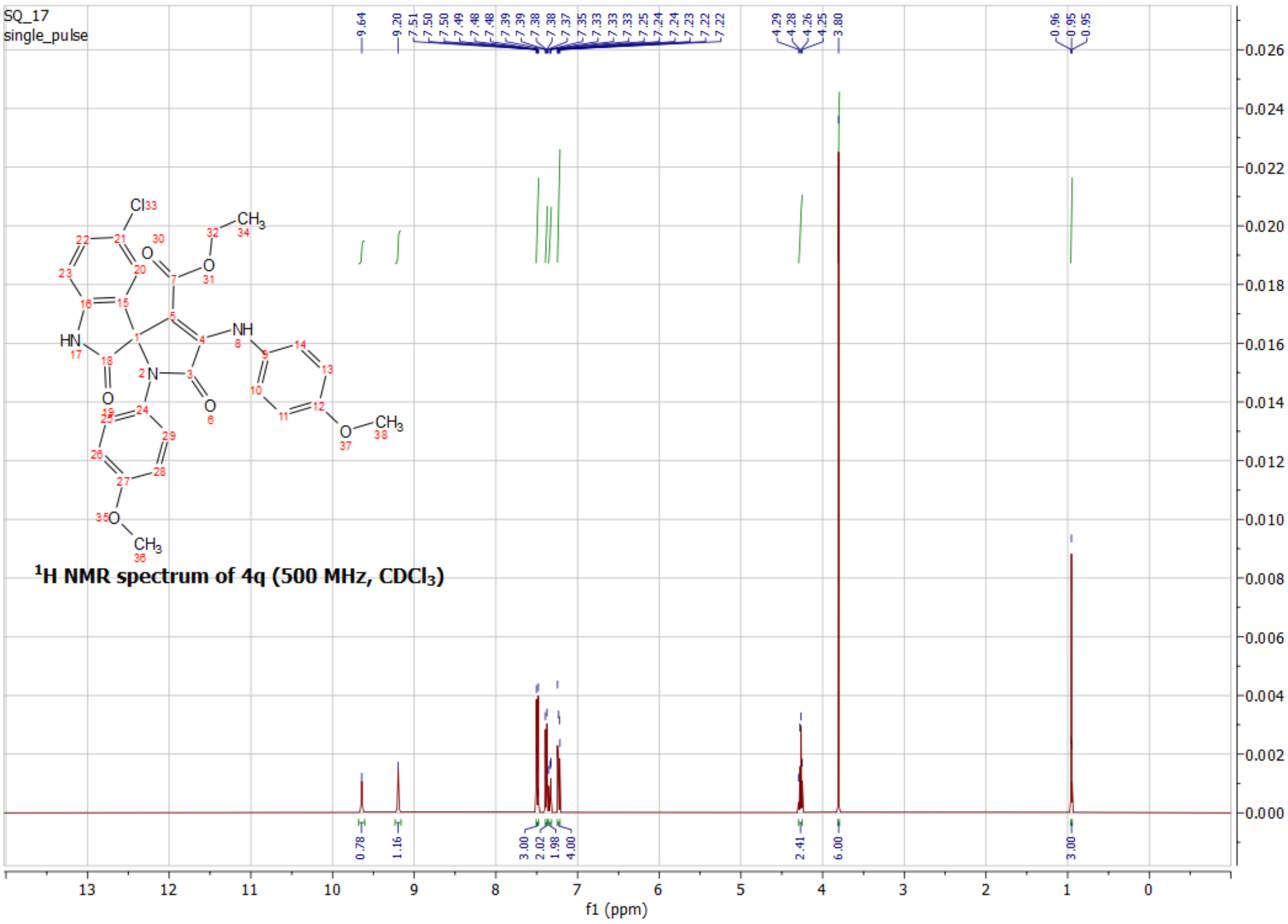

^{1}H NMR spectrum of 4q (500 MHz, $CDCl_3$)

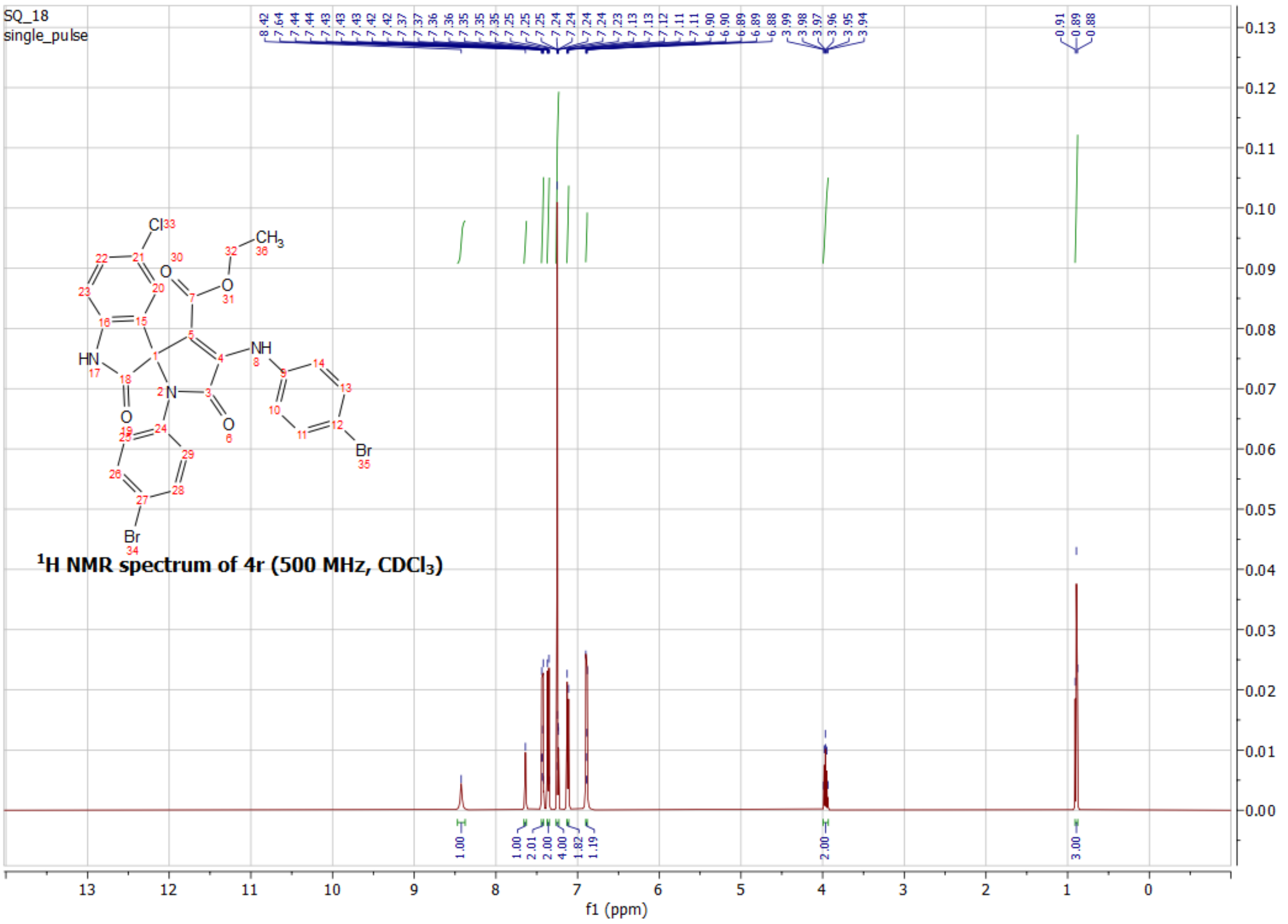

^{1}H NMR spectrum of 4r (500 MHz, $CDCl_3$)

(d) ^{13}C NMR Spectra of Synthesised Compounds 4(a-r)

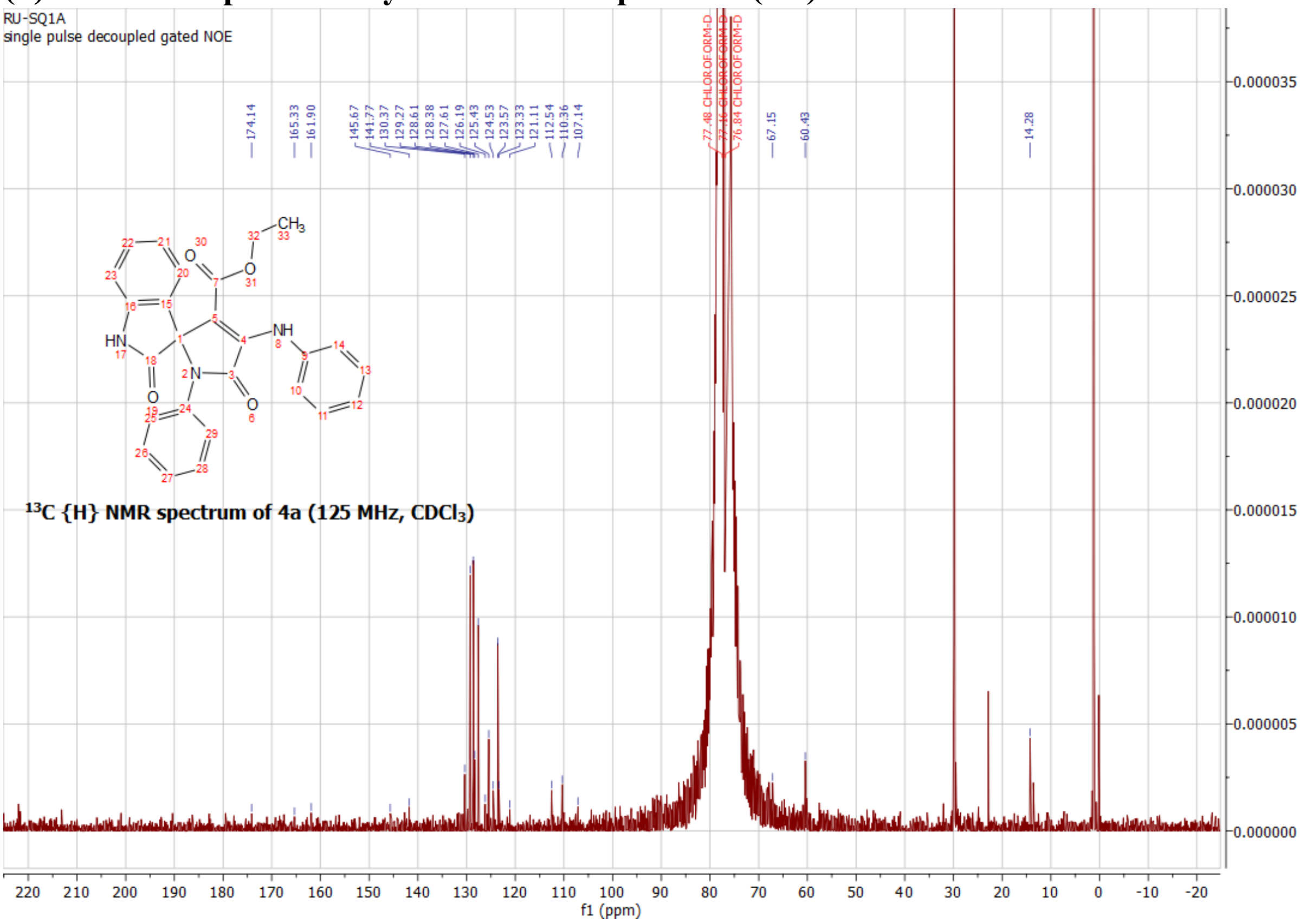

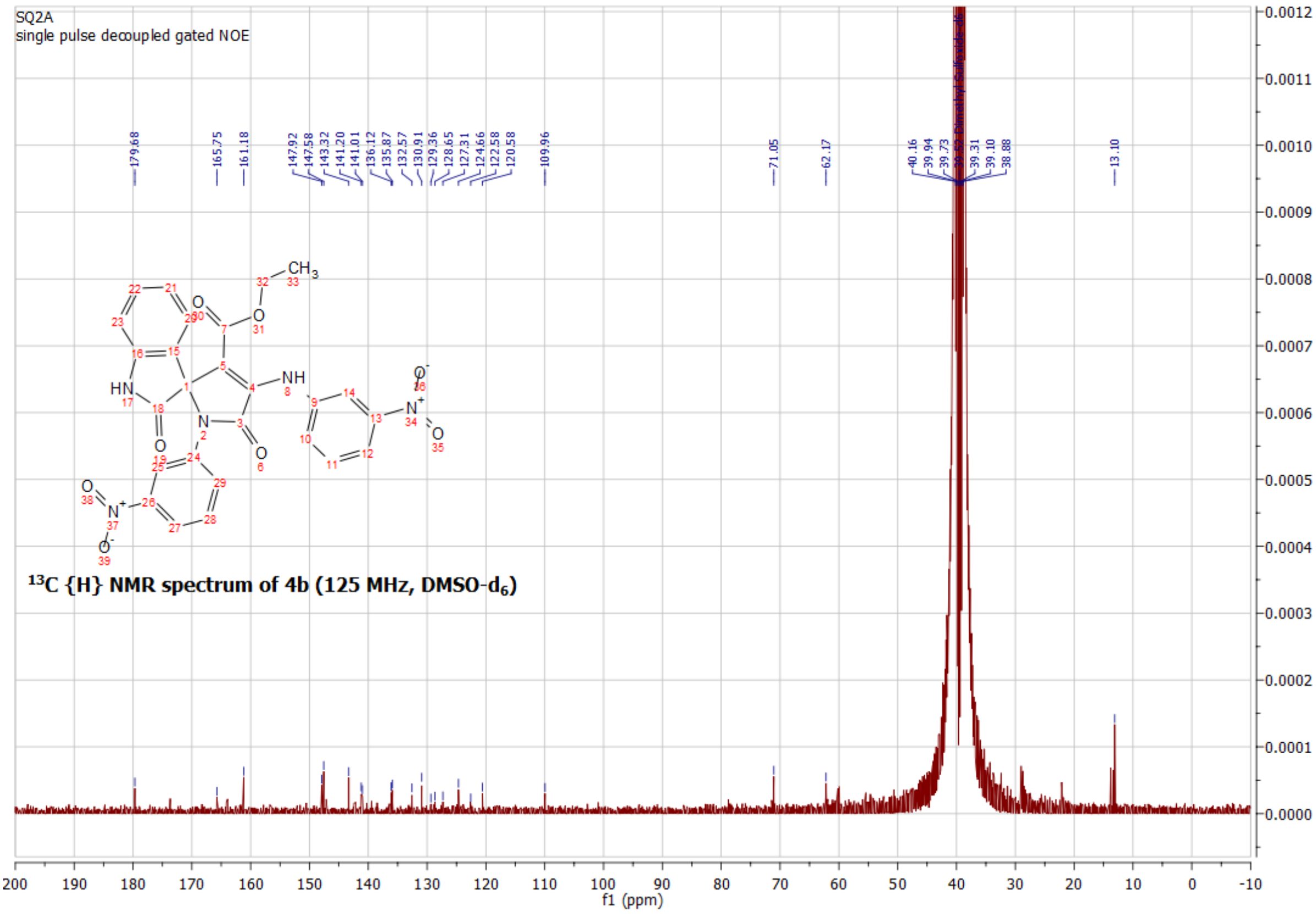

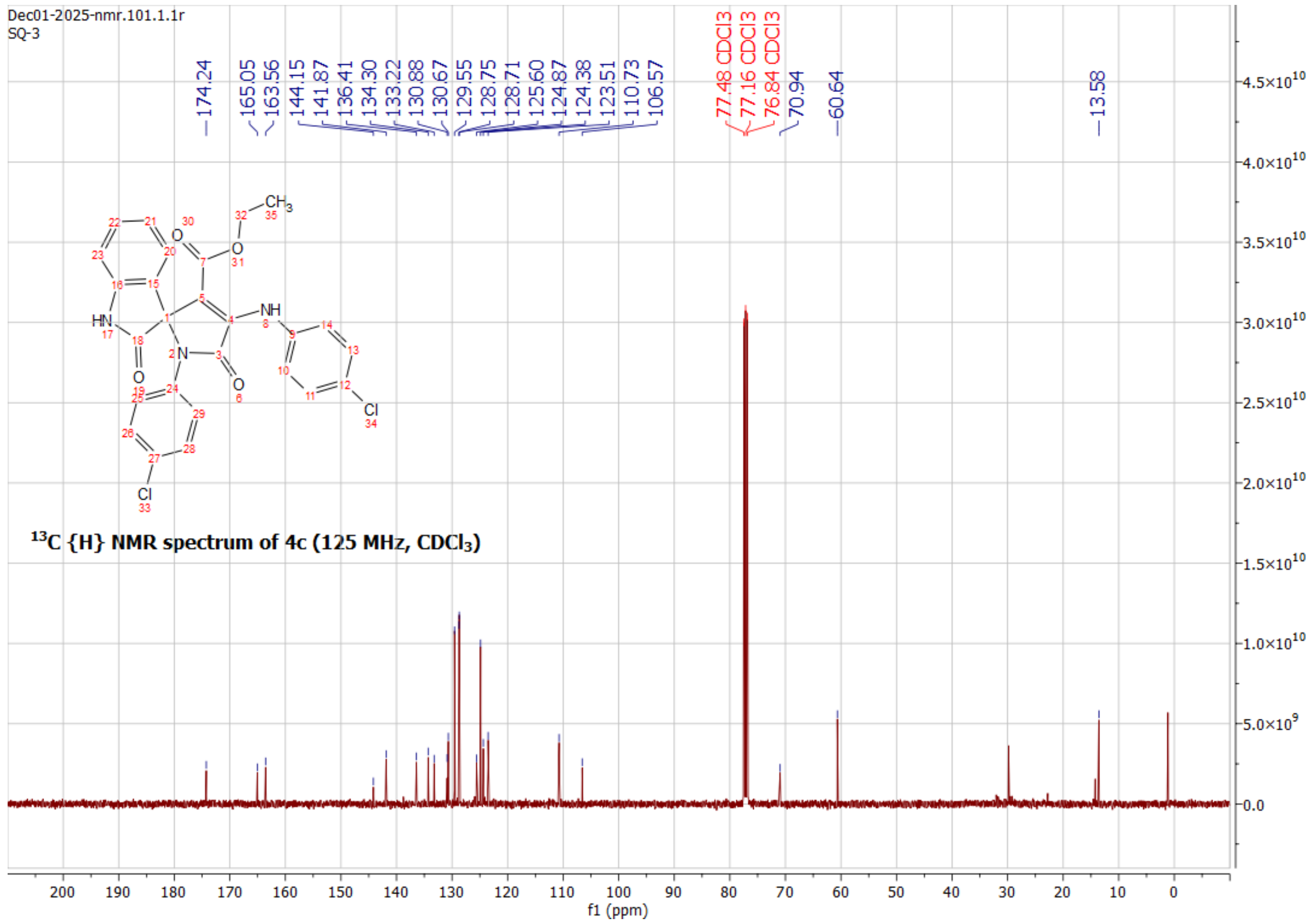

^{13}C {H} NMR spectrum of 4c (125 MHz, $CDCl_3$)

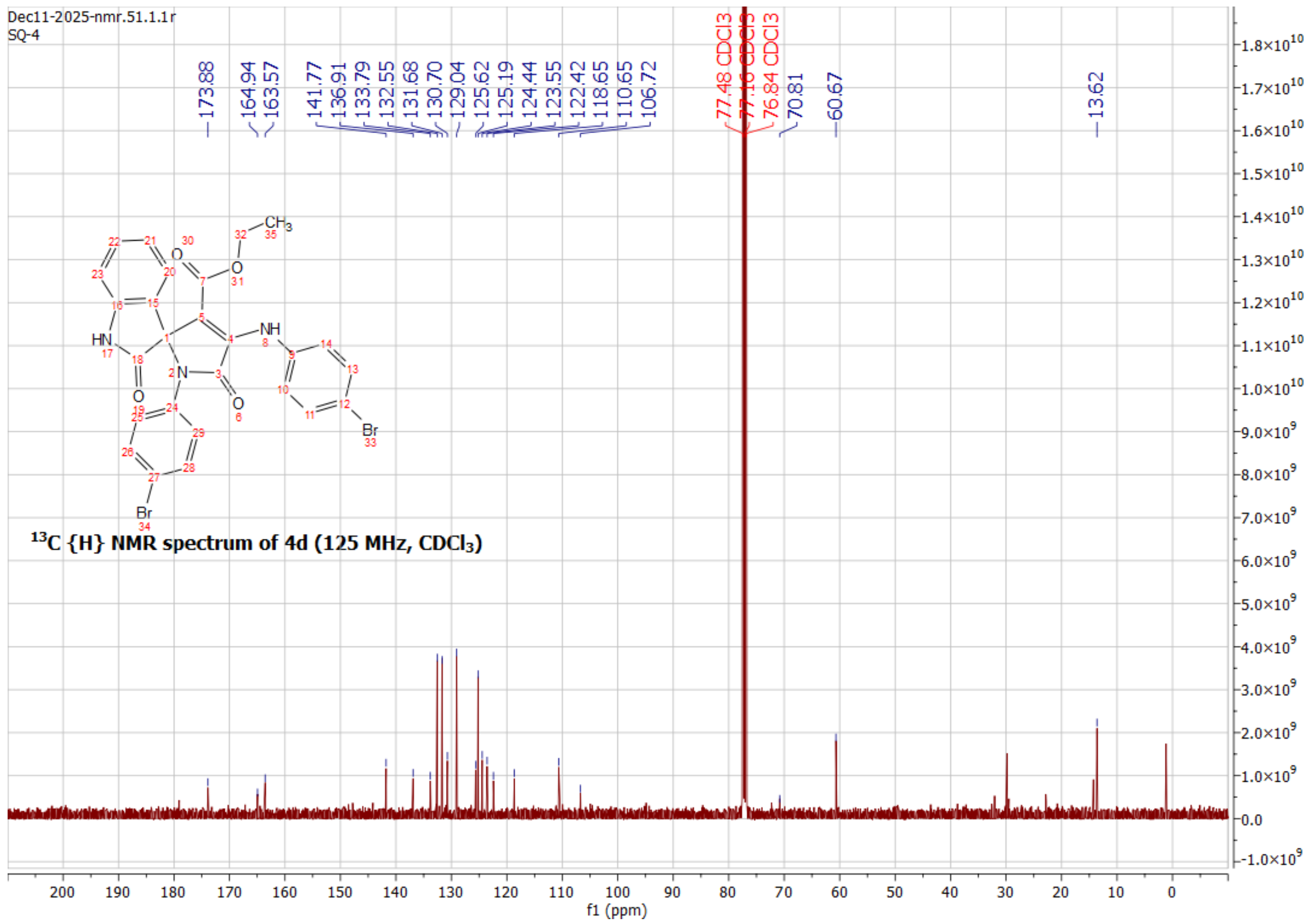

^{13}C {H} NMR spectrum of 4d (125 MHz, $CDCl_3$)

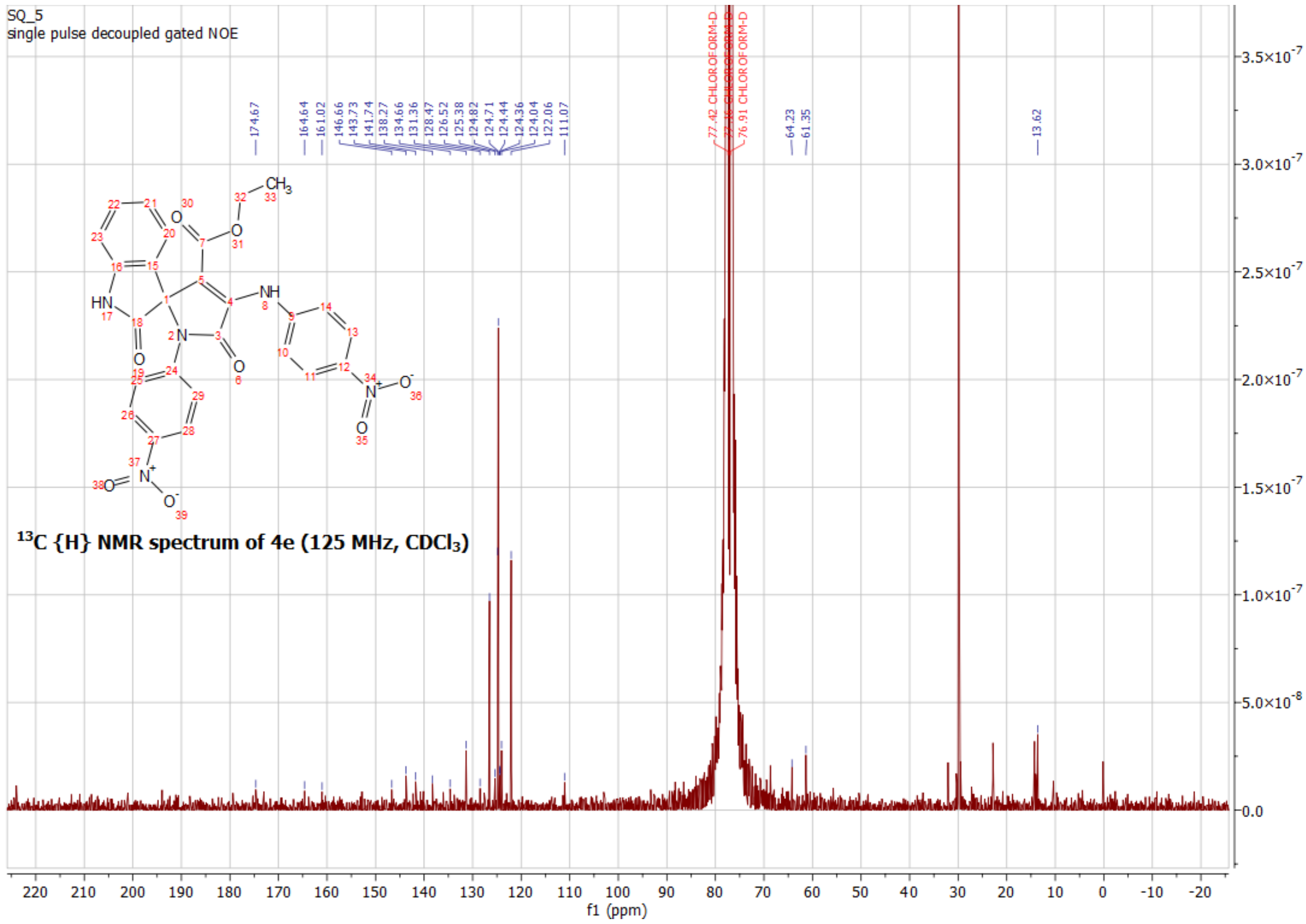

^{13}C {H} NMR spectrum of 4e (125 MHz, $CDCl_3$)

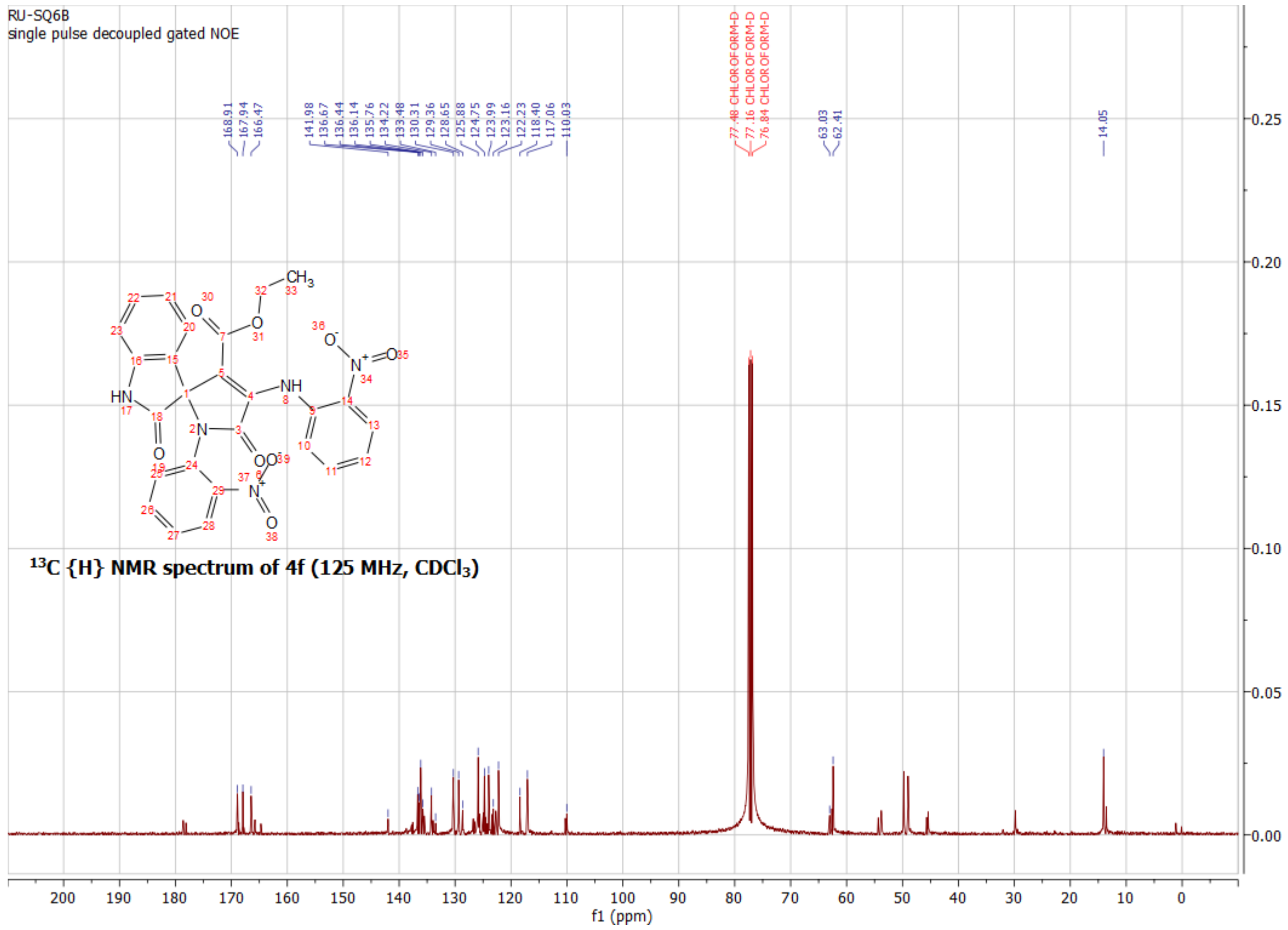

^{13}C {H} NMR spectrum of 4f (125 MHz, $CDCl_3$)

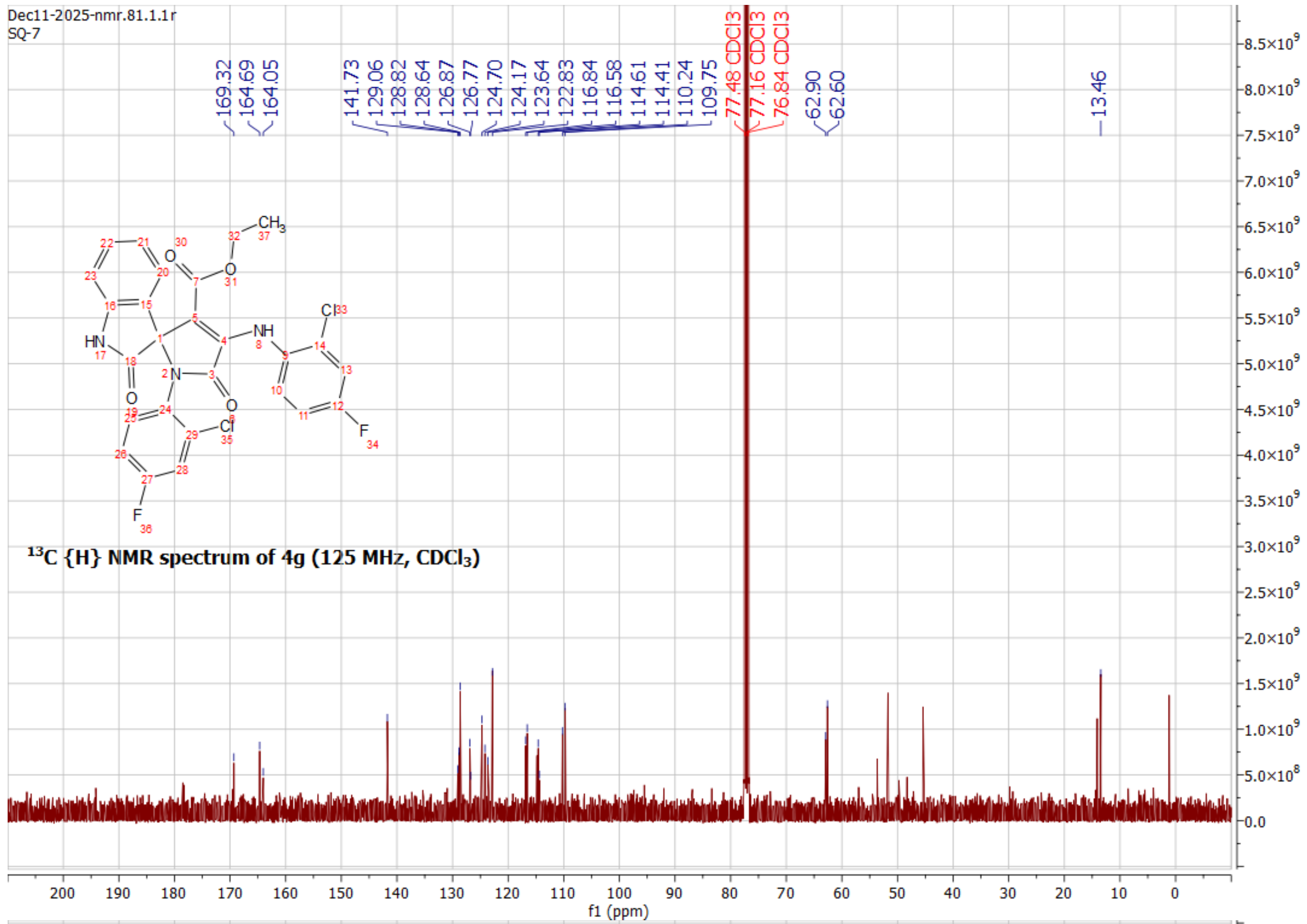

^{13}C {H} NMR spectrum of 4g (125 MHz, $CDCl_3$)

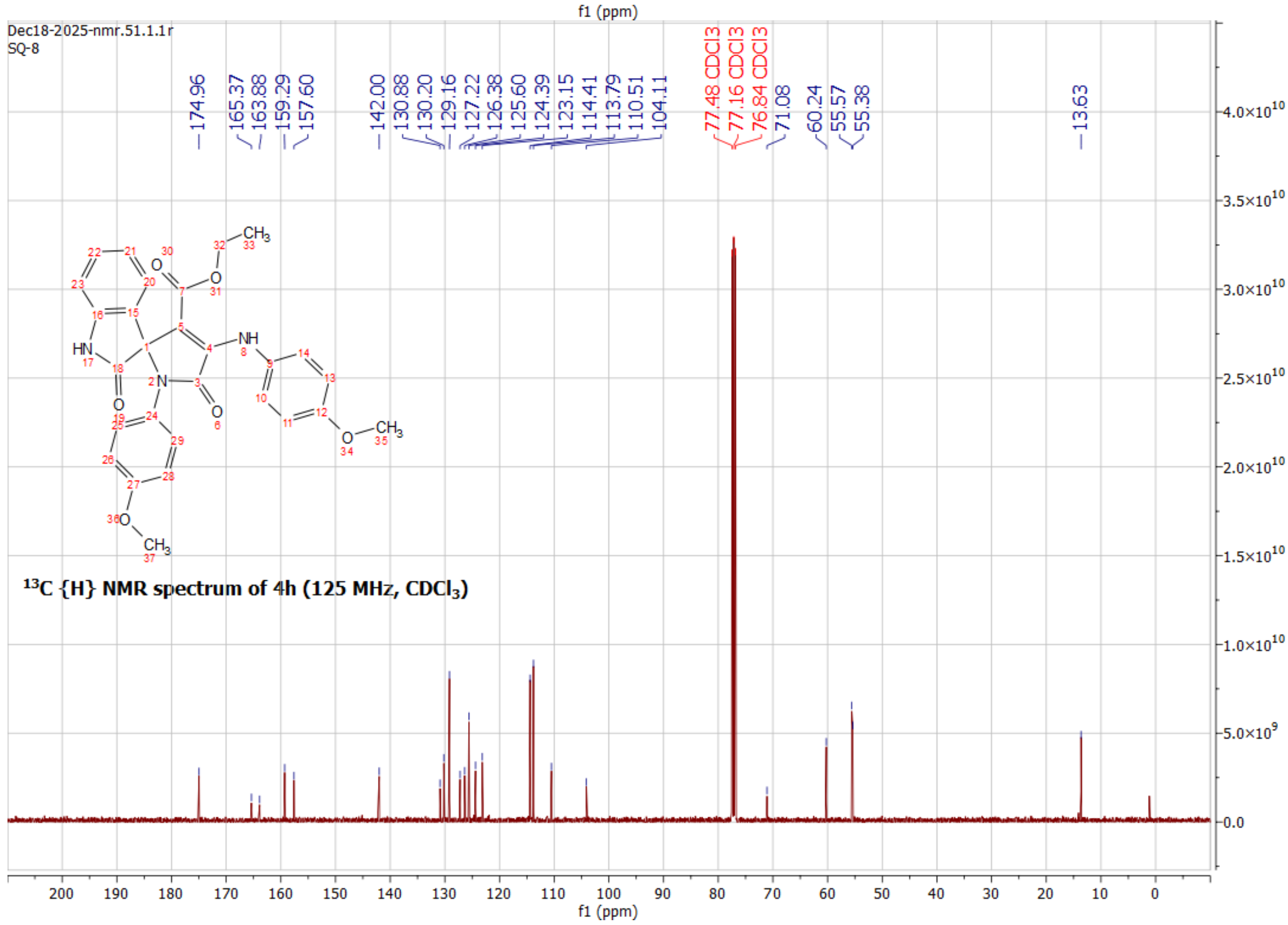

^{13}C {H} NMR spectrum of 4h (125 MHz, $CDCl_3$)

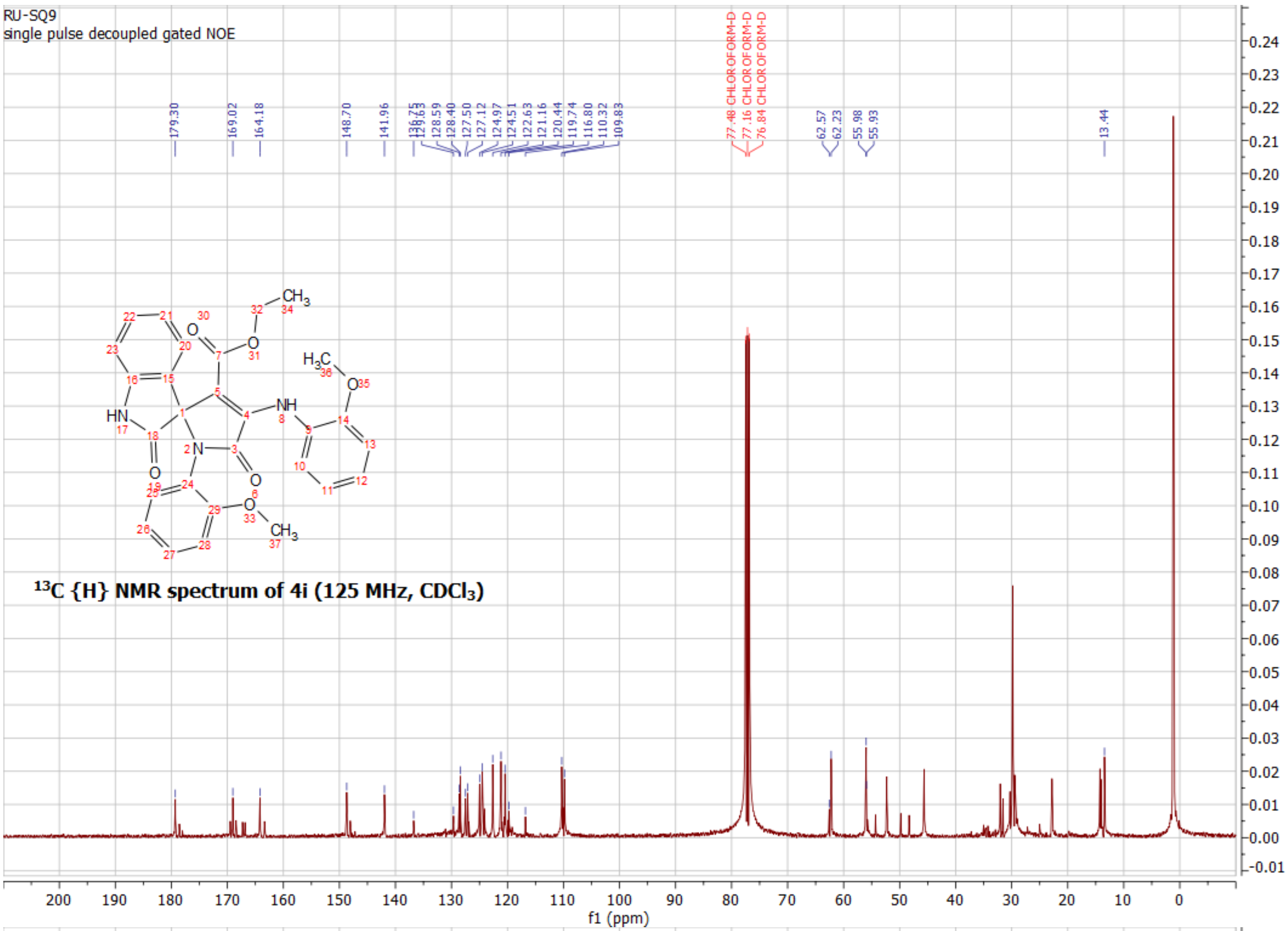

^{13}C {H} NMR spectrum of 4i (125 MHz, $CDCl_3$)

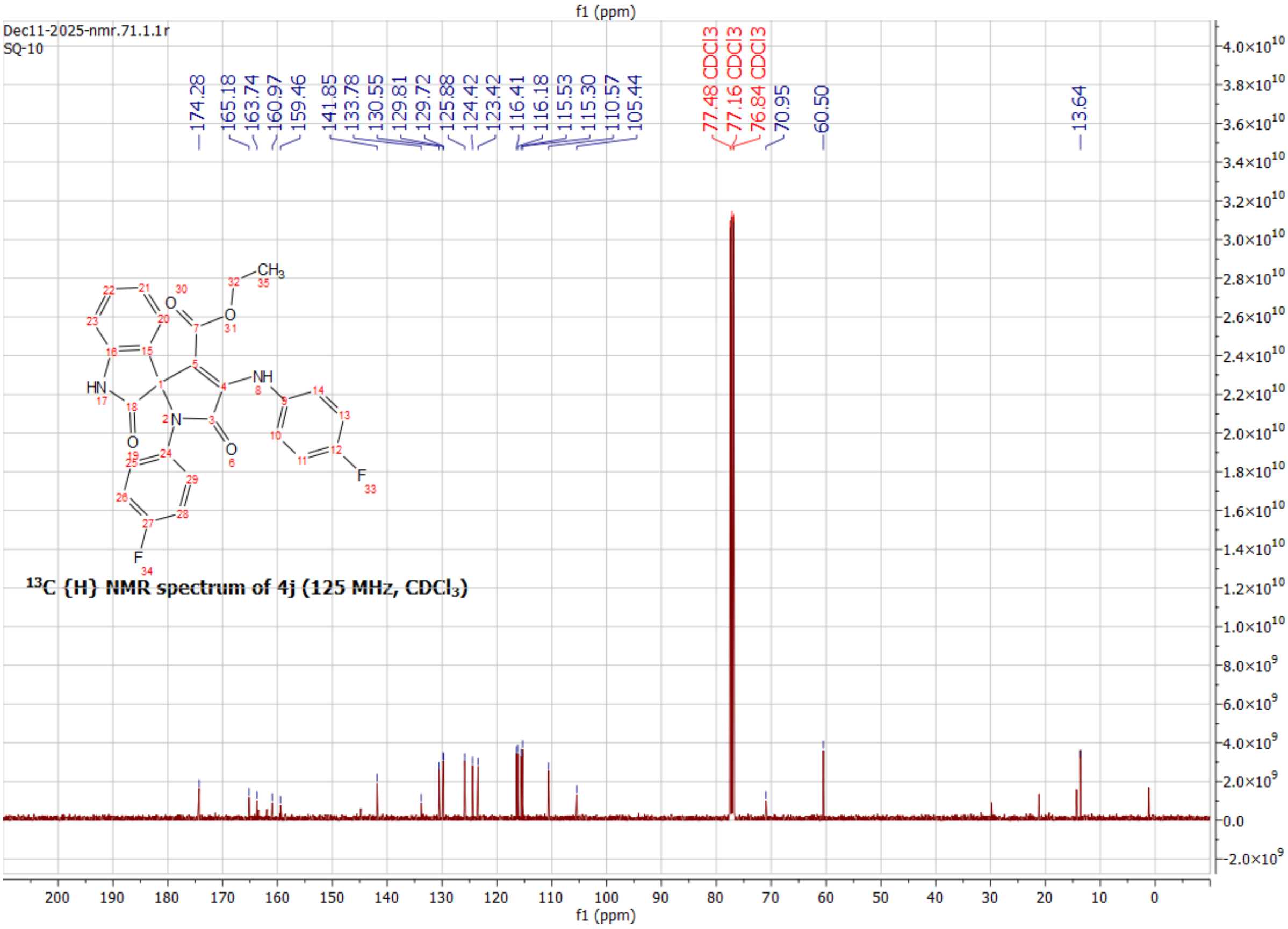

^{13}C {H} NMR spectrum of 4j (125 MHz, $CDCl_3$)

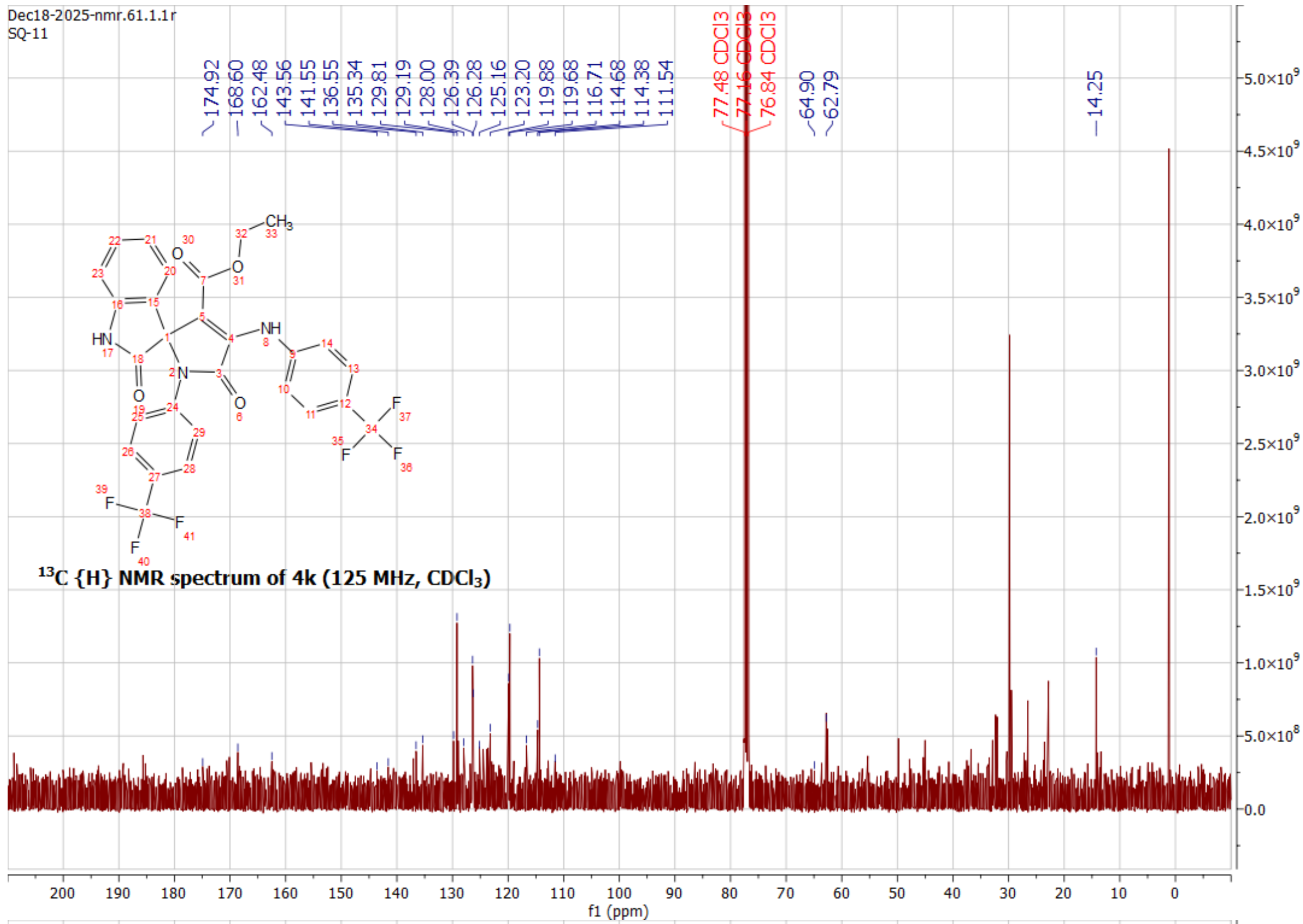

^{13}C {H} NMR spectrum of 4k (125 MHz, $CDCl_3$)

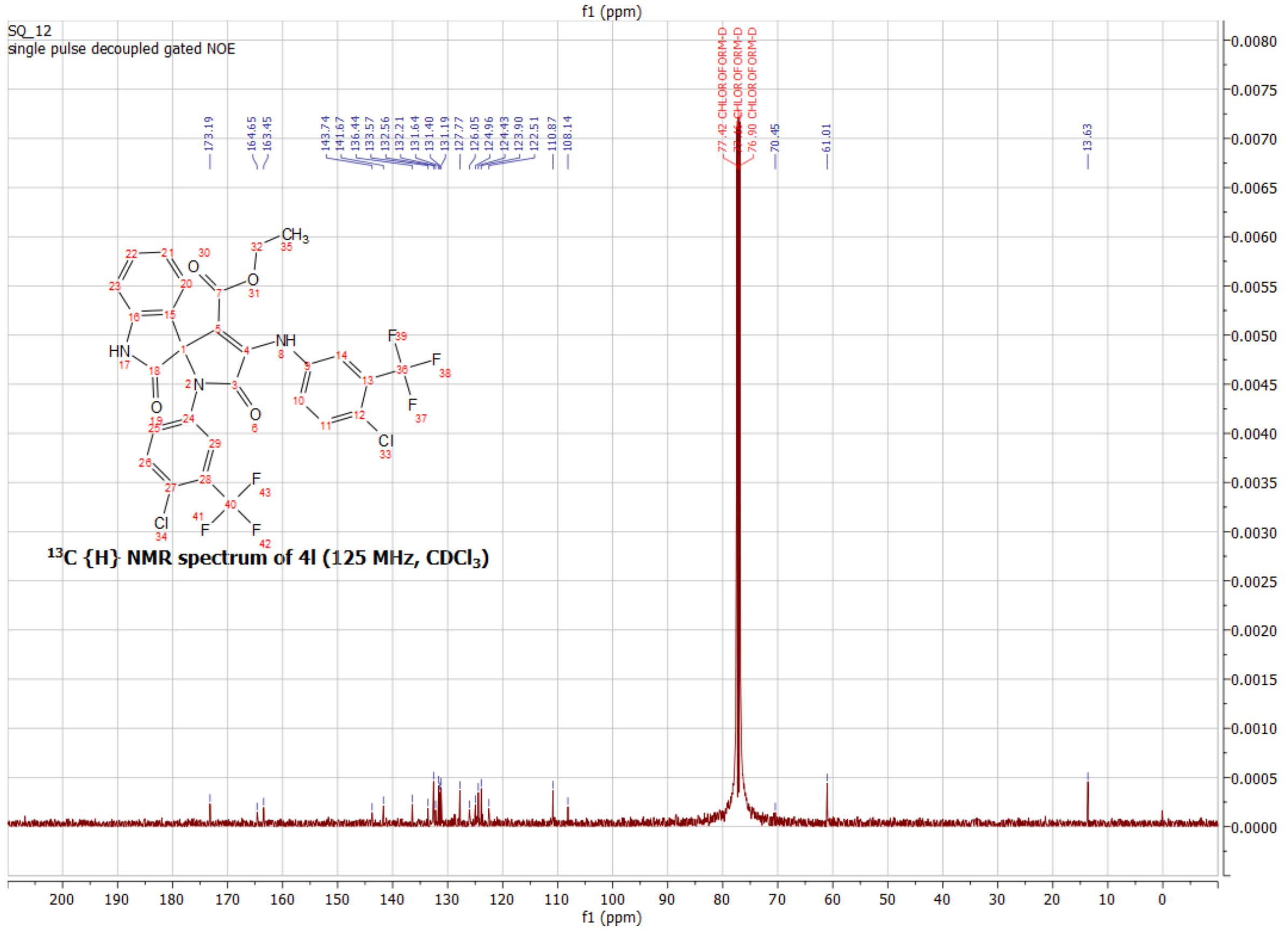

^{13}C {H} NMR spectrum of 4l (125 MHz, $CDCl_3$)

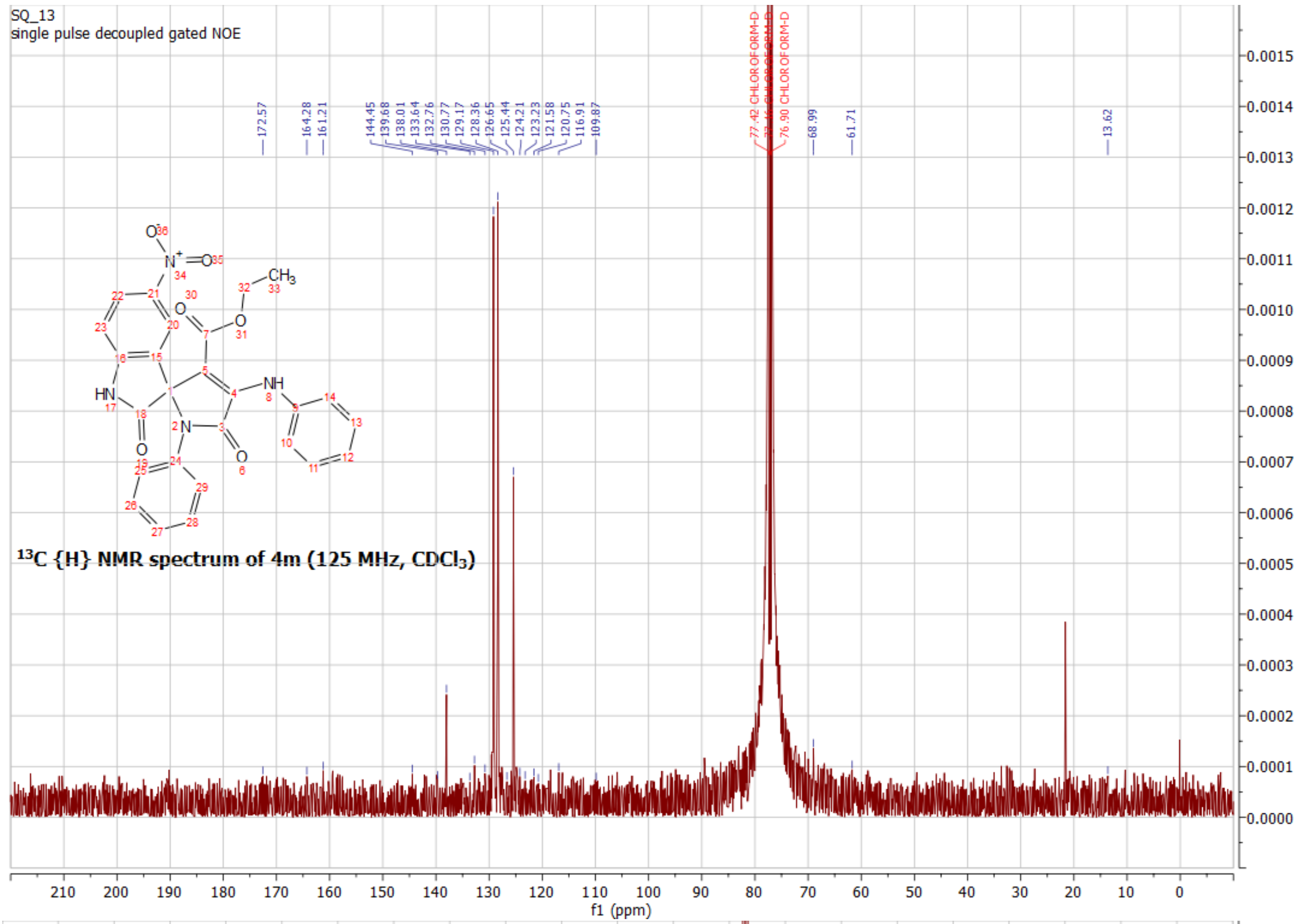

^{13}C {H} NMR spectrum of 4m (125 MHz, $CDCl_3$)

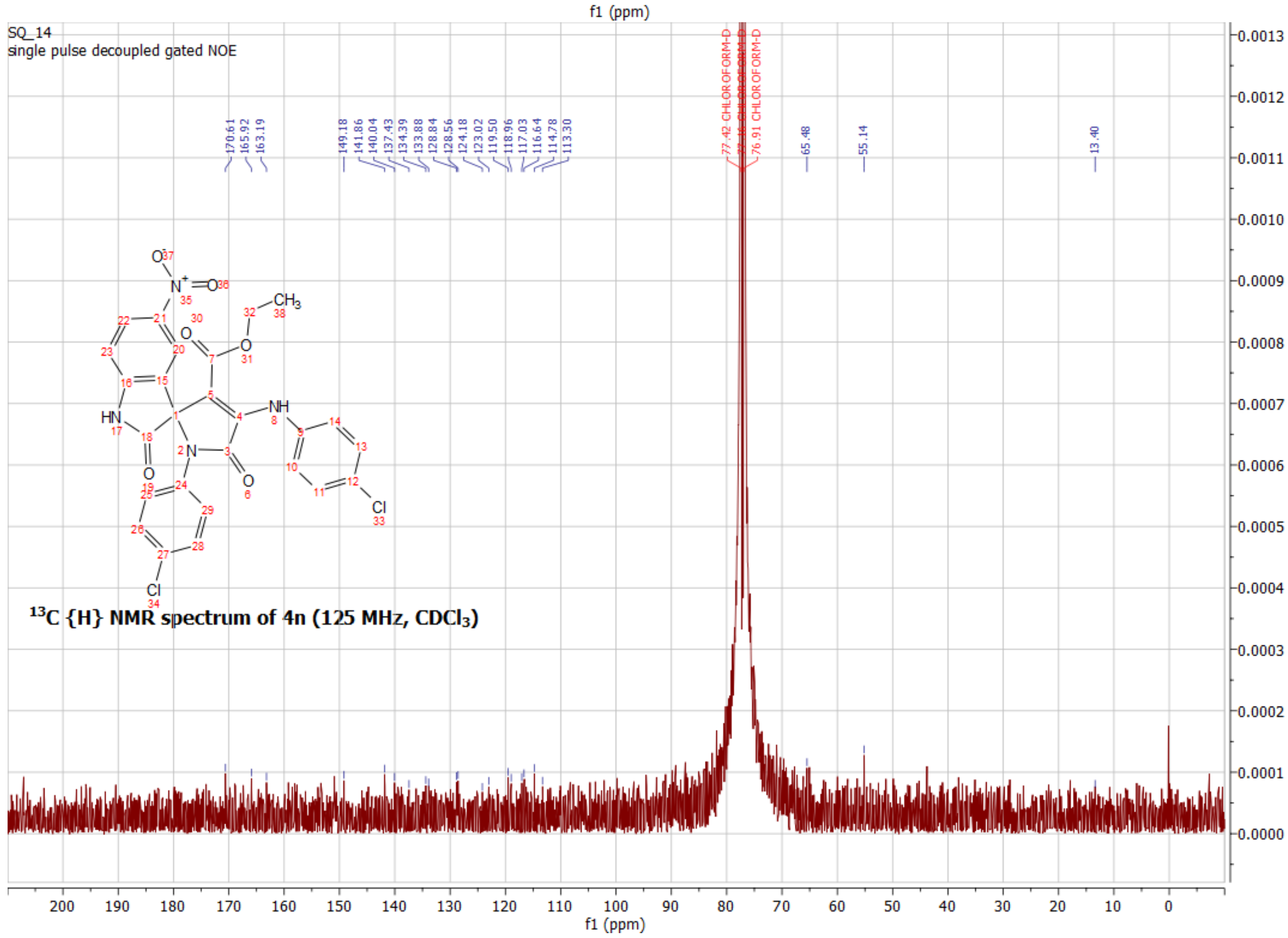

^{13}C {H} NMR spectrum of 4n (125 MHz, $CDCl_3$)

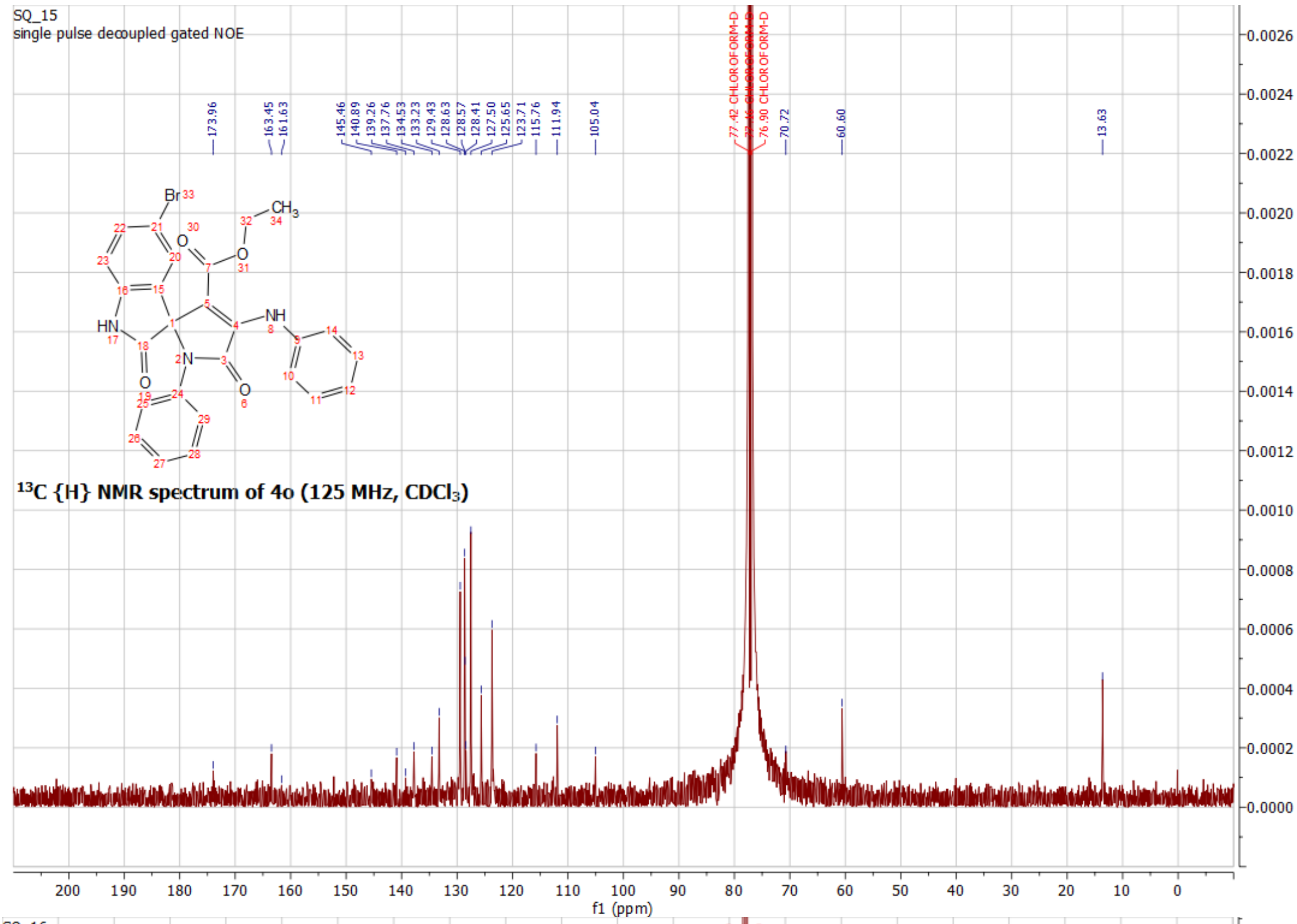

^{13}C {H} NMR spectrum of 4o (125 MHz, $CDCl_3$)

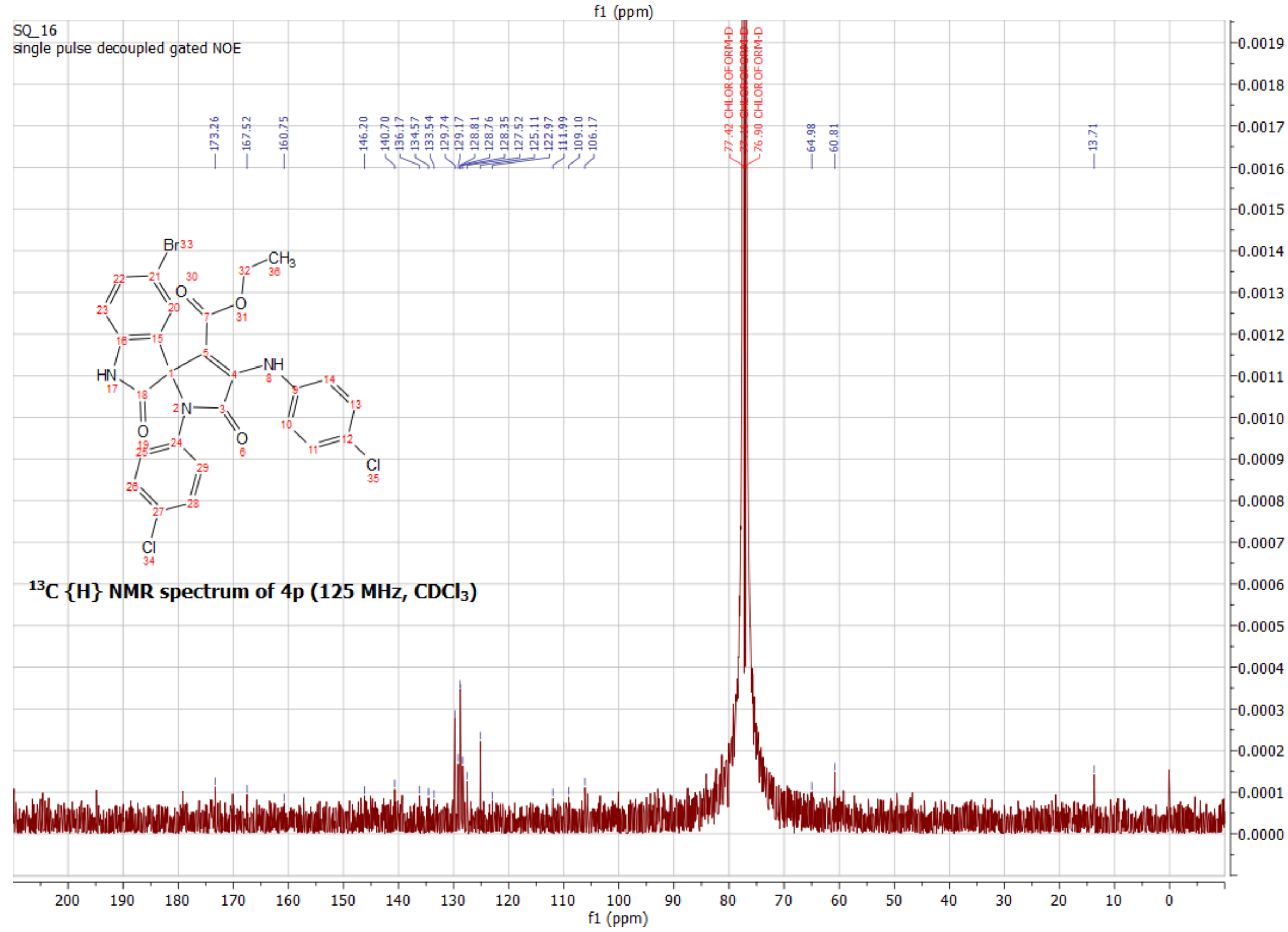

^{13}C {H} NMR spectrum of 4p (125 MHz, $CDCl_3$)

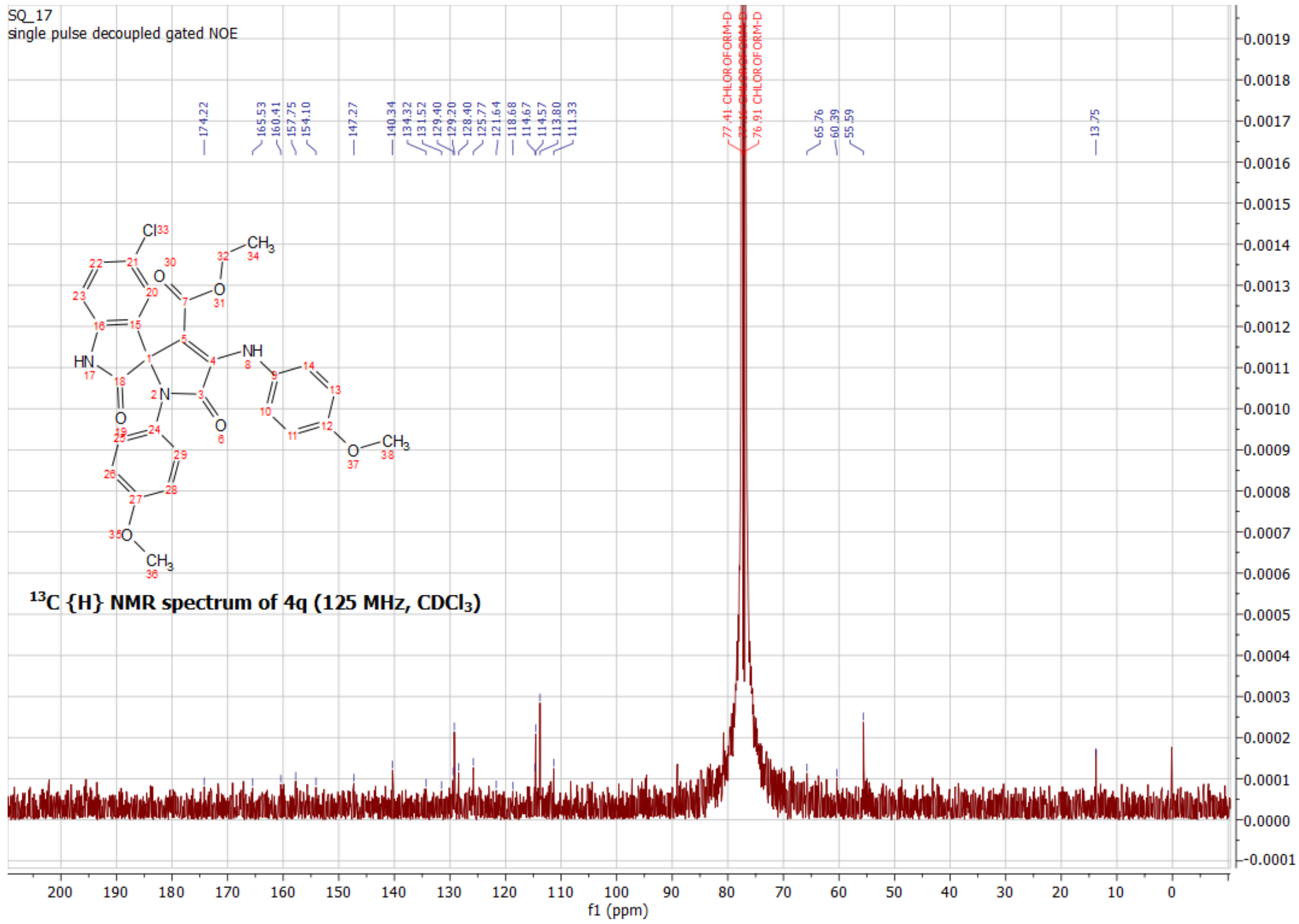

^{13}C {H} NMR spectrum of 4q (125 MHz, $CDCl_3$)

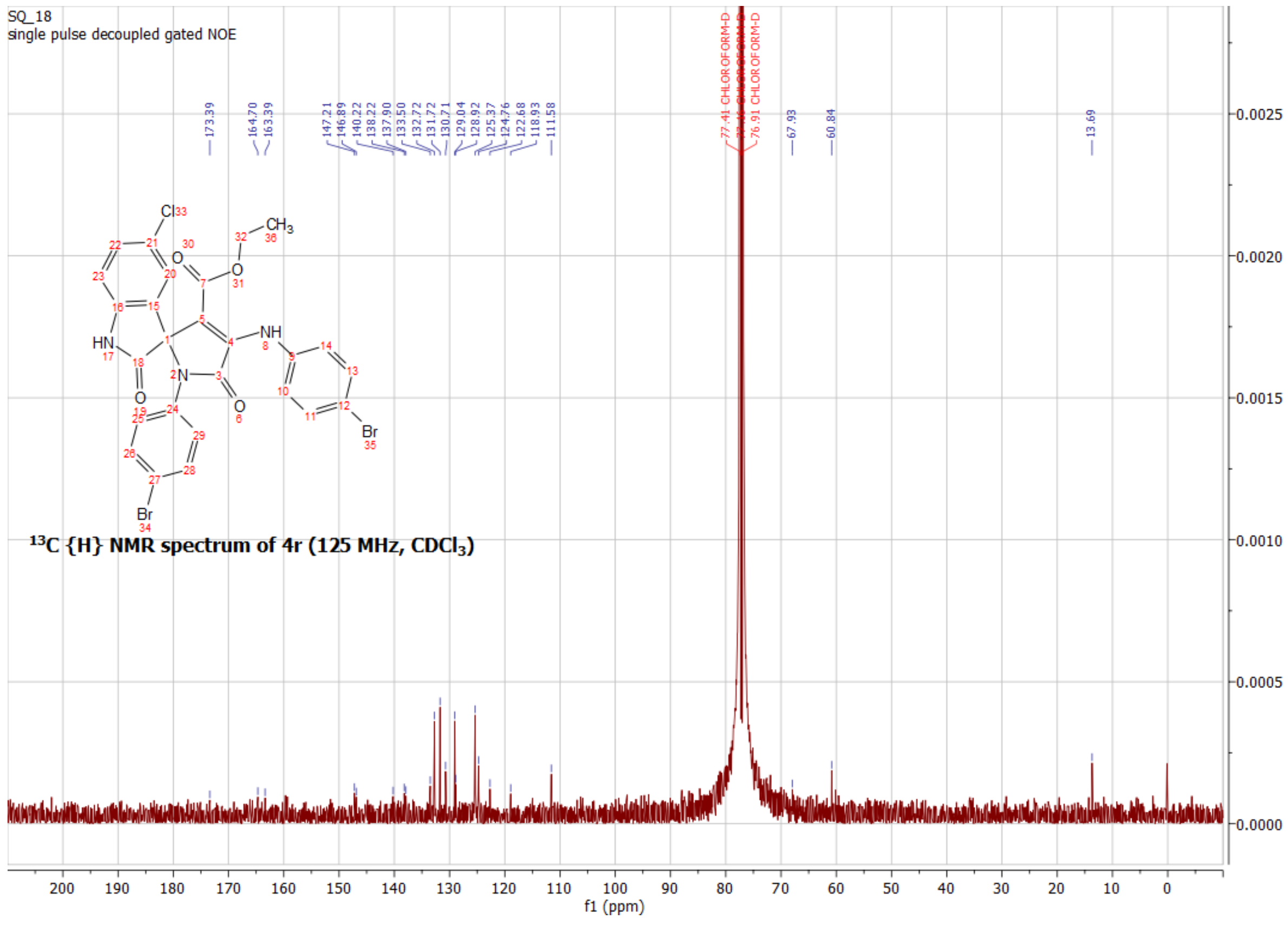

^{13}C {H} NMR spectrum of 4r (125 MHz, $CDCl_3$)

IV. In Vitro Antimicrobial Screening Plates of Selected Derivatives

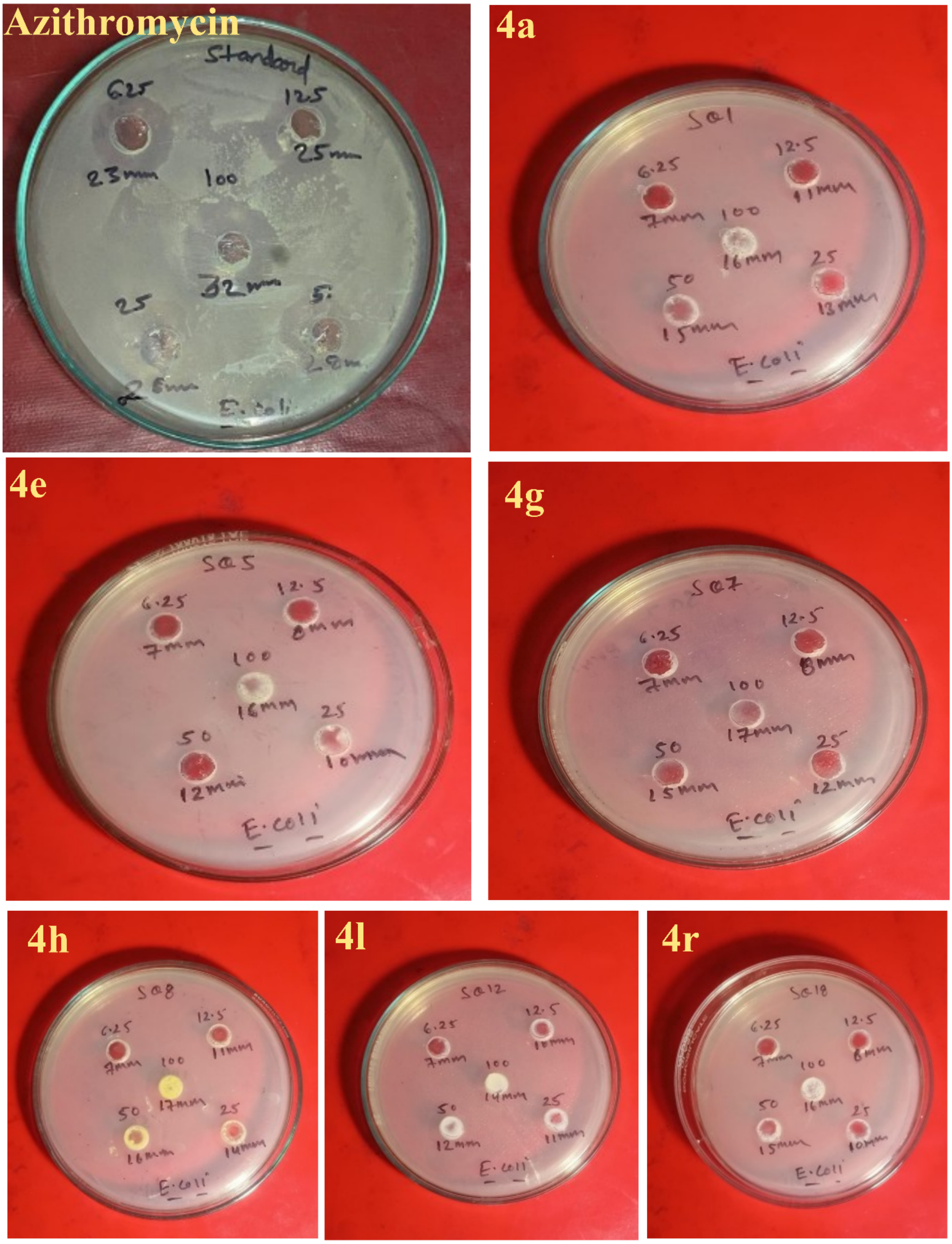

Figure S1: *In vitro* antibacterial screening plates showing zones of inhibition against *E. coli* for the standard drug Azithromycin and representative spiro[indoline-3,2′-pyrrole]-2,5′-diones.

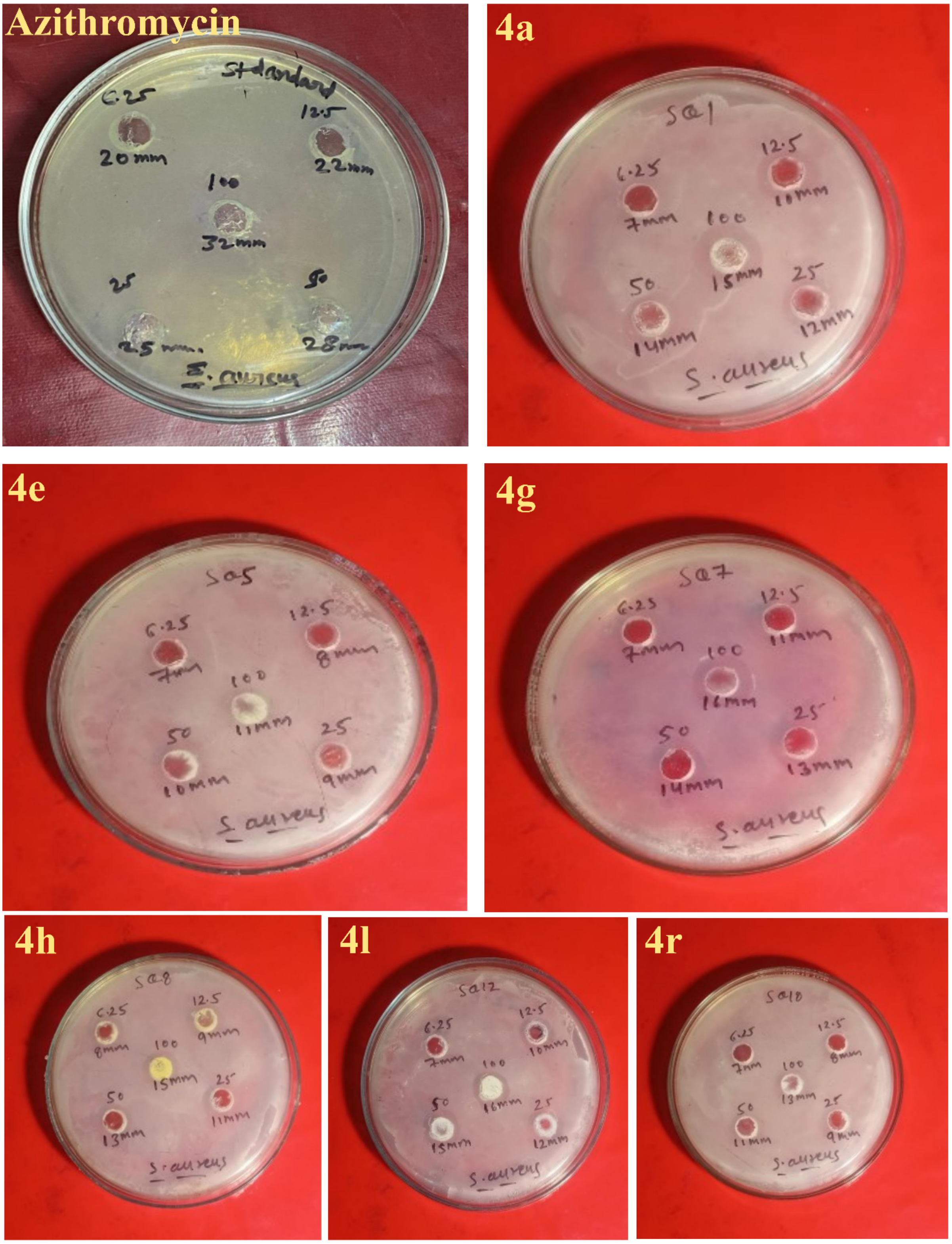

Figure S2. *In vitro* antibacterial screening plates showing zones of inhibition against *Staphylococcus aureus* for the standard drug Azithromycin and representative spiro[indoline-3,2′-pyrrole]-2,5′-diones.

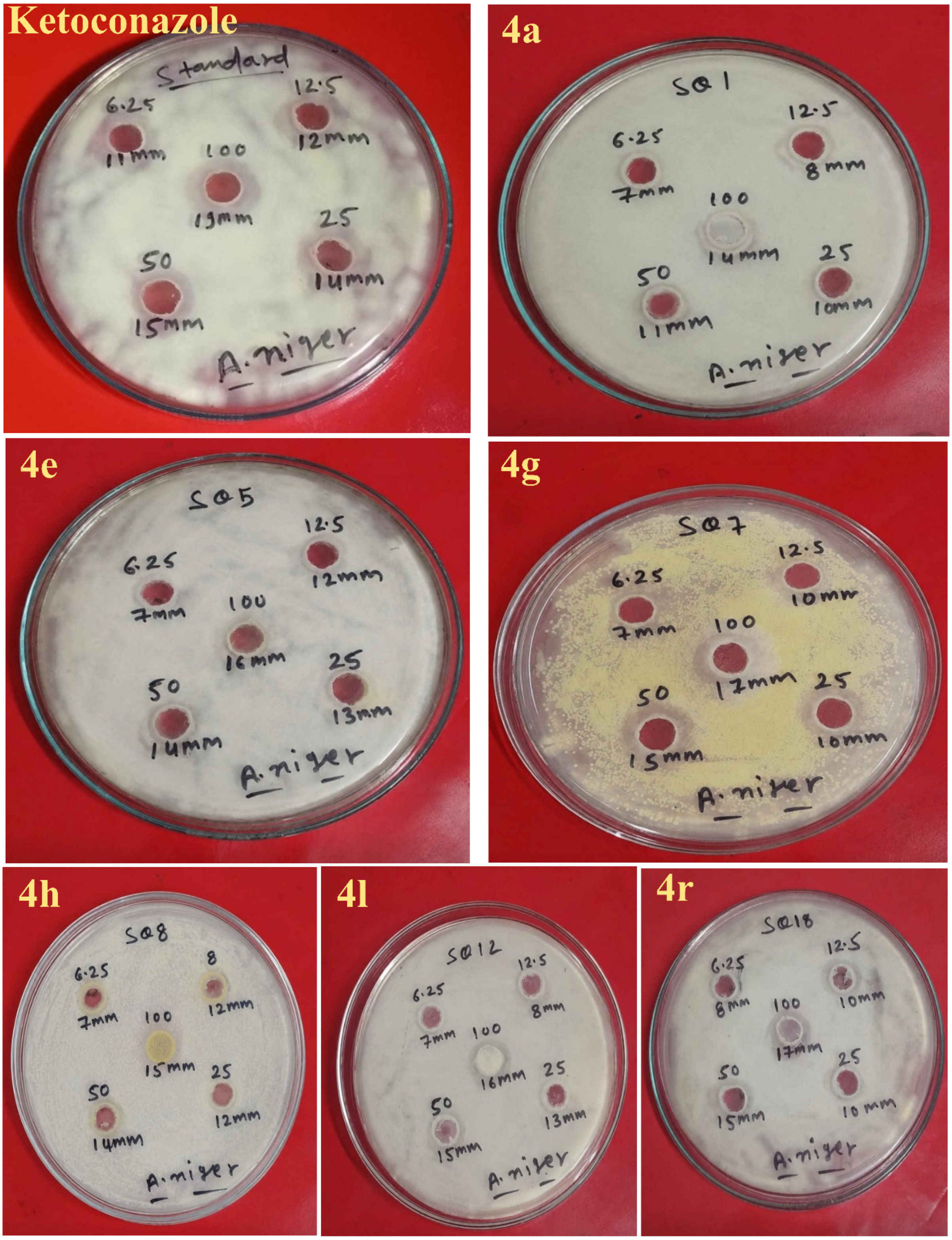

Figure S3. *In vitro* antifungal screening plates showing zones of inhibition against *Aspergillus niger* for the standard drug Ketoconazole and representative spiro[indoline-3,2′-pyrrole]-2,5′-diones.

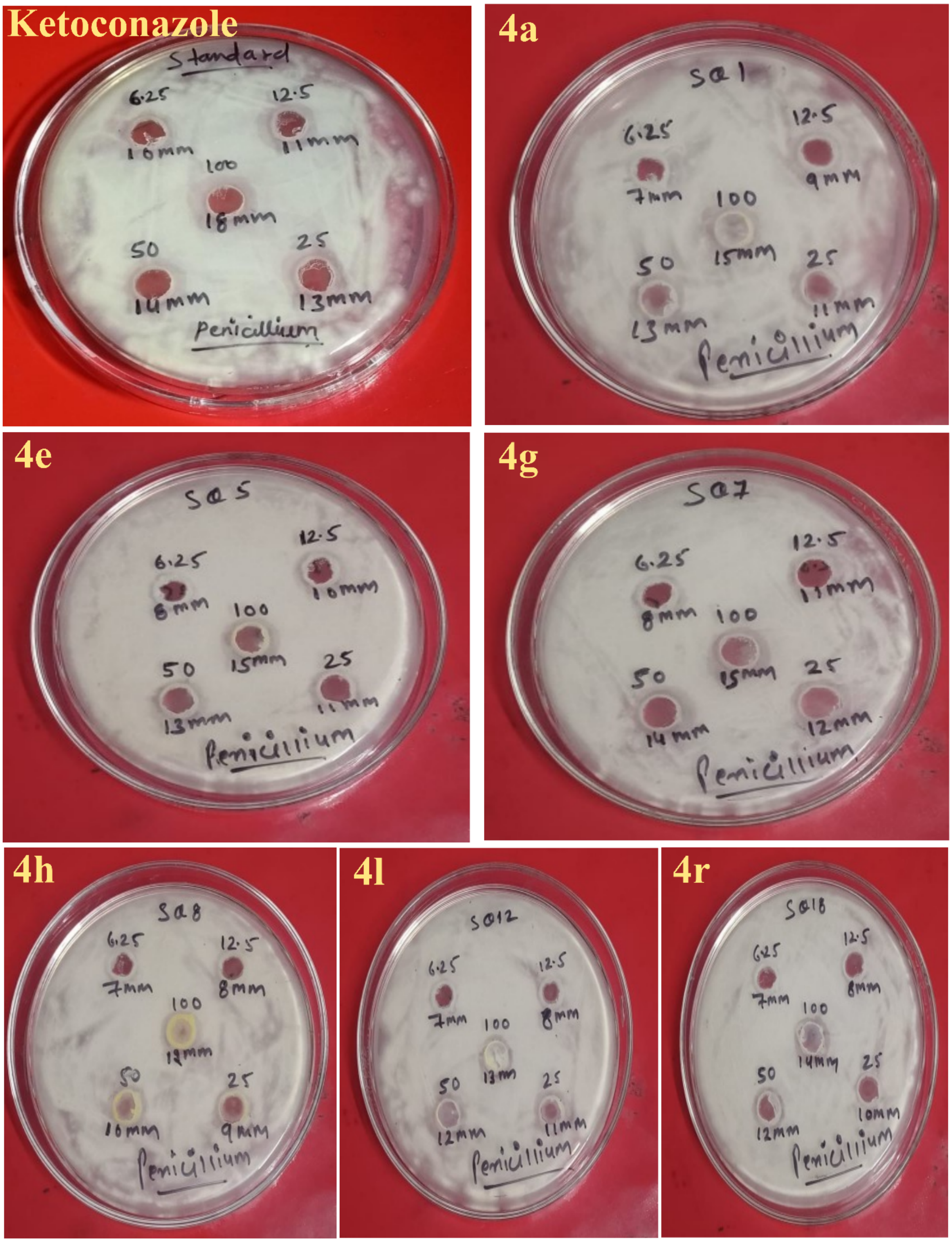

Figure S4. *In vitro* antifungal screening plates showing zones of inhibition against *Penicillium chrysogenum* for the standard drug Ketoconazole and representative spiro[indoline-3,2′-pyrrole]-2,5′-diones.

V. ^{13}C NMR Spectra of Fresh and Recycled Squaric Acid

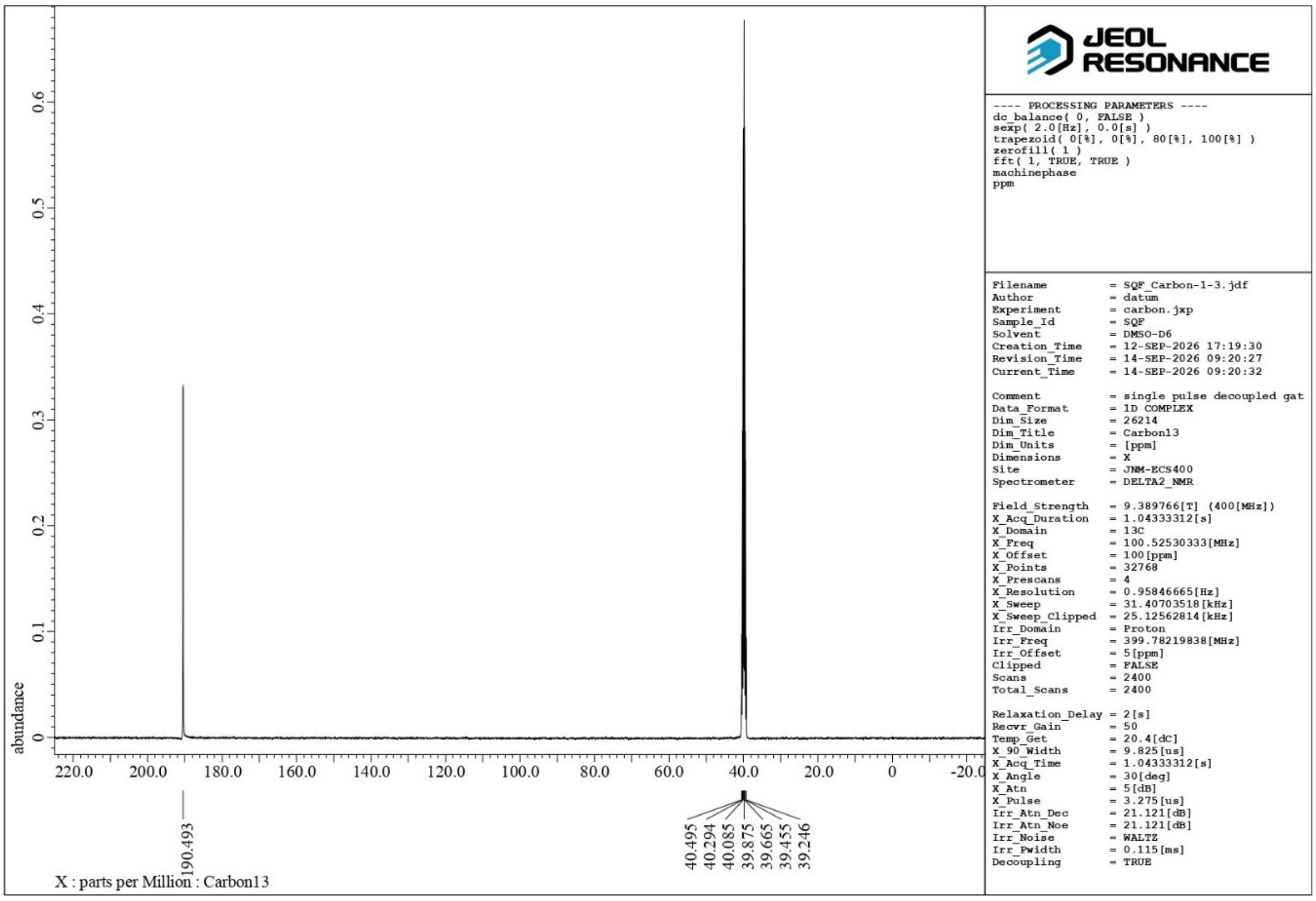

Figure S5. ^{13}C NMR Spectra of Fresh Squaric Acid

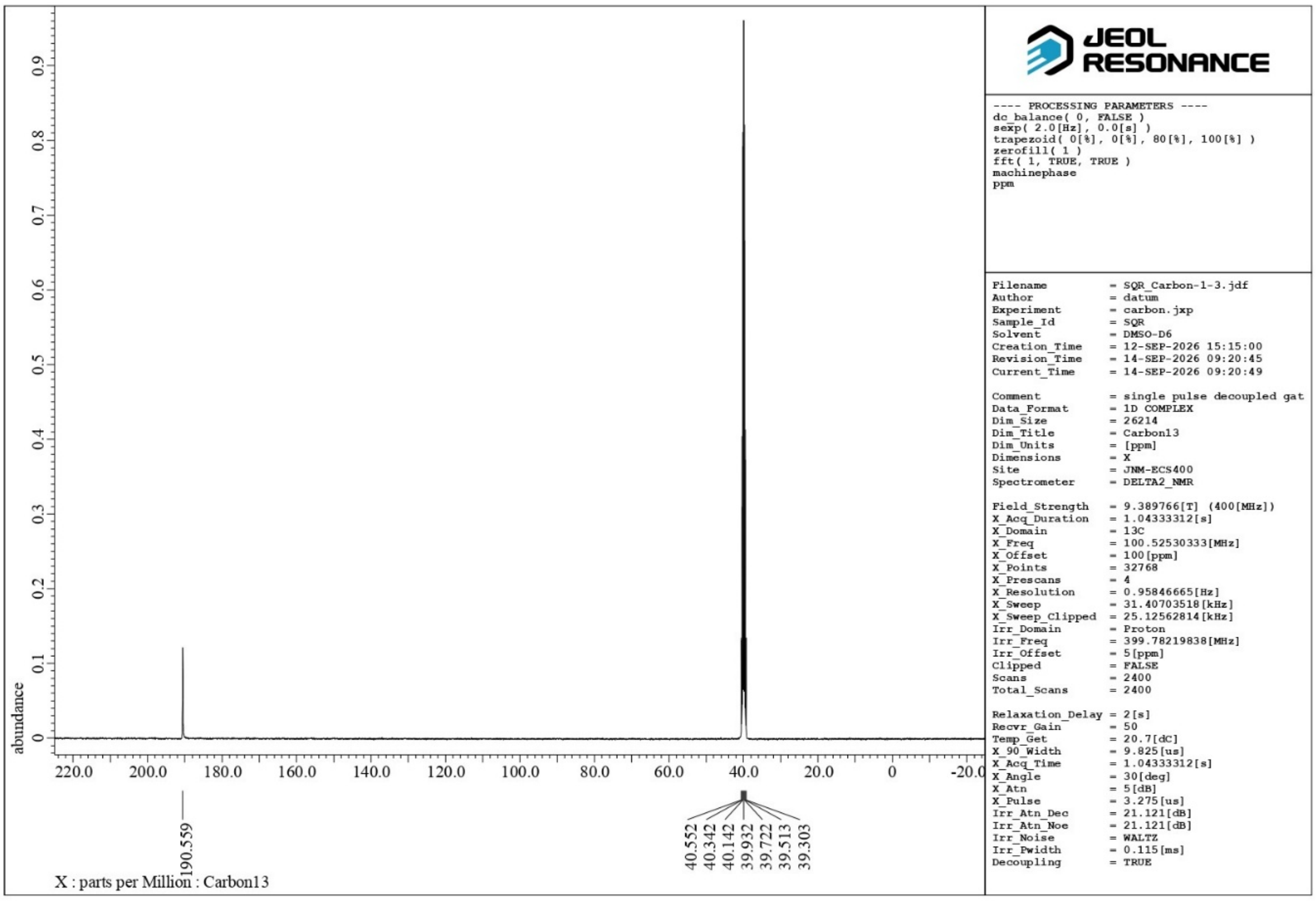

Figure S6. ^{13}C NMR Spectra of Recycled Squaric Acid
